# Supplementary material for: Tracing active members in microbial communities by BONCAT and click chemistry-based enrichment of newly synthesized proteins
Source: ISME Commun. 2024 Dec 4;4(1):ycae153. doi: 10.1093/ismeco/ycae153 (PMC11683836; doi:10.1093/ismeco/ycae153)
Supplement: Genome_Server_ycae153 [file genome_server_ycae153.zip › Genome Server/BIN24_TYGS_job_results.pdf]

TYPE (STRAIN) GENOME SERVER

JOB SUMMARY

PRINT DATE:	2024-06-16 16:15:53 +0200
JOB ID:	bf75185e-22ba-4e70-bd09-cccb6df88fbf
RESULT PAGE:	https://tygs.dsmz.de/user_results/show?guid=bf75185e-22ba-4e70-bd09-cccb6df88fbf

Table 1: Phylogenies

Publication-ready versions of both the genome-scale GBDP tree and the 16S rRNA gene sequence tree can be customized and exported either in SVG (vector graphic) or PNG format from within the phylogeny viewers in your TYGS result page. For publications the **SVG format is recommended** because it is lossless, always keeps its high resolution and can also be easily converted to other popular formats such as PDF or EPS. Please follow the link provided above!

Table 2: Identification

The below list contains the result of the TYGS species identification routine.

Explanation of remarks that might occur in the below table:

remark [R1]: The TYGS type strain database is automatically updated on an almost daily basis. However, if a particular type strain genome is not available in the TYGS database, this can have several reasons which are detailed in the FAQ. You can request an extended 16S rRNA gene analysis via the 16S tree viewer found in your result page to detect **not yet genome-sequenced** type strains relevant for your study.

remark [R2]: > 70% dDDH value (formula d_4) and (almost) minimal dDDH values for gene-content formulae d_0 and d_6 indicate a potentially unreliable identification result and should thus be checked via the 16S rRNA gene sequence similarity. Such strong deviations can, in principle, be caused by sequence contamination.

remark [R3]: G+C content difference of > 1 % indicates a potentially unreliable identification result because within species G+C content varies no more than 1 %, if computed from genome sequences (PMID: 24505073).

Strain	Conclusion	Identification result	Remark
**'bin.24'**	potential new species		see [R1]

Table 3: Pairwise comparisons of user genomes vs. type-strain genomes

The following table contains the pairwise dDDH values between your user genomes and the selected type-strain genomes. The dDDH values are provided along with their confidence intervals (C.I.) for the three different GBDP formulas:

- formula d_0 (a.k.a. GGDC formula 1): length of all HSPs divided by total genome length
- formula d_4 (a.k.a. GGDC formula 2): sum of all identities found in HSPs divided by overall HSP length
- formula d_6 (a.k.a. GGDC formula 3): sum of all identities found in HSPs divided by total genome length

Note: Formula d_4 is independent of genome length and is thus robust against the use of incomplete draft genomes. For other reasons for preferring formula d_4, see the FAQ.

Query	Subject	d_0	C.I. d_0	d_4	C.I. d_4	d_6	C.I. d_6	Diff. G+C Percent
**'bin.24.fa'**	*Methanothrix concilii* GP6	28.8	[25.4 - 32.4]	26.9	[24.5 - 29.4]	27.2	[24.3 - 30.3]	3.16
**'bin.24.fa'**	*Methanothrix thermophila* PT	12.8	[10.1 - 16.0]	16.1	[14.1 - 18.4]	13.1	[10.8 - 15.9]	0.6
**'bin.24.fa'**	*Isosphaera pallida* ATCC 43644	12.5	[9.8 - 15.7]	3.7	[2.8 - 4.8]	12.9	[10.6 - 15.6]	8.35
**'bin.24.fa'**	*Rickettsia honei* RB	12.5	[9.8 - 15.7]	3.7	[2.8 - 4.8]	12.9	[10.6 - 15.6]	21.73
**'bin.24.fa'**	*Candidatus* Enterenecus merdae ChiHcolR17-2730	12.5	[9.8 - 15.7]	3.7	[2.8 - 4.8]	12.9	[10.6 - 15.6]	9.29
**'bin.24.fa'**	*Faecalibacterium gallinarum* JCM 17207	12.5	[9.8 - 15.7]	3.7	[2.8 - 4.8]	12.9	[10.6 - 15.6]	4.94
**'bin.24.fa'**	*Mycobacterium kiyosense* IWGMT90018-18076	12.5	[9.8 - 15.7]	3.7	[2.8 - 4.8]	12.9	[10.6 - 15.6]	12.94
**'bin.24.fa'**	*Leucobacter soli* CIP 111803	12.5	[9.8 - 15.7]	3.7	[2.8 - 4.8]	12.9	[10.6 - 15.6]	15.36
**'bin.24.fa'**	*Faecalicatena contorta* JCM 6483	12.5	[9.8 - 15.7]	3.7	[2.8 - 4.8]	12.9	[10.6 - 15.6]	8.35
**'bin.24.fa'**	*Rickettsia slovaca* 13-B	12.5	[9.8 - 15.7]	3.7	[2.8 - 4.8]	12.9	[10.6 - 15.6]	21.64

Table 4: Strains in your dataset

Joint dataset of automatically determined closest type strains (if this mode was chosen), manually selected type strains (if selected accordingly) and the provided user strains, if provided (marked in yellow).

Strain	Authority	Other deposits	Synonyms	Base pairs	Percent G+C	No. proteins	Goldstamp	Bioproject accession	Biosample accession	Assembly accession	IMG OID
Rickettsia slovaca 13-B	Sekeyová et al. 1998 emend. Hördt et al. 2020	B	*Rickettsia slovaca*	1275 089	32.5	1114	Gp0001828	PRJNA15712	SAMN02603145	GCA_000237845	2511231061
Candidatus Enterenecus merdae ChiHcolR17-2730	Gilroy et al. 2021		*Candidatus* Enterenecus merdae	1799 706	63.4	1664		PRJNA543206	SAMN15817102	GCA_018716385	
Faecalicatena contorta JCM 6483	(Prévot 1947) Sakamoto et al. 2017	113 VI; ATCC 25540; CCUG 52236; DSM 3982	*Catenabacterium contortum*; *Eubacterium contortum*; *Faecalicatena contorta*	5327 649	45.8	4867		PRJDB10510	SAMD00644941	GCA_039521745	
Rickettsia honei RB	Stenos et al. 1998 emend. Hördt et al. 2020	ATCC VR-1472	*Rickettsia honei*	1268 758	32.4	1469	Gp0021539	PRJNA158665	SAMN02469768	GCA_000263055	2548877054
Isosphaera pallida ATCC 43644	(ex Woronichin 1927) Giovannoni et al. 1995	DSM 9630; IS1B	*Isosphaera pallida*	5529 304	62.5	3722	Gp0001163	PRJNA32825	SAMN00138929	GCA_000186345	649633058
Mycobacterium kiyosense IWGMT90018-18076	Fukano et al. 2023	KCTC 49725; JCM 34837	*Mycobacterium kiyosense*	6346 450	67.1	6263		PRJDB12154	SAMD00407201	GCA_021654635	
Methanothrix concilii GP6	Patel 1985	ATCC 35969; DSM 3671; JCM 10134; NRC 2989; OCM 69	*Methanosaeta concilii*; *Methanothrix concilii*	3026 645	51.0	2850	Gp0002323	PRJNA63185	SAMN02604263	GCA_000204415	650716054
Methanothrix thermophila PT	Kamagata et al. 1992	DSM 6194; JCM 14653; NBRC 101360	*Methanosaeta thermophila*; *Methanothrix thermophila*	1879 471	53.5	1696	Gp0000268	PRJNA15765	SAMN02598350	GCA_000014945	639633038
Faecalibacterium gallinarum JCM 17207	Sakamoto et al. 2022	DSM 23680; ic1379	*Faecalibacterium gallinarum*	2795 642	59.1	2609		PRJDB12731	SAMD00432541	GCA_022180365	

Strain	Authority	Other deposits	Synonyms	Base pairs	Percent G+C	No. proteins	Goldstamp	Bioproject accession	Biosample accession	Assembly accession	IMG OID
Leucobacter soli CIP 111803	Kämpfer et al. 2021	LMG 31600; DSM 110505; CCM 9020; IMT-300	*Leucobacter soli*	2856 782	69.5	2523		PRJEB43055	SAMEA8924568	GCA_910593785	
bin.24.fa				2009 437	54.1	2117					

Methods, Results and References

The genome sequence data were uploaded to the Type (Strain) Genome Server (TYGS), a free bioinformatics platform available under https://tygs.dsmz.de, for a whole genome-based taxonomic analysis [1]. The analysis also made use of recently introduced methodological updates and features [2]. Information on nomenclature, synonymy and associated taxonomic literature was provided by TYGS's sister database, the List of Prokaryotic names with Standing in Nomenclature (LPSN, available at https://lpsn.dsmz.de) [2]. The results were provided by the TYGS on 2024-06-16. The TYGS analysis was subdivided into the following steps:

Determination of closely related type strains

The determination of closely related type strains did not succeed because not a single 16S rDNA gene sequence was detected in the provided user genomes. The subsequent analyses are thus only based on the provided genome data and the manually selected type strains, if any.

Pairwise comparison of genome sequences

For the phylogenomic inference, all pairwise comparisons among the set of genomes were conducted using GBDP and accurate intergenomic distances inferred under the algorithm 'trimming' and distance formula d_5 [3]. 100 distance replicates were calculated each. Digital DDH values and confidence intervals were calculated using the recommended settings of the GGDC 4.0 [2,3].

Phylogenetic inference

The resulting intergenomic distances were used to infer a balanced minimum evolution tree with branch support via FASTME 2.1.6.1 including SPR postprocessing [4]. Branch support was inferred from 100 pseudo-bootstrap replicates each. The trees were rooted at the midpoint [5] and visualized with PhyD3 [6].

Type-based species and subspecies clustering

The type-based species clustering using a 70% dDDH radius around each of the 10 type strains was done as previously described [1]. The resulting groups are shown in Table 1 and 4. Subspecies clustering was done using a 79% dDDH threshold as previously introduced [7].

Results

Type-based species and subspecies clustering

The resulting species and subspecies clusters are listed in Table 4, whereas the taxonomic identification of the query strains is found in Table 1. Briefly, the clustering yielded 10 species clusters and the provided query strains were assigned to 1 of these. Moreover, user strains were located in 1 of 10 subspecies clusters.

Figure caption genome tree

Figure 2. Tree inferred with FastME 2.1.6.1 [4] from GBDP distances calculated from genome sequences. The branch lengths are scaled in terms of GBDP distance formula d_5. The numbers above branches are GBDP pseudo-bootstrap support values > 60 % from 100 replications, with an average branch support of 92.5 %. The tree was rooted at the midpoint [5].

References

[1] Meier-Kolthoff JP, Göker M. TYGS is an automated high-throughput platform for state-of-the-art genome-based taxonomy. Nat. Commun. 2019;10: 2182. DOI: 10.1038/s41467-019-10210-3

[2] Meier-Kolthoff JP, Sardà Carbasse J, Peinado-Olarte RL, Göker M. TYGS and LPSN: a database tandem for fast and reliable genome-based classification and nomenclature of prokaryotes. Nucleic Acid Res. 2022;50: D801–D807. DOI: 10.1093/nar/gkab902

[3] Meier-Kolthoff JP, Auch AF, Klenk H-P, Göker M. Genome sequence-based species delimitation with confidence intervals and improved distance functions. BMC Bioinformatics. 2013;14: 60. DOI: 10.1186/1471-2105-14-60

[4] Lefort V, Desper R, Gascuel O. FastME 2.0: A comprehensive, accurate, and fast distance-based phylogeny inference program. Mol Biol Evol. 2015;32: 2798–2800. DOI: 10.1093/molbev/msv150

[5] Farris JS. Estimating phylogenetic trees from distance matrices. Am Nat. 1972;106: 645–667.

[6] Kreft L, Botzki A, Coppens F, Vandepoele K, Van Bel M. PhyD3: A phylogenetic tree viewer with extended phyloXML support for functional genomics data visualization. Bioinformatics. 2017;33: 2946–2947. DOI: 10.1093/bioinformatics/btx324

[7] Meier-Kolthoff JP, Hahnke RL, Petersen J, Scheuner C, Michael V, Fiebig A, et al. Complete genome sequence of DSM 30083^T, the type strain (U5/41^T) of *Escherichia coli*, and a proposal for delineating subspecies in microbial taxonomy. Stand Genomic Sci. 2014;9: 2. DOI: 10.1186/1944-3277-9-2
