# Supplementary material for: Tracing active members in microbial communities by BONCAT and click chemistry-based enrichment of newly synthesized proteins
Source: ISME Commun. 2024 Dec 4;4(1):ycae153. doi: 10.1093/ismeco/ycae153 (PMC11683836; doi:10.1093/ismeco/ycae153)
Supplement: Genome_Server_ycae153 [file genome_server_ycae153.zip › Genome Server/Bin_11_TYGS_job_results.pdf]

TYPE (STRAIN) GENOME SERVER

JOB SUMMARY

PRINT DATE:	2024-06-17 08:49:49 +0200
JOB ID:	8ce6b427-b617-4191-b16f-6815bae704ea--02
RESULT PAGE:	https://tygs.dsmz.de/user_results/show?guid=8ce6b427-b617-4191-b16f-6815bae704ea--02

Table 1: Phylogenies

Publication-ready versions of both the genome-scale GBDP tree and the 16S rRNA gene sequence tree can be customized and exported either in SVG (vector graphic) or PNG format from within the phylogeny viewers in your TYGS result page. For publications the **SVG format is recommended** because it is lossless, always keeps its high resolution and can also be easily converted to other popular formats such as PDF or EPS. Please follow the link provided above!

Table 2: Identification

The below list contains the result of the TYGS species identification routine.

Explanation of remarks that might occur in the below table:

remark [R1]: The TYGS type strain database is automatically updated on an almost daily basis. However, if a particular type strain genome is not available in the TYGS database, this can have several reasons which are detailed in the FAQ. You can request an extended 16S rRNA gene analysis via the 16S tree viewer found in your result page to detect **not yet genome-sequenced** type strains relevant for your study.

remark [R2]: > 70% dDDH value (formula d_4) and (almost) minimal dDDH values for gene-content formulae d_0 and d_6 indicate a potentially unreliable identification result and should thus be checked via the 16S rRNA gene sequence similarity. Such strong deviations can, in principle, be caused by sequence contamination.

remark [R3]: G+C content difference of > 1 % indicates a potentially unreliable identification result because within species G+C content varies no more than 1 %, if computed from genome sequences (PMID: 24505073).

Strain	Conclusion	Identification result	Remark
**'bin.11'**	potential new species		see [R1]

Table 3: Pairwise comparisons of user genomes vs. type-strain genomes

The following table contains the pairwise dDDH values between your user genomes and the selected type-strain genomes. The dDDH values are provided along with their confidence intervals (C.I.) for the three different GBDP formulas:

- formula d_0 (a.k.a. GGDC formula 1): length of all HSPs divided by total genome length
- formula d_4 (a.k.a. GGDC formula 2): sum of all identities found in HSPs divided by overall HSP length
- formula d_6 (a.k.a. GGDC formula 3): sum of all identities found in HSPs divided by total genome length

Note: Formula d_4 is independent of genome length and is thus robust against the use of incomplete draft genomes. For other reasons for preferring formula d_4, see the FAQ.

Query	Subject	d_0	C.I. d_0	d_4	C.I. d_4	d_6	C.I. d_6	Diff. G+C Percent
**'bin.11.fa'**	*Acetomicrobium hydrogeniformans* ATCC BAA-1850	14.8	[11.9 - 18.2]	18.7	[16.5 - 21.1]	14.9	[12.4 - 17.7]	1.85
**'bin.11.fa'**	*Acetomicrobium thermoterrenum* DSM 13490	14.6	[11.8 - 18.0]	18.6	[16.5 - 21.0]	14.7	[12.3 - 17.6]	1.77
**'bin.11.fa'**	*Acetomicrobium mobile* DSM 13181	14.6	[11.8 - 18.0]	18.6	[16.4 - 20.9]	14.7	[12.3 - 17.6]	3.29
**'bin.11.fa'**	*Acetomicrobium flavidum* DSM 20664	15.1	[12.2 - 18.6]	18.4	[16.3 - 20.8]	15.1	[12.7 - 18.0]	3.61
**'bin.11.fa'**	*Campylobacter canadensis* LMG24001T	12.5	[9.8 - 15.7]	3.7	[2.8 - 4.8]	12.9	[10.6 - 15.6]	17.32
**'bin.11.fa'**	*Acholeplasma modicum* ATCC 29102	12.5	[9.8 - 15.7]	3.7	[2.8 - 4.8]	12.9	[10.6 - 15.6]	16.86
**'bin.11.fa'**	*Thermodesulfobacterium hydrogeniphilum* DSM 14290	12.5	[9.8 - 15.7]	3.7	[2.8 - 4.8]	12.9	[10.6 - 15.6]	14.33
**'bin.11.fa'**	*Vibrio salinus* DNF-1	12.5	[9.8 - 15.7]	3.7	[2.8 - 4.8]	12.9	[10.6 - 15.6]	2.54
**'bin.11.fa'**	*Halocynthiibacter halioticoli* Z654T	12.5	[9.8 - 15.7]	3.7	[2.8 - 4.8]	12.9	[10.6 - 15.6]	9.5
**'bin.11.fa'**	*Thermotoga profunda* AZM34c06	12.5	[9.8 - 15.7]	3.7	[2.8 - 4.8]	12.9	[10.6 - 15.6]	5.88

Table 4: Strains in your dataset

Joint dataset of automatically determined closest type strains (if this mode was chosen), manually selected type strains (if selected accordingly) and the provided user strains, if provided (marked in yellow).

Strain	Authority	Other deposits	Synonyms	Base pairs	Percent G+C	No. proteins	Goldstamp	Bioproject accession	Biosample accession	Assembly accession	IMG OID
Halocynthiibacter halioticoli Z654T	Zhang et al. 2023	MCCC 1H00503; KCTC 92003	*Halocynthiibacter halioticoli*	3296250	54.2	3215		PRJNA224116	SAMN31308952	GCF_025802015	
Vibrio salinus DNF-1	Huang et al. 2023	BCRC 81209; JCM 33626	*Vibrio salinus*	4446507	42.1	3942		PRJNA785371	SAMN23555486	GCA_021272385	
Acholeplasma modicum ATCC 29102	Leach 1973	NCTC 10134; PG 49	*Acholeplasma modicum*; *Haploplasma modicum*	1221086	27.8	1130	Gp0018543	PRJNA223044	SAMN02743893	GCA_000687835	2558860159
Campylobacter canadensis LMG24001T	Inglis et al. 2007	CCUG 54429; DSM 22948; L266	*Campylobacter canadensis*	1923009	27.4	1835		PRJNA284973	SAMN03737966	GCA_013177655	
Thermodesulfobacterium hydrogeniphilum DSM 14290	Jeanthon et al. 2002	JCM 11239; SL6	*Thermodesulfobacterium hydrogeniphilum*	1685468	30.3	1670	Gp0044192	PRJNA239492	SAMN02744824	GCA_000746255	2579779159
Acetomicrobium thermoterrenum DSM 13490	(Rees et al. 1997) Ben Hania et al. 2016	ACM 5076; RWcit; RWcit2	*Acetomicrobium thermoterrenum*; *Anaerobaculum thermoterrenum*	1961877	46.5	1899	Gp0102823	PRJNA262052	SAMN03080603	GCA_900107215	2599185146
Acetomicrobium hydrogeniformans ATCC BAA-1850	(Maune and Tanner 2012) Ben Hania et al. 2016	DSM 22491; OS1	*Acetomicrobium hydrogeniformans*; *Anaerobaculum hydrogeniformans*	2123925	46.5	2197	Gp0025572	PRJNA33123	SAMN00009040	GCA_000160455	
Thermotoga profunda AZM34c06	Mori et al. 2014	DSM 23275; NBRC 106115	*Pseudothermotoga profunda*; *Thermotoga profunda*	2187612	38.8	2101	Gp0091042	PRJDB1509	SAMD00061023	GCA_000828675	
Acetomicrobium mobile DSM 13181	(Menes and Muxí 2002) Ben Hania et al. 2016	ATCC BAA-54; NGA	*Acetomicrobium mobile*; *Anaerobaculum mobile*	2160700	48.0	2017	Gp0004406	PRJNA53351	SAMN02261375	GCA_000266925	2509601011

Strain	Authority	Other deposits	Synonyms	Base pairs	Percent G+C	No. proteins	Goldstamp	Bioproject accession	Biosample accession	Assembly accession	IMG OID
Acetomicrobium flavidum DSM 20664	Soutschek et al. 1985	LMG 6941; ATCC 43122	*Acetomicrobium flavidum*	2021 699	48.3	1944	Gp0148757	PRJNA331522	SAMN05444368	GCA_900129645	2695420966
bin.11.fa				1318 635	44.7	1476					

Methods, Results and References

The genome sequence data were uploaded to the Type (Strain) Genome Server (TYGS), a free bioinformatics platform available under https://tygs.dsmz.de, for a whole genome-based taxonomic analysis [1]. The analysis also made use of recently introduced methodological updates and features [2]. Information on nomenclature, synonymy and associated taxonomic literature was provided by TYGS's sister database, the List of Prokaryotic names with Standing in Nomenclature (LPSN, available at https://lpsn.dsmz.de) [2]. The results were provided by the TYGS on 2024-06-16. The TYGS analysis was subdivided into the following steps:

Determination of closely related type strains

The determination of closely related type strains did not succeed because not a single 16S rDNA gene sequence was detected in the provided user genomes. The subsequent analyses are thus only based on the provided genome data and the manually selected type strains, if any.

Pairwise comparison of genome sequences

For the phylogenomic inference, all pairwise comparisons among the set of genomes were conducted using GBDP and accurate intergenomic distances inferred under the algorithm 'trimming' and distance formula d_5 [3]. 100 distance replicates were calculated each. Digital DDH values and confidence intervals were calculated using the recommended settings of the GGDC 4.0 [2,3].

Phylogenetic inference

The resulting intergenomic distances were used to infer a balanced minimum evolution tree with branch support via FASTME 2.1.6.1 including SPR postprocessing [4]. Branch support was inferred from 100 pseudo-bootstrap replicates each. The trees were rooted at the midpoint [5] and visualized with PhyD3 [6].

Type-based species and subspecies clustering

The type-based species clustering using a 70% dDDH radius around each of the 10 type strains was done as previously described [1]. The resulting groups are shown in Table 1 and 4. Subspecies clustering was done using a 79% dDDH threshold as previously introduced [7].

Results

Type-based species and subspecies clustering

The resulting species and subspecies clusters are listed in Table 4, whereas the taxonomic identification of the query strains is found in Table 1. Briefly, the clustering yielded 9 species clusters and the provided query strains were assigned to 1 of these. Moreover, user strains were located in 1 of 10 subspecies clusters.

Figure caption genome tree

Figure 2. Tree inferred with FastME 2.1.6.1 [4] from GBDP distances calculated from genome sequences. The branch lengths are scaled in terms of GBDP distance formula d_5. The numbers above branches are GBDP pseudo-bootstrap support values > 60 % from 100 replications, with an average branch support of 60.8 %. The tree was rooted at the midpoint [5].

References

[1] Meier-Kolthoff JP, Göker M. TYGS is an automated high-throughput platform for state-of-the-art genome-based taxonomy. Nat. Commun. 2019;10: 2182. DOI: 10.1038/s41467-019-10210-3

[2] Meier-Kolthoff JP, Sardà Carbasse J, Peinado-Olarte RL, Göker M. TYGS and LPSN: a database tandem for fast and reliable genome-based classification and nomenclature of prokaryotes. Nucleic Acid Res. 2022;50: D801–D807. DOI: 10.1093/nar/gkab902

[3] Meier-Kolthoff JP, Auch AF, Klenk H-P, Göker M. Genome sequence-based species delimitation with confidence intervals and improved distance functions. BMC Bioinformatics. 2013;14: 60. DOI: 10.1186/1471-2105-14-60

[4] Lefort V, Desper R, Gascuel O. FastME 2.0: A comprehensive, accurate, and fast distance-based phylogeny inference program. Mol Biol Evol. 2015;32: 2798–2800. DOI: 10.1093/molbev/msv150

[5] Farris JS. Estimating phylogenetic trees from distance matrices. Am Nat. 1972;106: 645–667.

[6] Kreft L, Botzki A, Coppens F, Vandepoele K, Van Bel M. PhyD3: A phylogenetic tree viewer with extended phyloXML support for functional genomics data visualization. Bioinformatics. 2017;33: 2946–2947. DOI: 10.1093/bioinformatics/btx324

[7] Meier-Kolthoff JP, Hahnke RL, Petersen J, Scheuner C, Michael V, Fiebig A, et al. Complete genome sequence of DSM 30083^{T}, the type strain (U5/41^{T}) of *Escherichia coli*, and a proposal for delineating subspecies in microbial taxonomy. Stand Genomic Sci. 2014;9: 2. DOI: 10.1186/1944-3277-9-2
