# Supplementary material for: Tracing active members in microbial communities by BONCAT and click chemistry-based enrichment of newly synthesized proteins
Source: ISME Commun. 2024 Dec 4;4(1):ycae153. doi: 10.1093/ismeco/ycae153 (PMC11683836; doi:10.1093/ismeco/ycae153)
Supplement: Genome_Server_ycae153 [file genome_server_ycae153.zip › Genome Server/Bin_13_TYGS_job_results.pdf]

TYPE (STRAIN) GENOME SERVER

JOB SUMMARY

PRINT DATE:	2024-06-17 08:51:42 +0200
JOB ID:	8ce6b427-b617-4191-b16f-6815bae704ea--03
RESULT PAGE:	https://tygs.dsmz.de/user_results/show?guid=8ce6b427-b617-4191-b16f-6815bae704ea--03

Table 1: Phylogenies

Publication-ready versions of both the genome-scale GBDP tree and the 16S rRNA gene sequence tree can be customized and exported either in SVG (vector graphic) or PNG format from within the phylogeny viewers in your TYGS result page. For publications the **SVG format is recommended** because it is lossless, always keeps its high resolution and can also be easily converted to other popular formats such as PDF or EPS. Please follow the link provided above!

Table 2: Identification

The below list contains the result of the TYGS species identification routine.

Explanation of remarks that might occur in the below table:

remark [R1]: The TYGS type strain database is automatically updated on an almost daily basis. However, if a particular type strain genome is not available in the TYGS database, this can have several reasons which are detailed in the FAQ. You can request an extended 16S rRNA gene analysis via the 16S tree viewer found in your result page to detect **not yet genome-sequenced** type strains relevant for your study.

remark [R2]: > 70% dDDH value (formula d_4) and (almost) minimal dDDH values for gene-content formulae d_0 and d_6 indicate a potentially unreliable identification result and should thus be checked via the 16S rRNA gene sequence similarity. Such strong deviations can, in principle, be caused by sequence contamination.

remark [R3]: G+C content difference of > 1 % indicates a potentially unreliable identification result because within species G+C content varies no more than 1 %, if computed from genome sequences (PMID: 24505073).

Strain	Conclusion	Identification result	Remark
**'bin.13'**	potential new species		see [R1]

Table 3: Pairwise comparisons of user genomes vs. type-strain genomes

The following table contains the pairwise dDDH values between your user genomes and the selected type-strain genomes. The dDDH values are provided along with their confidence intervals (C.I.) for the three different GBDP formulas:

- formula d_0 (a.k.a. GGDC formula 1): length of all HSPs divided by total genome length
- formula d_4 (a.k.a. GGDC formula 2): sum of all identities found in HSPs divided by overall HSP length
- formula d_6 (a.k.a. GGDC formula 3): sum of all identities found in HSPs divided by total genome length

Note: Formula d_4 is independent of genome length and is thus robust against the use of incomplete draft genomes. For other reasons for preferring formula d_4, see the FAQ.

Query	Subject	d_0	C.I. d_0	d_4	C.I. d_4	d_6	C.I. d_6	Diff. G+C Percent
**'bin.13.fa'**	*Streptomyces musisoli* CH5-8T	12.5	[9.8 - 15.7]	68.6	[65.6 - 71.4]	12.9	[10.6 - 15.6]	10.75
**'bin.13.fa'**	*Sphingomonas astaxanthinifaciens* DSM 22298	12.5	[9.8 - 15.7]	41.9	[39.4 - 44.4]	12.9	[10.6 - 15.6]	7.07
**'bin.13.fa'**	*Aminithiophilus ramosus* J.5.4.2-T.3.5.2	12.7	[10.0 - 16.0]	19.6	[17.4 - 22.0]	13.1	[10.8 - 15.8]	2.65
**'bin.13.fa'**	*Dethiosulfovibrio faecalis* F2B	12.5	[9.9 - 15.8]	18.8	[16.7 - 21.2]	12.9	[10.6 - 15.7]	6.49
**'bin.13.fa'**	*Aminobacterium colombiense* DSM 12261	12.5	[9.9 - 15.8]	18.1	[15.9 - 20.4]	12.9	[10.6 - 15.7]	16.03
**'bin.13.fa'**	*Aminomonas paucivorans* DSM 12260	12.8	[10.1 - 16.0]	17.4	[15.3 - 19.8]	13.1	[10.8 - 15.9]	6.25
**'bin.13.fa'**	*Fretibacterium fastidiosum* SGP1	12.5	[9.9 - 15.8]	16.7	[14.6 - 19.0]	12.9	[10.6 - 15.7]	1.66
**'bin.13.fa'**	*Thermovirga lienii* DSM 17291	12.6	[10.0 - 15.9]	16.4	[14.3 - 18.7]	13.0	[10.7 - 15.8]	14.28
**'bin.13.fa'**	*Hydrogenophaga aromaticivorans* D2P1T	12.5	[9.8 - 15.7]	15.0	[13.0 - 17.3]	12.9	[10.6 - 15.6]	4.22
**'bin.13.fa'**	*Candidatus* Desulfovibrio kirbyi ZnDsv-02	12.5	[9.8 - 15.7]	3.7	[2.8 - 4.8]	12.9	[10.6 - 15.6]	7.69

Table 4: Strains in your dataset

Joint dataset of automatically determined closest type strains (if this mode was chosen), manually selected type strains (if selected accordingly) and the provided user strains, if provided (marked in yellow).

Strain	Authority	Other deposits	Synonyms	Base pairs	Percent G+C	No. proteins	Goldstamp	Bioproject accession	Biosample accession	Assembly accession	IMG OID
Fretibacterium fastidiosum SGP1	Vartoukian et al. 2013	DSM 25557; JCM 16858	*Fretibacterium fastidiosum*	2456 860	63.0	1435	Gp0006124	PRJNA45959	SAMEA3138377	GCA_000210715	
Streptomyces musisoli CH5-8T	Duangupama et al. 2021	TBRC 9950; NBRC 113997	*Streptomyces musisoli*	9339 209	72.1	8102		PRJNA224116	SAMN17267016	GCF_016741855	
Thermovirga lienii DSM 17291	Dahle and Birkeland 2006	ATCC BAA-1197; Cas60314	*Thermovirga lienii*	1967 774	47.1	1849	Gp0003689	PRJNA33163	SAMN02232060	GCA_000233775	2505119043
Sphingomonas astaxanthinifaciens DSM 22298	Asker et al. 2008	CCUG 53608; NBRC 102146; TDMA-17	*Sphingomonas astaxanthinifaciens*	2533 034	68.4	2511	Gp0013294	PRJNA234792	SAMN02691714	GCA_000711715	2565957137
Aminobacterium colombiense DSM 12261	Baena et al. 1999	ALA-1	*Aminobacterium colombiense*	1980 592	45.3	1876	Gp0003444	PRJNA32587	SAMN02598488	GCA_000025885	646564503
Aminomonas paucivorans DSM 12260	Baena et al. 1999	ATCC BAA-6; GLU-3	*Aminomonas paucivorans*	2630 070	67.6	2399	Gp0002272	PRJNA33371	SAMN02256532	GCA_000165795	2502790015
Hydrogenophaga aromaticivorans D2P1T	Banerjee et al. 2021	LMG 31780; NCAIM B.02655	*Hydrogenophaga aromaticivorans*	5628 204	65.6	5274		PRJNA224116	SAMN12757153	GCF_013387465	
Candidatus Desulfovibrio kirbyi ZnDsv-02	Takeuchi et al. 2020		*Candidatus* Desulfovibrio kirbyi	1600 916	53.6	1257		PRJDB9170	SAMD00200503	GCA_013374015	
Dethiosulfovibrio faecalis F2B	Grabowski et al. 2022	KCTC 25378; DSM 112078	*Dethiosulfovibrio faecalis*	2663 927	54.8	2523		PRJNA613861	SAMN14421011	GCA_021568795	
Aminithiophilus ramosus J.5.4.2-T.3.5.2	Pradel et al. 2023	DSM 107166; NBRC 114655	*Aminithiophilus ramosus*	3108 337	64.0	2678		PRJNA720826	SAMN18680547	GCA_018069705	

Strain	Authority	Other deposits	Synonyms	Base pairs	Percent G+C	No. proteins	Goldstamp	Bioproject accession	Biosample accession	Assembly accession	IMG OID
bin.13.fa				1492 635	61.3	1549					

Methods, Results and References

The genome sequence data were uploaded to the Type (Strain) Genome Server (TYGS), a free bioinformatics platform available under https://tygs.dsmz.de, for a whole genome-based taxonomic analysis [1]. The analysis also made use of recently introduced methodological updates and features [2]. Information on nomenclature, synonymy and associated taxonomic literature was provided by TYGS's sister database, the List of Prokaryotic names with Standing in Nomenclature (LPSN, available at https://lpsn.dsmz.de) [2]. The results were provided by the TYGS on 2024-06-16. The TYGS analysis was subdivided into the following steps:

Determination of closely related type strains

The determination of closely related type strains did not succeed because not a single 16S rDNA gene sequence was detected in the provided user genomes. The subsequent analyses are thus only based on the provided genome data and the manually selected type strains, if any.

Pairwise comparison of genome sequences

For the phylogenomic inference, all pairwise comparisons among the set of genomes were conducted using GBDP and accurate intergenomic distances inferred under the algorithm 'trimming' and distance formula d_5 [3]. 100 distance replicates were calculated each. Digital DDH values and confidence intervals were calculated using the recommended settings of the GGDC 4.0 [2,3].

Phylogenetic inference

The resulting intergenomic distances were used to infer a balanced minimum evolution tree with branch support via FASTME 2.1.6.1 including SPR postprocessing [4]. Branch support was inferred from 100 pseudo-bootstrap replicates each. The trees were rooted at the midpoint [5] and visualized with PhyD3 [6].

Type-based species and subspecies clustering

The type-based species clustering using a 70% dDDH radius around each of the 10 type strains was done as previously described [1]. The resulting groups are shown in Table 1 and 4. Subspecies clustering was done using a 79% dDDH threshold as previously introduced [7].

Results

Type-based species and subspecies clustering

The resulting species and subspecies clusters are listed in Table 4, whereas the taxonomic identification of the query strains is found in Table 1. Briefly, the clustering yielded 11 species clusters and the provided query strains were assigned to 1 of these. Moreover, user strains were located in 1 of 11 subspecies clusters.

Figure caption genome tree

Figure 2. Tree inferred with FastME 2.1.6.1 [4] from GBDP distances calculated from genome sequences. The branch lengths are scaled in terms of GBDP distance formula d_5. The numbers above branches are GBDP pseudo-bootstrap support values > 60 % from 100 replications, with an average branch support of 46.6 %. The tree was rooted at the midpoint [5].

References

[1] Meier-Kolthoff JP, Göker M. TYGS is an automated high-throughput platform for state-of-the-art genome-based taxonomy. Nat. Commun. 2019;10: 2182. DOI: 10.1038/s41467-019-10210-3

[2] Meier-Kolthoff JP, Sardà Carbasse J, Peinado-Olarte RL, Göker M. TYGS and LPSN: a database tandem for fast and reliable genome-based classification and nomenclature of prokaryotes. Nucleic Acid Res. 2022;50: D801–D807. DOI: 10.1093/nar/gkab902

[3] Meier-Kolthoff JP, Auch AF, Klenk H-P, Göker M. Genome sequence-based species delimitation with confidence intervals and improved distance functions. BMC Bioinformatics. 2013;14: 60. DOI: 10.1186/1471-2105-14-60

[4] Lefort V, Desper R, Gascuel O. FastME 2.0: A comprehensive, accurate, and fast distance-based phylogeny inference program. Mol Biol Evol. 2015;32: 2798–2800. DOI: 10.1093/molbev/msv150

[5] Farris JS. Estimating phylogenetic trees from distance matrices. Am Nat. 1972;106: 645–667.

[6] Kreft L, Botzki A, Coppens F, Vandepoele K, Van Bel M. PhyD3: A phylogenetic tree viewer with extended phyloXML support for functional genomics data visualization. Bioinformatics. 2017;33: 2946–2947. DOI: 10.1093/bioinformatics/btx324

[7] Meier-Kolthoff JP, Hahnke RL, Petersen J, Scheuner C, Michael V, Fiebig A, et al. Complete genome sequence of DSM 30083^{T}, the type strain (U5/41^{T}) of *Escherichia coli*, and a proposal for delineating subspecies in microbial taxonomy. Stand Genomic Sci. 2014;9: 2. DOI: 10.1186/1944-3277-9-2
