# Supplementary material for: Tracing active members in microbial communities by BONCAT and click chemistry-based enrichment of newly synthesized proteins
Source: ISME Commun. 2024 Dec 4;4(1):ycae153. doi: 10.1093/ismeco/ycae153 (PMC11683836; doi:10.1093/ismeco/ycae153)
Supplement: Genome_Server_ycae153 [file genome_server_ycae153.zip › Genome Server/Bin_16_TYGS_job_results.pdf]

TYPE (STRAIN) GENOME SERVER

JOB SUMMARY

PRINT DATE:	2024-06-17 08:53:01 +0200
JOB ID:	8ce6b427-b617-4191-b16f-6815bae704ea--04
RESULT PAGE:	https://tygs.dsmz.de/user_results/show?guid=8ce6b427-b617-4191-b16f-6815bae704ea--04

Table 1: Phylogenies

Publication-ready versions of both the genome-scale GBDP tree and the 16S rRNA gene sequence tree can be customized and exported either in SVG (vector graphic) or PNG format from within the phylogeny viewers in your TYGS result page. For publications the **SVG format is recommended** because it is lossless, always keeps its high resolution and can also be easily converted to other popular formats such as PDF or EPS. Please follow the link provided above!

Table 2: Identification

The below list contains the result of the TYGS species identification routine.

Explanation of remarks that might occur in the below table:

remark [R1]: The TYGS type strain database is automatically updated on an almost daily basis. However, if a particular type strain genome is not available in the TYGS database, this can have several reasons which are detailed in the FAQ. You can request an extended 16S rRNA gene analysis via the 16S tree viewer found in your result page to detect **not yet genome-sequenced** type strains relevant for your study.

remark [R2]: > 70% dDDH value (formula d_4) and (almost) minimal dDDH values for gene-content formulae d_0 and d_6 indicate a potentially unreliable identification result and should thus be checked via the 16S rRNA gene sequence similarity. Such strong deviations can, in principle, be caused by sequence contamination.

remark [R3]: G+C content difference of > 1 % indicates a potentially unreliable identification result because within species G+C content varies no more than 1 %, if computed from genome sequences (PMID: 24505073).

Strain	Conclusion	Identification result	Remark
**'bin.16'**	potential new species		see [R1]

Table 3: Pairwise comparisons of user genomes vs. type-strain genomes

The following table contains the pairwise dDDH values between your user genomes and the selected type-strain genomes. The dDDH values are provided along with their confidence intervals (C.I.) for the three different GBDP formulas:

- formula d_0 (a.k.a. GGDC formula 1): length of all HSPs divided by total genome length
- formula d_4 (a.k.a. GGDC formula 2): sum of all identities found in HSPs divided by overall HSP length
- formula d_6 (a.k.a. GGDC formula 3): sum of all identities found in HSPs divided by total genome length

Note: Formula d_4 is independent of genome length and is thus robust against the use of incomplete draft genomes. For other reasons for preferring formula d_4, see the FAQ.

Query	Subject	d_0	C.I. d_0	d_4	C.I. d_4	d_6	C.I. d_6	Diff. G+C Percent
**'bin.16.fa'**	*Sphaerochaeta globosa* Buddy	13.0	[10.3 - 16.3]	16.8	[14.7 - 19.1]	13.3	[11.0 - 16.1]	4.83
**'bin.16.fa'**	*Sphaerochaeta associata* GLS2	13.0	[10.3 - 16.3]	16.8	[14.7 - 19.1]	13.4	[11.0 - 16.1]	3.12
**'bin.16.fa'**	*Candidatus* Aphodenecus pullistercoris 11167	12.8	[10.1 - 16.0]	15.8	[13.7 - 18.1]	13.1	[10.8 - 15.9]	5.32
**'bin.16.fa'**	*Leptotrichia buccalis* DSM 1135	12.5	[9.8 - 15.7]	3.7	[2.8 - 4.8]	12.9	[10.6 - 15.6]	24.06
**'bin.16.fa'**	*Leptotrichia shahii* DSM 19757	12.5	[9.8 - 15.7]	3.7	[2.8 - 4.8]	12.9	[10.6 - 15.6]	24.15
**'bin.16.fa'**	*Pseudoxanthomonas broegbernensis* DSM 12573	12.5	[9.8 - 15.7]	3.7	[2.8 - 4.8]	12.9	[10.6 - 15.6]	16.97
**'bin.16.fa'**	*Sneathia sanguinegens* CCUG 41628	12.5	[9.8 - 15.7]	3.7	[2.8 - 4.8]	12.9	[10.6 - 15.6]	26.96
**'bin.16.fa'**	*Brachybacterium timonense* Marseille-P4339	12.5	[9.8 - 15.7]	3.7	[2.8 - 4.8]	12.9	[10.6 - 15.6]	13.58
**'bin.16.fa'**	*Candidatus* Mesorhizobium australafricanum VK9D	12.5	[9.8 - 15.7]	3.7	[2.8 - 4.8]	12.9	[10.6 - 15.6]	9.45
**'bin.16.fa'**	*Pseudomonas boreopolis* JCM 13306	12.5	[9.8 - 15.7]	3.7	[2.8 - 4.8]	12.9	[10.6 - 15.6]	15.27

Table 4: Strains in your dataset

Joint dataset of automatically determined closest type strains (if this mode was chosen), manually selected type strains (if selected accordingly) and the provided user strains, if provided (marked in yellow).

Strain	Authority	Other deposits	Synonyms	Base pairs	Percent G+C	No. proteins	Goldstamp	Bioproject accession	Biosample accession	Assembly accession	IMG OID
Brachybacterium timonense Marseille-P4339	Kuete et al. 2019	CSUR P4339	*Brachybacterium timonense*	3091 410	67.3	2684		PRJEB23296	SAMEA104557109	GCA_900258505	
Leptotrichia shahii DSM 19757	Eribe et al. 2004	CIP 107916; CCUG 47503; JCM 16776; LB 37; VPI N06A-34	*Leptotrichia shahii*	2148 931	29.6	1968	Gp0013904	PRJNA169703	SAMN02440425	GCA_000373045	2515154071
Candidatus Aphodenecus pullistercoris 11167	Gilroy et al. 2021		*Candidatus* Aphodenecus pullistercoris	2073 588	59.0	1946		PRJNA543206	SAMN15817123	GCA_017694495	
Sphaerochaeta associata GLS2	Troshina et al. 2015	DSM 26261; VKM B-2742	*Sphaerochaeta associata*	3545 847	50.6	3251	Gp0157006	PRJEB20704	SAMN06298221	GCA_900182845	
Pseudoxanthomonas broegbernensis DSM 12573	Finkmann et al. 2000 emend. Thierry et al. 2004	CIP 107227; ATCC BAA-10; CCUG 46890; NBRC 101013; B1616/1	*Pseudoxanthomonas broegbernensis*	3519 948	70.7	2996		PRJNA376262	SAMN06368556	GCA_010093165	
Candidatus Mesorhizobium australafricanum VK9D	van Lill et al. 2024	VK9D Ts	*Candidatus* Mesorhizobium australafricanum	6451 413	63.2	6104		PRJNA224116	SAMN37150256	GCF_033977265	
Pseudomonas boreopolis JCM 13306	Gray and Thornton 1928	LMG 979; CIP 106717; NCIMB 9401; ATCC 33662; CCUG 3365; DSM 19620; NCIB 9401	*Pseudomonas boreopolis*	4323 998	69.0	3833		PRJDB10510	SAMD00245232	GCA_014654535	

Strain	Authority	Other deposits	Synonyms	Base pairs	Percent G+C	No. proteins	Goldstamp	Bioproject accession	Biosample accession	Assembly accession	IMG OID
Leptotrichia buccalis DSM 1135	(Robin 1853) Trevisan 1879	CIP 105792; ATCC 14201; CCUG 34316; JCM 12969; NCTC 10249; NCTC 14386	*Leptothrix buccalis*; *Leptotrichia buccalis*	2465 610	29.6	2220	Gp0001215	PRJNA29445	SAMN00002593	GCA_000023905	644736384
Sneathia sanguinegens CCUG 41628	Collins et al. 2002	CIP 106906; DSM 22970	*Sneathia sanguinegens*	1291 023	26.7	1172	Gp0149659	PRJNA224116	SAMN04320708	GCF_001517935	
Sphaerochaeta globosa Buddy	Ritalahti et al. 2012 emend. Hördt et al. 2020	ATCC BAA-1886; DSM 22777	*Sphaerochaeta globosa*	3316 466	48.9	3017	Gp0004903	PRJNA47953	SAMN00713587	GCA_000190435	650377973
bin.16.fa				1529 410	53.7	1509					

Methods, Results and References

The genome sequence data were uploaded to the Type (Strain) Genome Server (TYGS), a free bioinformatics platform available under https://tygs.dsmz.de, for a whole genome-based taxonomic analysis [1]. The analysis also made use of recently introduced methodological updates and features [2]. Information on nomenclature, synonymy and associated taxonomic literature was provided by TYGS's sister database, the List of Prokaryotic names with Standing in Nomenclature (LPSN, available at https://lpsn.dsmz.de) [2]. The results were provided by the TYGS on 2024-06-16. The TYGS analysis was subdivided into the following steps:

Determination of closely related type strains

The determination of closely related type strains did not succeed because not a single 16S rDNA gene sequence was detected in the provided user genomes. The subsequent analyses are thus only based on the provided genome data and the manually selected type strains, if any.

Pairwise comparison of genome sequences

For the phylogenomic inference, all pairwise comparisons among the set of genomes were conducted using GBDP and accurate intergenomic distances inferred under the algorithm 'trimming' and distance formula d_5 [3]. 100 distance replicates were calculated each. Digital DDH values and confidence intervals were calculated using the recommended settings of the GGDC 4.0 [2,3].

Phylogenetic inference

The resulting intergenomic distances were used to infer a balanced minimum evolution tree with branch support via FASTME 2.1.6.1 including SPR postprocessing [4]. Branch support was inferred from 100 pseudo-bootstrap replicates each. The trees were rooted at the midpoint [5] and visualized with PhyD3 [6].

Type-based species and subspecies clustering

The type-based species clustering using a 70% dDDH radius around each of the 10 type strains was done as previously described [1]. The resulting groups are shown in Table 1 and 4. Subspecies clustering was done using a 79% dDDH threshold as previously introduced [7].

Results

Type-based species and subspecies clustering

The resulting species and subspecies clusters are listed in Table 4, whereas the taxonomic identification of the query strains is found in Table 1. Briefly, the clustering yielded 11 species clusters and the provided query strains were assigned to 1 of these. Moreover, user strains were located in 1 of 11 subspecies clusters.

Figure caption genome tree

Figure 2. Tree inferred with FastME 2.1.6.1 [4] from GBDP distances calculated from genome sequences. The branch lengths are scaled in terms of GBDP distance formula d_5. The numbers above branches are GBDP pseudo-bootstrap support values > 60 % from 100 replications, with an average branch support of 86.1 %. The tree was rooted at the midpoint [5].

References

[1] Meier-Kolthoff JP, Göker M. TYGS is an automated high-throughput platform for state-of-the-art genome-based taxonomy. Nat. Commun. 2019;10: 2182. DOI: 10.1038/s41467-019-10210-3

[2] Meier-Kolthoff JP, Sardà Carbasse J, Peinado-Olarte RL, Göker M. TYGS and LPSN: a database tandem for fast and reliable genome-based classification and nomenclature of prokaryotes. Nucleic Acid Res. 2022;50: D801–D807. DOI: 10.1093/nar/gkab902

[3] Meier-Kolthoff JP, Auch AF, Klenk H-P, Göker M. Genome sequence-based species delimitation with confidence intervals and improved distance functions. BMC Bioinformatics. 2013;14: 60. DOI: 10.1186/1471-2105-14-60

[4] Lefort V, Desper R, Gascuel O. FastME 2.0: A comprehensive, accurate, and fast distance-based phylogeny inference program. Mol Biol Evol. 2015;32: 2798–2800. DOI: 10.1093/molbev/msv150

[5] Farris JS. Estimating phylogenetic trees from distance matrices. Am Nat. 1972;106: 645–667.

[6] Kreft L, Botzki A, Coppens F, Vandepoele K, Van Bel M. PhyD3: A phylogenetic tree viewer with extended phyloXML support for functional genomics data visualization. Bioinformatics. 2017;33: 2946–2947. DOI: 10.1093/bioinformatics/btx324

[7] Meier-Kolthoff JP, Hahnke RL, Petersen J, Scheuner C, Michael V, Fiebig A, et al. Complete genome sequence of DSM 30083^{T}, the type strain (U5/41^{T}) of *Escherichia coli*, and a proposal for delineating subspecies in microbial taxonomy. Stand Genomic Sci. 2014;9: 2. DOI: 10.1186/1944-3277-9-2
