# Supplementary material for: Tracing active members in microbial communities by BONCAT and click chemistry-based enrichment of newly synthesized proteins
Source: ISME Commun. 2024 Dec 4;4(1):ycae153. doi: 10.1093/ismeco/ycae153 (PMC11683836; doi:10.1093/ismeco/ycae153)
Supplement: Genome_Server_ycae153 [file genome_server_ycae153.zip › Genome Server/Bin_19_TYGS_job_results.pdf]

TYPE (STRAIN) GENOME SERVER

JOB SUMMARY

PRINT DATE:	2024-06-17 08:55:00 +0200
JOB ID:	8ce6b427-b617-4191-b16f-6815bae704ea--05
RESULT PAGE:	https://tygs.dsmz.de/user_results/show?guid=8ce6b427-b617-4191-b16f-6815bae704ea--05

Table 1: Phylogenies

Publication-ready versions of both the genome-scale GBDP tree and the 16S rRNA gene sequence tree can be customized and exported either in SVG (vector graphic) or PNG format from within the phylogeny viewers in your TYGS result page. For publications the **SVG format is recommended** because it is lossless, always keeps its high resolution and can also be easily converted to other popular formats such as PDF or EPS. Please follow the link provided above!

Table 2: Identification

The below list contains the result of the TYGS species identification routine.

Explanation of remarks that might occur in the below table:

remark [R1]: The TYGS type strain database is automatically updated on an almost daily basis. However, if a particular type strain genome is not available in the TYGS database, this can have several reasons which are detailed in the FAQ. You can request an extended 16S rRNA gene analysis via the 16S tree viewer found in your result page to detect **not yet genome-sequenced** type strains relevant for your study.

remark [R2]: > 70% dDDH value (formula d_4) and (almost) minimal dDDH values for gene-content formulae d_0 and d_6 indicate a potentially unreliable identification result and should thus be checked via the 16S rRNA gene sequence similarity. Such strong deviations can, in principle, be caused by sequence contamination.

remark [R3]: G+C content difference of > 1 % indicates a potentially unreliable identification result because within species G+C content varies no more than 1 %, if computed from genome sequences (PMID: 24505073).

Strain	Conclusion	Identification result	Remark
**'bin.19'**	potential new species		see [R1]

Table 3: Pairwise comparisons of user genomes vs. type-strain genomes

The following table contains the pairwise dDDH values between your user genomes and the selected type-strain genomes. The dDDH values are provided along with their confidence intervals (C.I.) for the three different GBDP formulas:

- formula d_0 (a.k.a. GGDC formula 1): length of all HSPs divided by total genome length
- formula d_4 (a.k.a. GGDC formula 2): sum of all identities found in HSPs divided by overall HSP length
- formula d_6 (a.k.a. GGDC formula 3): sum of all identities found in HSPs divided by total genome length

Note: Formula d_4 is independent of genome length and is thus robust against the use of incomplete draft genomes. For other reasons for preferring formula d_4, see the FAQ.

Query	Subject	d_0	C.I. d_0	d_4	C.I. d_4	d_6	C.I. d_6	Diff. G+C Percent
**'bin.19.fa'**	*Methanobrevibacter olleyae* DSM 16632	12.5	[9.8 - 15.7]	42.8	[40.3 - 45.3]	12.9	[10.6 - 15.6]	14.16
**'bin.19.fa'**	*Methanobacterium subterraneum* DSM 11074	12.6	[9.9 - 15.9]	23.5	[21.2 - 26.0]	13.0	[10.7 - 15.8]	1.56
**'bin.19.fa'**	*Methanobacterium petrolearium* DSM 22353	12.7	[10.0 - 16.0]	19.2	[17.0 - 21.6]	13.1	[10.8 - 15.8]	3.2
**'bin.19.fa'**	*Neobittarella massiliensis* Marseille-P4047	12.5	[9.8 - 15.7]	3.7	[2.8 - 4.8]	12.9	[10.6 - 15.6]	17.6
**'bin.19.fa'**	*Cellulophaga algicola* DSM 14237	12.5	[9.8 - 15.7]	3.7	[2.8 - 4.8]	12.9	[10.6 - 15.6]	7.2
**'bin.19.fa'**	*Enterococcus ratti* DSM 15687	12.5	[9.8 - 15.7]	3.7	[2.8 - 4.8]	12.9	[10.6 - 15.6]	6.67
**'bin.19.fa'**	*Labilibacter sediminis* CG51	12.5	[9.8 - 15.7]	3.7	[2.8 - 4.8]	12.9	[10.6 - 15.6]	5.13
**'bin.19.fa'**	*Actinomyces israelii* DSM 43320	12.5	[9.8 - 15.7]	3.7	[2.8 - 4.8]	12.9	[10.6 - 15.6]	30.47
**'bin.19.fa'**	*Flavobacterium covae* AL-02-36T	12.5	[9.8 - 15.7]	3.7	[2.8 - 4.8]	12.9	[10.6 - 15.6]	10.01
**'bin.19.fa'**	*Methanimicrococcus hongohii* Hf6T	12.5	[9.8 - 15.7]	3.7	[2.8 - 4.8]	12.9	[10.6 - 15.6]	0.22

Table 4: Strains in your dataset

Joint dataset of automatically determined closest type strains (if this mode was chosen), manually selected type strains (if selected accordingly) and the provided user strains, if provided (marked in yellow).

Strain	Authority	Other deposits	Synonyms	Base pairs	Percent G+C	No. proteins	Goldstamp	Bioproject accession	Biosample accession	Assembly accession	IMG OID
Enterococcus ratti DSM 15687	Teixeira et al. 2001	CIP 107173; NCIMB 13635; ATCC 700914; CCUG 43228; NBRC 100698; DS 2705-87	*Enterococcus ratti*	2485 649	34.3	2411		PRJNA270385	SAMN03267184	GCA_001886195	
Methanobacterium petrolearium DSM 22353	Mori and Harayama 2011	NBRC 105198; Mic5c12	*Methanobacterium petrolearium*	2468 550	37.8	2406	Gp0502673				2913397459
Methanimicrococcus hongohii Hf6T	Protasov and Brune 2023	DSM 114388; JCM 39385	*Methanimicrococcus hongohii*	2193 213	41.2	1764		PRJNA983582	SAMN34026937	GCA_032594095	
Methanobrevibacter olleyae DSM 16632	Rea et al. 2007	KM1H5-1P; OCM 841	*Methanobrevibacter olleyae*	2122 096	26.8	1813	Gp0087972	PRJEB17460	SAMN02910297	GCA_900114585	
Labilibacter sediminis CG51	Wang et al. 2020	MCCC 1K03739; JCM 33138	*Labilibacter sediminis*	6222 519	35.8	4602		PRJNA533522	SAMN11458608	GCA_005877885	
Methanobacterium subterraneum DSM 11074	Kotelnikova et al. 1998	A8p; ATCC 700657	*Methanobacterium subterraneum*	2515 817	39.4	2326	Gp0300986	PRJNA224116	SAMN05928842	GCF_002813695	
Actinomyces israelii DSM 43320	(Kruse 1896) Lachner-Sandoval 1898 emend. Nouioui et al. 2018	CIP 103259; ATCC 12102; CCUG 18307; JCM 12964; NCTC 12972	*Actinobacterium israeli; Actinomyces israelii; Streptothrix israeli*	4025 833	71.4	3346	Gp0013936	PRJNA215659	SAMN02745698	GCA_000711965	2565956542
Cellulophaga algicola DSM 14237	Bowman 2000 emend. Hahnke et al. 2016	ACAM 630; IC166	*Cellulophaga algicola*	4888 353	33.8	4163	Gp0004500	PRJNA41529	SAMN00190054	GCA_000186265	649633031

Strain	Authority	Other deposits	Synonyms	Base pairs	Percent G+C	No. proteins	Goldstamp	Bioproject accession	Biosample accession	Assembly accession	IMG OID
Neobittarella massiliensis Marseille-P4047	Bilen et al. 2018	CSUR P4047	*Neobittarella massiliensis*	3228298	58.6	2897		PRJEB22826	SAMEA104572235	GCA_900289145	
Flavobacterium covae AL-02-36T	La Frentz et al. 2022	ATCC TSD-246; CCM 9171	*Flavobacterium covae*	3401157	31.0	2356		PRJNA684153	SAMN17052638	GCA_019565575	
bin.19.fa				529197	41.0	633					

Methods, Results and References

The genome sequence data were uploaded to the Type (Strain) Genome Server (TYGS), a free bioinformatics platform available under https://tygs.dsmz.de, for a whole genome-based taxonomic analysis [1]. The analysis also made use of recently introduced methodological updates and features [2]. Information on nomenclature, synonymy and associated taxonomic literature was provided by TYGS's sister database, the List of Prokaryotic names with Standing in Nomenclature (LPSN, available at https://lpsn.dsmz.de) [2]. The results were provided by the TYGS on 2024-06-16. The TYGS analysis was subdivided into the following steps:

Determination of closely related type strains

The determination of closely related type strains did not succeed because not a single 16S rDNA gene sequence was detected in the provided user genomes. The subsequent analyses are thus only based on the provided genome data and the manually selected type strains, if any.

Pairwise comparison of genome sequences

For the phylogenomic inference, all pairwise comparisons among the set of genomes were conducted using GBDP and accurate intergenomic distances inferred under the algorithm 'trimming' and distance formula d_5 [3]. 100 distance replicates were calculated each. Digital DDH values and confidence intervals were calculated using the recommended settings of the GGDC 4.0 [2,3].

Phylogenetic inference

The resulting intergenomic distances were used to infer a balanced minimum evolution tree with branch support via FASTME 2.1.6.1 including SPR postprocessing [4]. Branch support was inferred from 100 pseudo-bootstrap replicates each. The trees were rooted at the midpoint [5] and visualized with PhyD3 [6].

Type-based species and subspecies clustering

The type-based species clustering using a 70% dDDH radius around each of the 10 type strains was done as previously described [1]. The resulting groups are shown in Table 1 and 4. Subspecies clustering was done using a 79% dDDH threshold as previously introduced [7].

Results

Type-based species and subspecies clustering

The resulting species and subspecies clusters are listed in Table 4, whereas the taxonomic identification of the query strains is found in Table 1. Briefly, the clustering yielded 11 species clusters and the provided query strains were assigned to 1 of these. Moreover, user strains were located in 1 of 11 subspecies clusters.

Figure caption genome tree

Figure 2. Tree inferred with FastME 2.1.6.1 [4] from GBDP distances calculated from genome sequences. The branch lengths are scaled in terms of GBDP distance formula d_5. The numbers above branches are GBDP pseudo-bootstrap support values > 60 % from 100 replications, with an average branch support of 83.4 %. The tree was rooted at the midpoint [5].

References

[1] Meier-Kolthoff JP, Göker M. TYGS is an automated high-throughput platform for state-of-the-art genome-based taxonomy. Nat. Commun. 2019;10: 2182. DOI: 10.1038/s41467-019-10210-3

[2] Meier-Kolthoff JP, Sardà Carbasse J, Peinado-Olarte RL, Göker M. TYGS and LPSN: a database tandem for fast and reliable genome-based classification and nomenclature of prokaryotes. Nucleic Acid Res. 2022;50: D801–D807. DOI: 10.1093/nar/gkab902

[3] Meier-Kolthoff JP, Auch AF, Klenk H-P, Göker M. Genome sequence-based species delimitation with confidence intervals and improved distance functions. BMC Bioinformatics. 2013;14: 60. DOI: 10.1186/1471-2105-14-60

[4] Lefort V, Desper R, Gascuel O. FastME 2.0: A comprehensive, accurate, and fast distance-based phylogeny inference program. Mol Biol Evol. 2015;32: 2798–2800. DOI: 10.1093/molbev/msv150

[5] Farris JS. Estimating phylogenetic trees from distance matrices. Am Nat. 1972;106: 645–667.

[6] Kreft L, Botzki A, Coppens F, Vandepoele K, Van Bel M. PhyD3: A phylogenetic tree viewer with extended phyloXML support for functional genomics data visualization. Bioinformatics. 2017;33: 2946–2947. DOI: 10.1093/bioinformatics/btx324

[7] Meier-Kolthoff JP, Hahnke RL, Petersen J, Scheuner C, Michael V, Fiebig A, et al. Complete genome sequence of DSM 30083^{T}, the type strain (U5/41^{T}) of *Escherichia coli*, and a proposal for delineating subspecies in microbial taxonomy. Stand Genomic Sci. 2014;9: 2. DOI: 10.1186/1944-3277-9-2
