# Supplementary material for: Tracing active members in microbial communities by BONCAT and click chemistry-based enrichment of newly synthesized proteins
Source: ISME Commun. 2024 Dec 4;4(1):ycae153. doi: 10.1093/ismeco/ycae153 (PMC11683836; doi:10.1093/ismeco/ycae153)
Supplement: Genome_Server_ycae153 [file genome_server_ycae153.zip › Genome Server/Bin_20_TYGS_job_results.pdf]

TYPE (STRAIN) GENOME SERVER

JOB SUMMARY

PRINT DATE:	2024-06-17 08:57:39 +0200
JOB ID:	8ce6b427-b617-4191-b16f-6815bae704ea--06
RESULT PAGE:	https://tygs.dsmz.de/user_results/show?guid=8ce6b427-b617-4191-b16f-6815bae704ea--06

Table 1: Phylogenies

Publication-ready versions of both the genome-scale GBDP tree and the 16S rRNA gene sequence tree can be customized and exported either in SVG (vector graphic) or PNG format from within the phylogeny viewers in your TYGS result page. For publications the **SVG format is recommended** because it is lossless, always keeps its high resolution and can also be easily converted to other popular formats such as PDF or EPS. Please follow the link provided above!

Table 2: Identification

The below list contains the result of the TYGS species identification routine.

Explanation of remarks that might occur in the below table:

remark [R1]: The TYGS type strain database is automatically updated on an almost daily basis. However, if a particular type strain genome is not available in the TYGS database, this can have several reasons which are detailed in the FAQ. You can request an extended 16S rRNA gene analysis via the 16S tree viewer found in your result page to detect **not yet genome-sequenced** type strains relevant for your study.

remark [R2]: > 70% dDDH value (formula d_4) and (almost) minimal dDDH values for gene-content formulae d_0 and d_6 indicate a potentially unreliable identification result and should thus be checked via the 16S rRNA gene sequence similarity. Such strong deviations can, in principle, be caused by sequence contamination.

remark [R3]: G+C content difference of > 1 % indicates a potentially unreliable identification result because within species G+C content varies no more than 1 %, if computed from genome sequences (PMID: 24505073).

Strain	Conclusion	Identification result	Remark
**'bin.20'**	potential new species		see [R1]

Table 3: Pairwise comparisons of user genomes vs. type-strain genomes

The following table contains the pairwise dDDH values between your user genomes and the selected type-strain genomes. The dDDH values are provided along with their confidence intervals (C.I.) for the three different GBDP formulas:

- formula d_0 (a.k.a. GGDC formula 1): length of all HSPs divided by total genome length
- formula d_4 (a.k.a. GGDC formula 2): sum of all identities found in HSPs divided by overall HSP length
- formula d_6 (a.k.a. GGDC formula 3): sum of all identities found in HSPs divided by total genome length

Note: Formula d_4 is independent of genome length and is thus robust against the use of incomplete draft genomes. For other reasons for preferring formula d_4, see the FAQ.

Query	Subject	d_0	C.I. d_0	d_4	C.I. d_4	d_6	C.I. d_6	Diff. G+C Percent
**'bin.20.fa'**	*Castellaniella defragrans* CCUG 39790	35.7	[32.3 - 39.2]	32.6	[30.2 - 35.1]	34.0	[31.1 - 37.1]	4.58
**'bin.20.fa'**	*Castellaniella defragrans* DSM 12141	37.1	[33.7 - 40.6]	32.1	[29.7 - 34.6]	35.0	[32.1 - 38.1]	4.63
**'bin.20.fa'**	*Castellaniella daejeonensis* JCM16240	50.9	[47.4 - 54.3]	30.0	[27.7 - 32.6]	44.8	[41.8 - 47.9]	1.98
**'bin.20.fa'**	*Castellaniella ginsengisoli* JCM 15515	49.6	[46.2 - 53.0]	29.1	[26.7 - 31.6]	43.5	[40.5 - 46.5]	1.54
**'bin.20.fa'**	*Castellaniella caeni* NBRC 101664	23.4	[20.1 - 27.1]	22.7	[20.4 - 25.1]	22.3	[19.5 - 25.3]	0.41
**'bin.20.fa'**	*Bordetella pseudohinzii* 8-296-03	14.6	[11.8 - 18.0]	19.7	[17.5 - 22.1]	14.8	[12.3 - 17.6]	1.97
**'bin.20.fa'**	*Corticimicrobacter populi* 3d-2-2T	13.5	[10.7 - 16.8]	19.5	[17.3 - 21.9]	13.8	[11.4 - 16.6]	3.57
**'bin.20.fa'**	*Yanghanlia caeni* LG-2	14.4	[11.6 - 17.8]	19.1	[16.9 - 21.5]	14.6	[12.1 - 17.4]	1.72
**'bin.20.fa'**	*Vandammella animalimorsus* NML 03-0146T	13.0	[10.3 - 16.3]	18.4	[16.3 - 20.8]	13.3	[11.0 - 16.1]	0.88
**'bin.20.fa'**	*Comamonas flocculans* NLF-7-7	13.1	[10.4 - 16.4]	17.4	[15.3 - 19.7]	13.4	[11.1 - 16.2]	3.19

Table 4: Strains in your dataset

Joint dataset of automatically determined closest type strains (if this mode was chosen), manually selected type strains (if selected accordingly) and the provided user strains, if provided (marked in yellow).

Strain	Authority	Other deposits	Synonyms	Base pairs	Percent G+C	No. proteins	Goldstamp	Bioproject accession	Biosample accession	Assembly accession	IMG OID
Castellaniella defragrans CCUG 39790	(Foss et al. 1998) Kämpfer et al. 2006	54Pin; LMG 18538; CIP 105602; CCUG 39790; DSM 12141	*Alcaligenes defragrans*; *Castellaniella defragrans*	3949 625	69.2	3638		PRJNA563568	SAMN12697567	GCA_008801975	
Corticimicrobacter populi 3d-2-2T	Li et al. 2019	KCTC 52807; CFCC 11891	*Corticimicrobacter populi*	3546 680	61.0	3235		PRJNA224116	SAMN08993631	GCF_003123725	
Castellaniella defragrans DSM 12141	(Foss et al. 1998) Kämpfer et al. 2006	54Pin; LMG 18538; CIP 105602; CCUG 39790; DSM 12141	*Alcaligenes defragrans*; *Castellaniella defragrans*	3937 281	69.2	3581	Gp0456234				2861677110
Castellaniella caeni NBRC 101664	Liu et al. 2008	LMG 23411; KCTC 12197; DSM 22240; Ho-11	*Castellaniella caeni*	3275 699	65.0	2997	Gp0075762	PRJDB1344	SAMD00047219	GCA_001592225	
Bordetella pseudohinzii 8-296-03	Ivanov et al. 2016	NRRL B-59942; NCTC 13808	*Bordetella pseudohinzii*	4538 520	66.6	4183		PRJNA224116	SAMN02263900	GCF_000657795	
Yanghanlia caeni LG-2	Ruan et al. 2024	KCTC 8084; CCTCC AB 2023123	*Yanghanlia caeni*	3202 748	62.9	2963		PRJNA1002479	SAMN36845266	GCA_030733815	
Comamonas flocculans NLF-7-7	Kim et al. 2020	KCTC 62943	*Comamonas flocculans*	3333 437	67.8	3079		PRJNA555370	SAMN12317495	GCA_007954405	
Castellaniella daejeonensis JCM16240	Lee et al. 2010	KCTC 22454; DSM 25289; MJ06	*Castellaniella daejeonensis*	3101 645	66.6	2880		PRJDB10510	SAMD00645013	GCA_039523555	

Strain	Authority	Other deposits	Synonyms	Base pairs	Percent G+C	No. proteins	Goldstamp	Bioproject accession	Biosample accession	Assembly accession	IMG OID
Castellaniella ginsengisoli JCM 15515	Kim et al. 2009	KCTC 22398; DSM 23970; DCY 36	*Castellaniella ginsengisoli*	2907 155	66.1	2694		PRJDB10510	SAMD00644997	GCA_039523235	
Vandammella animalimorsus NML 03-0146T	Bernard et al. 2022	LMG 31935; NCTC 14450	*Vandammella animalimorsus*	3075 779	65.5	2631		PRJNA398363	SAMN07511443	GCA_002285285	
bin.20.fa				2605 997	64.6	2506					

Methods, Results and References

The genome sequence data were uploaded to the Type (Strain) Genome Server (TYGS), a free bioinformatics platform available under https://tygs.dsmz.de, for a whole genome-based taxonomic analysis [1]. The analysis also made use of recently introduced methodological updates and features [2]. Information on nomenclature, synonymy and associated taxonomic literature was provided by TYGS's sister database, the List of Prokaryotic names with Standing in Nomenclature (LPSN, available at https://lpsn.dsmz.de) [2]. The results were provided by the TYGS on 2024-06-16. The TYGS analysis was subdivided into the following steps:

Determination of closely related type strains

The determination of closely related type strains did not succeed because not a single 16S rDNA gene sequence was detected in the provided user genomes. The subsequent analyses are thus only based on the provided genome data and the manually selected type strains, if any.

Pairwise comparison of genome sequences

For the phylogenomic inference, all pairwise comparisons among the set of genomes were conducted using GBDP and accurate intergenomic distances inferred under the algorithm 'trimming' and distance formula d_5 [3]. 100 distance replicates were calculated each. Digital DDH values and confidence intervals were calculated using the recommended settings of the GGDC 4.0 [2,3].

Phylogenetic inference

The resulting intergenomic distances were used to infer a balanced minimum evolution tree with branch support via FASTME 2.1.6.1 including SPR postprocessing [4]. Branch support was inferred from 100 pseudo-bootstrap replicates each. The trees were rooted at the midpoint [5] and visualized with PhyD3 [6].

Type-based species and subspecies clustering

The type-based species clustering using a 70% dDDH radius around each of the 10 type strains was done as previously described [1]. The resulting groups are shown in Table 1 and 4. Subspecies clustering was done using a 79% dDDH threshold as previously introduced [7].

Results

Type-based species and subspecies clustering

The resulting species and subspecies clusters are listed in Table 4, whereas the taxonomic identification of the query strains is found in Table 1. Briefly, the clustering yielded 10 species clusters and the provided query strains were assigned to 1 of these. Moreover, user strains were located in 1 of 10 subspecies clusters.

Figure caption genome tree

Figure 2. Tree inferred with FastME 2.1.6.1 [4] from GBDP distances calculated from genome sequences. The branch lengths are scaled in terms of GBDP distance formula d_5. The numbers above branches are GBDP pseudo-bootstrap support values > 60 % from 100 replications, with an average branch support of 80.9 %. The tree was rooted at the midpoint [5].

References

[1] Meier-Kolthoff JP, Göker M. TYGS is an automated high-throughput platform for state-of-the-art genome-based taxonomy. Nat. Commun. 2019;10: 2182. DOI: 10.1038/s41467-019-10210-3

[2] Meier-Kolthoff JP, Sardà Carbasse J, Peinado-Olarte RL, Göker M. TYGS and LPSN: a database tandem for fast and reliable genome-based classification and nomenclature of prokaryotes. Nucleic Acid Res. 2022;50: D801–D807. DOI: 10.1093/nar/gkab902

[3] Meier-Kolthoff JP, Auch AF, Klenk H-P, Göker M. Genome sequence-based species delimitation with confidence intervals and improved distance functions. BMC Bioinformatics. 2013;14: 60. DOI: 10.1186/1471-2105-14-60

[4] Lefort V, Desper R, Gascuel O. FastME 2.0: A comprehensive, accurate, and fast distance-based phylogeny inference program. Mol Biol Evol. 2015;32: 2798–2800. DOI: 10.1093/molbev/msv150

[5] Farris JS. Estimating phylogenetic trees from distance matrices. Am Nat. 1972;106: 645–667.

[6] Kreft L, Botzki A, Coppens F, Vandepoele K, Van Bel M. PhyD3: A phylogenetic tree viewer with extended phyloXML support for functional genomics data visualization. Bioinformatics. 2017;33: 2946–2947. DOI: 10.1093/bioinformatics/btx324

[7] Meier-Kolthoff JP, Hahnke RL, Petersen J, Scheuner C, Michael V, Fiebig A, et al. Complete genome sequence of DSM 30083^T, the type strain (U5/41^T) of *Escherichia coli*, and a proposal for delineating subspecies in microbial taxonomy. Stand Genomic Sci. 2014;9: 2. DOI: 10.1186/1944-3277-9-2
