# Supplementary material for: Tracing active members in microbial communities by BONCAT and click chemistry-based enrichment of newly synthesized proteins
Source: ISME Commun. 2024 Dec 4;4(1):ycae153. doi: 10.1093/ismeco/ycae153 (PMC11683836; doi:10.1093/ismeco/ycae153)
Supplement: Genome_Server_ycae153 [file genome_server_ycae153.zip › Genome Server/Bin_22_TYGS_job_results.pdf]

TYPE (STRAIN) GENOME SERVER

JOB SUMMARY

PRINT DATE:	2024-06-17 08:59:05 +0200
JOB ID:	8ce6b427-b617-4191-b16f-6815bae704ea--07
RESULT PAGE:	https://tygs.dsmz.de/user_results/show?guid=8ce6b427-b617-4191-b16f-6815bae704ea--07

Table 1: Phylogenies

Publication-ready versions of both the genome-scale GBDP tree and the 16S rRNA gene sequence tree can be customized and exported either in SVG (vector graphic) or PNG format from within the phylogeny viewers in your TYGS result page. For publications the **SVG format is recommended** because it is lossless, always keeps its high resolution and can also be easily converted to other popular formats such as PDF or EPS. Please follow the link provided above!

Table 2: Identification

The below list contains the result of the TYGS species identification routine.

Explanation of remarks that might occur in the below table:

remark [R1]: The TYGS type strain database is automatically updated on an almost daily basis. However, if a particular type strain genome is not available in the TYGS database, this can have several reasons which are detailed in the FAQ. You can request an extended 16S rRNA gene analysis via the 16S tree viewer found in your result page to detect **not yet genome-sequenced** type strains relevant for your study.

remark [R2]: > 70% dDDH value (formula d_4) and (almost) minimal dDDH values for gene-content formulae d_0 and d_6 indicate a potentially unreliable identification result and should thus be checked via the 16S rRNA gene sequence similarity. Such strong deviations can, in principle, be caused by sequence contamination.

remark [R3]: G+C content difference of > 1 % indicates a potentially unreliable identification result because within species G+C content varies no more than 1 %, if computed from genome sequences (PMID: 24505073).

Strain	Conclusion	Identification result	Remark
**'bin.22'**	belongs to known species	*Fermentimonas caenicola*	see [R2]<br>see [R3]

Table 3: Pairwise comparisons of user genomes vs. type-strain genomes

The following table contains the pairwise dDDH values between your user genomes and the selected type-strain genomes. The dDDH values are provided along with their confidence intervals (C.I.) for the three different GBDP formulas:

- formula d_0 (a.k.a. GGDC formula 1): length of all HSPs divided by total genome length
- formula d_4 (a.k.a. GGDC formula 2): sum of all identities found in HSPs divided by overall HSP length
- formula d_6 (a.k.a. GGDC formula 3): sum of all identities found in HSPs divided by total genome length

Note: Formula d_4 is independent of genome length and is thus robust against the use of incomplete draft genomes. For other reasons for preferring formula d_4, see the FAQ.

Query	Subject	d_0	C.I. d_0	d_4	C.I. d_4	d_6	C.I. d_6	Diff. G+C Percent
**'bin.22.fa'**	*Lascolabacillus massiliensis* SIT8	12.5	[9.9 - 15.8]	93.1	[91.2 - 94.7]	12.9	[10.6 - 15.7]	5.43
**'bin.22.fa'**	*Fermentimonas caenicola* ING2-E5B	12.5	[9.9 - 15.8]	90.9	[88.6 - 92.7]	12.9	[10.6 - 15.7]	5.73
**'bin.22.fa'**	*Clostridium diolis* DSM 15410	12.7	[10.0 - 15.9]	53.3	[50.6 - 55.9]	13.1	[10.7 - 15.8]	1.83
**'bin.22.fa'**	*Clostridium beijerinckii* DSM 791	12.7	[10.0 - 15.9]	47.4	[44.8 - 50.0]	13.1	[10.7 - 15.8]	1.91
**'bin.22.fa'**	*Clostridium beijerinckii* NCTC 13035	12.7	[10.0 - 15.9]	46.1	[43.5 - 48.7]	13.1	[10.7 - 15.8]	1.69
**'bin.22.fa'**	*Citroniella saccharovorans* DSM 29873	12.5	[9.8 - 15.7]	27.1	[24.8 - 29.6]	12.9	[10.6 - 15.6]	1.34
**'bin.22.fa'**	*Clostridium tepidum* IEH 97212	12.6	[9.9 - 15.8]	26.6	[24.3 - 29.1]	13.0	[10.7 - 15.7]	4.49
**'bin.22.fa'**	*Treponema phagedenis* B43.1 T	12.5	[9.8 - 15.7]	21.6	[19.3 - 24.0]	12.9	[10.6 - 15.6]	8.34
**'bin.22.fa'**	*Clostridium akagii* DSM 12554	12.5	[9.9 - 15.8]	21.2	[19.0 - 23.7]	12.9	[10.6 - 15.7]	0.83
**'bin.22.fa'**	*Peptoniphilus obesi* ph1	12.5	[9.8 - 15.7]	3.7	[2.8 - 4.8]	12.9	[10.6 - 15.6]	1.43

Table 4: Strains in your dataset

Joint dataset of automatically determined closest type strains (if this mode was chosen), manually selected type strains (if selected accordingly) and the provided user strains, if provided (marked in yellow).

Strain	Authority	Other deposits	Synonyms	Base pairs	Percent G+C	No. proteins	Goldstamp	Bioproject accession	Biosample accession	Assembly accession	IMG OID
Citroniella saccharovorans DSM 29873	Patel et al. 2019	CCUG 66799; M6.X9	*Citroniella saccharovorans*	1953 712	30.2	1814		PRJNA1050169	SAMN38726626	GCA_035481615	
Clostridium tepidum IEH 97212	Dobritsa et al. 2017	NRRL B-65463; DSM 104389	*Clostridium tepidum*	3074 397	27.1	2718		PRJNA224116	SAMN06094717	GCF_002008345	
Treponema phagedenis B43.1 T	(ex Brumpt 1922) Kuhnert et al. 2020	DSM 110455; NCTC 14362	*Treponema phagedenis*	3372 116	39.9	2631		PRJNA224116	SAMN12512735	GCF_008153345	
Clostridium akagii DSM 12554	Kuhner et al. 2000	ATCC BAA-166; CK58	*Clostridium akagii*	4587 056	30.7	4346	Gp0013477	PRJNA234894	SAMN02743371	GCA_000686725	2563366732
Peptoniphilus obesi ph1	Mishra et al. 2013	DSM 25489; CSUR P187	*Peptoniphilus obesi*	1746 609	30.1	1669		PRJEA82275	SAMEA2272531	GCA_000311865	
Clostridium beijerinckii NCTC 13035	Donker 1926 emend. Kobayashi et al. 2020	LMG 5716; CIP 104308; ATCC 25752; DSM 791; JCM 1390; NCTC 13035	*Clostridium beijerinckii*	6010 505	29.9	5266	Gp0342047	PRJEB6403	SAMEA4530646	GCA_900447025	
Clostridium beijerinckii DSM 791	Donker 1926 emend. Kobayashi et al. 2020	LMG 5716; CIP 104308; ATCC 25752; DSM 791; JCM 1390; NCTC 13035	*Clostridium beijerinckii*	5781 472	29.7	5081		PRJNA322803	SAMN05170524	GCA_002006445	
Lascolabacillus massiliensis SIT8	Beye et al. 2016	CSUR P1560	*Lascolabacillus massiliensis*	2824 716	37.0	2344		PRJEB8934	SAMEA3312814	GCA_001282625	

Strain	Authority	Other deposits	Synonyms	Base pairs	Percent G+C	No. proteins	Goldstamp	Bioproject accession	Biosample accession	Assembly accession	IMG OID
Clostridium diolis DSM 15410	Biebl and Spröer 2003	ATCC BAA-557; DSM 5431; SH1	*Clostridium diolis*	5853 885	29.7	5114	Gp0041667	PRJNA196562	SAMN02470960	GCA_000409695	2554235113
Fermentimonas caenicola ING2-E5B	Hahnke et al. 2016	LMG 28429; CECT 8609; DSM 28696	*Fermentimonas caenicola*	2808 926	37.3	2405	Gp0122603	PRJEB7091	SAMEA2724983	GCA_000953535	
bin.22.fa				4352 215	31.6	4899					

Methods, Results and References

The genome sequence data were uploaded to the Type (Strain) Genome Server (TYGS), a free bioinformatics platform available under https://tygs.dsmz.de, for a whole genome-based taxonomic analysis [1]. The analysis also made use of recently introduced methodological updates and features [2]. Information on nomenclature, synonymy and associated taxonomic literature was provided by TYGS's sister database, the List of Prokaryotic names with Standing in Nomenclature (LPSN, available at https://lpsn.dsmz.de) [2]. The results were provided by the TYGS on 2024-06-16. The TYGS analysis was subdivided into the following steps:

Determination of closely related type strains

The determination of closely related type strains did not succeed because not a single 16S rDNA gene sequence was detected in the provided user genomes. The subsequent analyses are thus only based on the provided genome data and the manually selected type strains, if any.

Pairwise comparison of genome sequences

For the phylogenomic inference, all pairwise comparisons among the set of genomes were conducted using GBDP and accurate intergenomic distances inferred under the algorithm 'trimming' and distance formula d_5 [3]. 100 distance replicates were calculated each. Digital DDH values and confidence intervals were calculated using the recommended settings of the GGDC 4.0 [2,3].

Phylogenetic inference

The resulting intergenomic distances were used to infer a balanced minimum evolution tree with branch support via FASTME 2.1.6.1 including SPR postprocessing [4]. Branch support was inferred from 100 pseudo-bootstrap replicates each. The trees were rooted at the midpoint [5] and visualized with PhyD3 [6].

Type-based species and subspecies clustering

The type-based species clustering using a 70% dDDH radius around each of the 10 type strains was done as previously described [1]. The resulting groups are shown in Table 1 and 4. Subspecies clustering was done using a 79% dDDH threshold as previously introduced [7].

Results

Type-based species and subspecies clustering

The resulting species and subspecies clusters are listed in Table 4, whereas the taxonomic identification of the query strains is found in Table 1. Briefly, the clustering yielded 7 species clusters and the provided query strains were assigned to 1 of these. Moreover, user strains were located in 1 of 7 subspecies clusters.

Figure caption genome tree

Figure 2. Tree inferred with FastME 2.1.6.1 [4] from GBDP distances calculated from genome sequences. The branch lengths are scaled in terms of GBDP distance formula d_5. The numbers above branches are GBDP pseudo-bootstrap support values > 60 % from 100 replications, with an average branch support of 91.5 %. The tree was rooted at the midpoint [5].

References

[1] Meier-Kolthoff JP, Göker M. TYGS is an automated high-throughput platform for state-of-the-art genome-based taxonomy. Nat. Commun. 2019;10: 2182. DOI: 10.1038/s41467-019-10210-3

[2] Meier-Kolthoff JP, Sardà Carbasse J, Peinado-Olarte RL, Göker M. TYGS and LPSN: a database tandem for fast and reliable genome-based classification and nomenclature of prokaryotes. Nucleic Acid Res. 2022;50: D801–D807. DOI: 10.1093/nar/gkab902

[3] Meier-Kolthoff JP, Auch AF, Klenk H-P, Göker M. Genome sequence-based species delimitation with confidence intervals and improved distance functions. BMC Bioinformatics. 2013;14: 60. DOI: 10.1186/1471-2105-14-60

[4] Lefort V, Desper R, Gascuel O. FastME 2.0: A comprehensive, accurate, and fast distance-based phylogeny inference program. Mol Biol Evol. 2015;32: 2798–2800. DOI: 10.1093/molbev/msv150

[5] Farris JS. Estimating phylogenetic trees from distance matrices. Am Nat. 1972;106: 645–667.

[6] Kreft L, Botzki A, Coppens F, Vandepoele K, Van Bel M. PhyD3: A phylogenetic tree viewer with extended phyloXML support for functional genomics data visualization. Bioinformatics. 2017;33: 2946–2947. DOI: 10.1093/bioinformatics/btx324

[7] Meier-Kolthoff JP, Hahnke RL, Petersen J, Scheuner C, Michael V, Fiebig A, et al. Complete genome sequence of DSM 30083^T, the type strain (U5/41^T) of *Escherichia coli*, and a proposal for delineating subspecies in microbial taxonomy. Stand Genomic Sci. 2014;9: 2. DOI: 10.1186/1944-3277-9-2
