# Supplementary material for: Tracing active members in microbial communities by BONCAT and click chemistry-based enrichment of newly synthesized proteins
Source: ISME Commun. 2024 Dec 4;4(1):ycae153. doi: 10.1093/ismeco/ycae153 (PMC11683836; doi:10.1093/ismeco/ycae153)
Supplement: Genome_Server_ycae153 [file genome_server_ycae153.zip › Genome Server/Bin_25_TYGS_job_results.pdf]

TYPE (STRAIN) GENOME SERVER

JOB SUMMARY

PRINT DATE: 2024-06-17 09:02:01 +0200

JOB ID: 8ce6b427-b617-4191-b16f-6815bae704ea--10

RESULT PAGE: https://tygs.dsmz.de/user_results/show?guid=8ce6b427-b617-4191-b16f-6815bae704ea--10

Table 1: Phylogenies

Publication-ready versions of both the genome-scale GBDP tree and the 16S rRNA gene sequence tree can be customized and exported either in SVG (vector graphic) or PNG format from within the phylogeny viewers in your TYGS result page. For publications the **SVG format is recommended** because it is lossless, always keeps its high resolution and can also be easily converted to other popular formats such as PDF or EPS. Please follow the link provided above!

Table 2: Identification

The below list contains the result of the TYGS species identification routine.

Explanation of remarks that might occur in the below table:

remark [R1]: The TYGS type strain database is automatically updated on an almost daily basis. However, if a particular type strain genome is not available in the TYGS database, this can have several reasons which are detailed in the FAQ. You can request an extended 16S rRNA gene analysis via the 16S tree viewer found in your result page to detect **not yet genome-sequenced** type strains relevant for your study.

remark [R2]: > 70% dDDH value (formula d_4) and (almost) minimal dDDH values for gene-content formulae d_0 and d_6 indicate a potentially unreliable identification result and should thus be checked via the 16S rRNA gene sequence similarity. Such strong deviations can, in principle, be caused by sequence contamination.

remark [R3]: G+C content difference of > 1 % indicates a potentially unreliable identification result because within species G+C content varies no more than 1 %, if computed from genome sequences (PMID: 24505073).

Strain	Conclusion	Identification result	Remark
**'bin.25'**	belongs to known species	*Candidatus* Brevifilum fermentans	see [R3]

Table 3: Pairwise comparisons of user genomes vs. type-strain genomes

The following table contains the pairwise dDDH values between your user genomes and the selected type-strain genomes. The dDDH values are provided along with their confidence intervals (C.I.) for the three different GBDP formulas:

• formula d_0 (a.k.a. GGDC formula 1): length of all HSPs divided by total genome length
• formula d_4 (a.k.a. GGDC formula 2): sum of all identities found in HSPs divided by overall HSP length
• formula d_6 (a.k.a. GGDC formula 3): sum of all identities found in HSPs divided by total genome length

Note: Formula d_4 is independent of genome length and is thus robust against the use of incomplete draft genomes. For other reasons for preferring formula d_4, see the FAQ.

Query	Subject	d_0	C.I. d_0	d_4	C.I. d_4	d_6	C.I. d_6	Diff. G+C Percent
**'bin.25.fa'**	*Candidatus* Brevifilum fermentans CAMBI-1	81.8	[77.9 - 85.2]	99.8	[99.7 - 99.9]	87.8	[84.8 - 90.3]	1.38
**'bin.25.fa'**	*Levilinea saccharolytica* DSM 16555	12.5	[9.8 - 15.8]	36.4	[34.0 - 39.0]	12.9	[10.6 - 15.6]	9.29
**'bin.25.fa'**	*Leptolinea tardivitalis* DSM 16556	12.5	[9.8 - 15.8]	31.1	[28.7 - 33.6]	12.9	[10.6 - 15.7]	3.7
**'bin.25.fa'**	*Anaerolinea thermophila* UNI-1	12.5	[9.9 - 15.8]	30.7	[28.3 - 33.2]	12.9	[10.6 - 15.7]	3.34
**'bin.25.fa'**	*Thermanaerothrix daxensis* DSM 23592	12.5	[9.8 - 15.8]	27.2	[24.8 - 29.7]	12.9	[10.6 - 15.7]	5.45
**'bin.25.fa'**	*Ornatilinea apprima* DSM 23815	12.5	[9.9 - 15.8]	27.2	[24.8 - 29.7]	12.9	[10.6 - 15.7]	4.52
**'bin.25.fa'**	*Longilinea arvoryzae* KOME-1	12.5	[9.9 - 15.8]	25.2	[22.9 - 27.7]	12.9	[10.6 - 15.7]	6.41
**'bin.25.fa'**	*Pelolinea submarina* DSM 23923	12.5	[9.8 - 15.8]	25.0	[22.7 - 27.5]	12.9	[10.6 - 15.7]	0.11
**'bin.25.fa'**	*Bellilinea caldifistulae* DSM 17877	12.5	[9.9 - 15.8]	24.4	[22.1 - 26.9]	12.9	[10.6 - 15.7]	2.31
**'bin.25.fa'**	*Halopseudomonas sabulinigri* JCM 14963	12.5	[9.8 - 15.8]	21.1	[18.8 - 23.5]	12.9	[10.6 - 15.6]	9.4
**'bin.25.fa'**	*Anaerolinea thermolimosa* IMO-1	12.5	[9.9 - 15.8]	20.6	[18.4 - 23.1]	12.9	[10.6 - 15.7]	4.11
**'bin.25.fa'**	*Pseudomonas triclosanedens* ZM23	12.5	[9.8 - 15.8]	20.0	[17.8 - 22.4]	12.9	[10.6 - 15.6]	13.48
**'bin.25.fa'**	*Halopseudomonas xiamenensis* DSM 22326	12.5	[9.8 - 15.7]	19.9	[17.7 - 22.3]	12.9	[10.6 - 15.6]	11.65
**'bin.25.fa'**	*Dietzia aerolata* Sj14a	12.5	[9.8 - 15.7]	19.8	[17.6 - 22.2]	12.9	[10.6 - 15.6]	17.67
**'bin.25.fa'**	*Zunongwangia profunda* SM-A87	12.5	[9.8 - 15.8]	18.8	[16.6 - 21.2]	12.9	[10.6 - 15.6]	14.29
**'bin.25.fa'**	*Cupriavidus nantongensis* X1	12.5	[9.8 - 15.8]	18.0	[15.8 - 20.3]	12.9	[10.6 - 15.7]	16.21
**'bin.25.fa'**	*Cupriavidus laharis* LMG 23992	12.5	[9.8 - 15.8]	17.7	[15.5 - 20.0]	12.9	[10.6 - 15.6]	14.7
**'bin.25.fa'**	*Candidatus* Pelethomonas intestinigallinarum ChiSjej2B20-3600	12.5	[9.8 - 15.7]	3.7	[2.8 - 4.8]	12.9	[10.6 - 15.6]	13.84
**'bin.25.fa'**	*Microbulbifer donghaiensis* CGMCC 1.7063	12.5	[9.8 - 15.7]	3.7	[2.8 - 4.8]	12.9	[10.6 - 15.6]	9.18

Table 4: Strains in your dataset

Joint dataset of automatically determined closest type strains (if this mode was chosen), manually selected type strains (if selected accordingly) and the provided user strains, if provided (marked in yellow).

Strain	Authority	Other deposits	Synonyms	Base pairs	Percent G+C	No. proteins	Goldstamp	Bioproject accession	Biosample accession	Assembly accession	IMG OID
Pseudomonas triclosanedens ZM23	Yin et al. 2024	MCCC 1K08497; JCM 36056	*Pseudomonas triclosanedens*	6245 384	64.0	5633		PRJNA905558	SAMN31875046	GCA_026686735	
Candidatus Brevifilum fermentans CAMBI-1	McIlroy et al. 2017		*Candidatus* Brevifilum fermentans	2574 431	49.1	2159		PRJNA224116	SAMEA104092011	GCF_900184705	
Candidatus Pelethomonas intestinigallinaru m ChiSjej2B20-3600	Gilroy et al. 2021		*Candidatus* Pelethomonas intestinigallinaru m	2034 372	64.4	1945		PRJNA543206	SAMN15817014	GCA_018712665	
Microbulbifer donghaiensis CGMCC 1.7063	Wang et al. 2009	JCM 15145; CN 85	*Microbulbifer donghaiensis*	4313 617	59.7	3620	Gp0112574	PRJEB18177	SAMN04487965	GCA_900129095	
Zunongwangia profunda SM-A87	Qin et al. 2007	DSM 18752; CCTCC AB 206139	*Zunongwangia profunda*	5128 187	36.2	4653	Gp0005129	PRJNA38641	SAMN02603855	GCA_000023465	646564591
Pelolinea submarina DSM 23923	Imachi et al. 2014	DSM 28969; JCM 17238; MO-CFX1	*Pelolinea submarina*	3518 991	50.6	3095	Gp0290646	PRJNA463391	SAMN09074695	GCA_003385075	2770939606
Halopseudomon as xiamenensis DSM 22326	(Lai and Shao 2008) Rudra and Gupta 2021	MCCC 1A00089; CGMCC 1.6446; JCM 13530; C10-2	*Halopseudomon as xiamenensis*; *Pseudomonas xiamenensis*	4268 146	62.2	3854	Gp0013197	PRJNA235122	SAMN02745750		2576861402
Thermanaerothri x daxensis DSM 23592	Grégoire et al. 2011	JCM 16980; GNS-1	*Thermanaerothri x daxensis*	3012 066	56.0	2395	Gp0118393	PRJNA271542	SAMN03274859	GCA_001306145	

Strain	Authority	Other deposits	Synonyms	Base pairs	Percent G+C	No. proteins	Goldstamp	Bioproject accession	Biosample accession	Assembly accession	IMG OID
Anaerolinea thermophila UNI-1	Sekiguchi et al. 2003	DSM 14523; JCM 11388; NBRC 100420	*Anaerolinea thermophila*	3532 378	53.8	3167	Gp0000937	PRJNA60099	SAMD00061114	GCA_000199675	649633005
Dietzia aerolata Sj14a	Kämpfer et al. 2010	DSM 45334; CCM 7659	*Dietzia aerolata*	3335 988	68.2	3228		PRJNA607608	SAMN14141888	GCA_014144865	
Anaerolinea thermolimosa IMO-1	Yamada et al. 2006 emend. Nouioui et al. 2018	DSM 16554; JCM 12577	*Anaerolinea thermolimosa*	4104 644	54.6	3699		PRJDB4019	SAMD00034885	GCA_001050195	
Bellilinea caldifistulae DSM 17877	Yamada et al. 2007 emend. Nouioui et al. 2018	JCM 13669; GOMI-1	*Bellilinea caldifistulae*	3655 684	52.8	2990		PRJNA289119	SAMN03842211	GCA_001306055	
Leptolinea tardivitalis DSM 16556	Yamada et al. 2006 emend. Nouioui et al. 2018	JCM 12579; YMTK-2	*Leptolinea tardivitalis*	3676 575	46.8	2939		PRJNA289671	SAMN03856817	GCA_001306095	
Levilinea saccharolytica DSM 16555	Yamada et al. 2006	JCM 12578; KIBI-1	*Levilinea saccharolytica*	4230 078	59.8	3173	Gp0149557	PRJNA289680	SAMN03856835	GCA_001306035	
Longilinea arvoryzae KOME-1	Yamada et al. 2007	DSM 17881; JCM 13670; KTCC 5380	*Longilinea arvoryzae*	4431 449	56.9	3886		PRJDB4023	SAMD00034889	GCA_001050235	
Ornatilinea apprima DSM 23815	Podosokorskaya et al. 2013	VKM B-2669; P3M-1	*Ornatilinea apprima*	4338 330	55.0	3347		PRJNA289676	SAMN03856825	GCA_001306115	
Halopseudomonas sabulinigri JCM 14963	(Kim et al. 2009) Rudra and Gupta 2021	KCTC 22137; DSM 23971; J64	*Halopseudomonas sabulinigri*; *Neopseudomonas sabulinigri*; *Pseudomonas sabulinigri*	4030 103	59.9	3624	Gp0127171	PRJEB16439	SAMN05216271	GCA_900105255	
Cupriavidus nantongensis X1	Sun et al. 2016	LMG 29218; KCTC 42909	*Cupriavidus nantongensis*	7136 380	66.7	6187	Gp0145281	PRJNA224116	SAMN04566111	GCF_001598055	

Strain	Authority	Other deposits	Synonyms	Base pairs	Percent G+C	No. proteins	Goldstamp	Bioproject accession	Biosample accession	Assembly accession	IMG OID
Cupriavidus laharis LMG 23992	Sato et al. 2006	1263a; CIP 108726; DSM 19552; PNCM 10347	*Cupriavidus laharis*	6098 987	65.2	5526		PRJEB43925	SAMEA9552242	GCA_914271435	
bin.25.fa				2007 175	50.5	1735					

Methods, Results and References

The genome sequence data were uploaded to the Type (Strain) Genome Server (TYGS), a free bioinformatics platform available under https://tygs.dsmz.de, for a whole genome-based taxonomic analysis [1]. The analysis also made use of recently introduced methodological updates and features [2]. Information on nomenclature, synonymy and associated taxonomic literature was provided by TYGS's sister database, the List of Prokaryotic names with Standing in Nomenclature (LPSN, available at https://lpsn.dsmz.de) [2]. The results were provided by the TYGS on 2024-06-16. The TYGS analysis was subdivided into the following steps:

Determination of closely related type strains

Determination of closest type strain genomes was done in two complementary ways: First, all user genomes were compared against all type strain genomes available in the TYGS database via the MASH algorithm, a fast approximation of intergenomic relatedness [3], and, the ten type strains with the smallest MASH distances chosen per user genome. Second, an additional set of ten closely related type strains was determined via the 16S rDNA gene sequences. These were extracted from the user genomes using RNAmmer [4] and each sequence was subsequently BLASTed [5] against the 16S rDNA gene sequence of each of the currently 21253 type strains available in the TYGS database. This was used as a proxy to find the best 50 matching type strains (according to the bitscore) for each user genome and to subsequently calculate precise distances using the Genome BLAST Distance Phylogeny approach (GBDP) under the algorithm 'coverage' and distance formula d_5 [6]. These distances were finally used to determine the 10 closest type strain genomes for each of the user genomes.

Pairwise comparison of genome sequences

For the phylogenomic inference, all pairwise comparisons among the set of genomes were conducted using GBDP and accurate intergenomic distances inferred under the algorithm 'trimming' and distance formula d_5 [6]. 100 distance replicates were calculated each. Digital DDH values and confidence intervals were calculated using the recommended settings of the GGDC 4.0 [2,6].

Phylogenetic inference

The resulting intergenomic distances were used to infer a balanced minimum evolution tree with branch support via FASTME 2.1.6.1 including SPR postprocessing [7]. Branch support was inferred from 100 pseudo-bootstrap replicates each. The trees were rooted at the midpoint [8] and visualized with PhyD3 [9].

Type-based species and subspecies clustering

The type-based species clustering using a 70% dDDH radius around each of the 19 type strains was done as previously described [1]. The resulting groups are shown in Table 1 and 4. Subspecies clustering was done using a 79% dDDH threshold as previously introduced [10].

Results

Type-based species and subspecies clustering

The resulting species and subspecies clusters are listed in Table 4, whereas the taxonomic identification of the query strains is found in Table 1. Briefly, the clustering yielded 19 species clusters and the provided query strains were assigned to 1 of these. Moreover, user strains were located in 1 of 19 subspecies clusters.

Figure caption SSU tree

Figure 1. Tree inferred with FastME 2.1.6.1 [7] from GBDP distances calculated from 16S rDNA gene sequences. The branch lengths are scaled in terms of GBDP distance formula d_5. The numbers above branches are GBDP pseudo-bootstrap support values > 60 % from 100 replications, with an average branch support of 74.3 %. The tree was rooted at the midpoint [8].

Figure caption genome tree

Figure 2. Tree inferred with FastME 2.1.6.1 [7] from GBDP distances calculated from genome sequences. The branch lengths are scaled in terms of GBDP distance formula d_5. The numbers above branches are GBDP pseudo-bootstrap support values > 60 % from 100 replications, with an average branch support of 55.9 %. The tree was rooted at the midpoint [8].

References

[1] Meier-Kolthoff JP, Göker M. TYGS is an automated high-throughput platform for state-of-the-art genome-based taxonomy. Nat. Commun. 2019;10: 2182. DOI: 10.1038/s41467-019-10210-3

[2] Meier-Kolthoff JP, Sardà Carbasse J, Peinado-Olarte RL, Göker M. TYGS and LPSN: a database tandem for fast and reliable genome-based classification and nomenclature of prokaryotes. Nucleic Acid Res. 2022;50: D801–D807. DOI: 10.1093/nar/gkab902

[3] Ondov BD, Treangen TJ, Melsted P, et al. Mash: Fast genome and metagenome distance estimation using MinHash. Genome Biol 2016;17: 1–14. DOI: 10.1186/s13059-016-0997-x

[4] Lagesen K, Hallin P. RNAmmer: consistent and rapid annotation of ribosomal RNA genes. Nucleic Acids Res. Oxford Univ Press; 2007;35: 3100–3108. DOI: 10.1093/nar/gkm160

[5] Camacho C, Coulouris G, Avagyan V, Ma N, Papadopoulos J, Bealer K, et al. BLAST+: architecture and applications. BMC Bioinformatics. 2009;10: 421. DOI: 10.1186/1471-2105-10-421

[6] Meier-Kolthoff JP, Auch AF, Klenk H-P, Göker M. Genome sequence-based species delimitation with confidence intervals and improved distance functions. BMC Bioinformatics. 2013;14: 60. DOI: 10.1186/1471-2105-14-60

[7] Lefort V, Desper R, Gascuel O. FastME 2.0: A comprehensive, accurate, and fast distance-based phylogeny inference program. Mol Biol Evol. 2015;32: 2798–2800. DOI: 10.1093/molbev/msv150

[8] Farris JS. Estimating phylogenetic trees from distance matrices. Am Nat. 1972;106: 645–667.

[9] Kreft L, Botzki A, Coppens F, Vandepoele K, Van Bel M. PhyD3: A phylogenetic tree viewer with extended phyloXML support for functional genomics data visualization. Bioinformatics. 2017;33: 2946–2947. DOI: 10.1093/bioinformatics/btx324

[10] Meier-Kolthoff JP, Hahnke RL, Petersen J, Scheuner C, Michael V, Fiebig A, et al. Complete genome sequence of DSM 30083^T, the type strain (U5/41^T) of *Escherichia coli*, and a proposal for delineating subspecies in microbial taxonomy. Stand Genomic Sci. 2014;9: 2. DOI: 10.1186/1944-3277-9-2
