# Supplementary material for: Tracing active members in microbial communities by BONCAT and click chemistry-based enrichment of newly synthesized proteins
Source: ISME Commun. 2024 Dec 4;4(1):ycae153. doi: 10.1093/ismeco/ycae153 (PMC11683836; doi:10.1093/ismeco/ycae153)
Supplement: Genome_Server_ycae153 [file genome_server_ycae153.zip › Genome Server/Bin_27_TYGS_job_results.pdf]

TYPE (STRAIN) GENOME SERVER

JOB SUMMARY

PRINT DATE: 2024-06-17 09:04:14 +0200

JOB ID: 8ce6b427-b617-4191-b16f-6815bae704ea--11

RESULT PAGE: https://tygs.dsmz.de/user_results/show?guid=8ce6b427-b617-4191-b16f-6815bae704ea--11

Table 1: Phylogenies

Publication-ready versions of both the genome-scale GBDP tree and the 16S rRNA gene sequence tree can be customized and exported either in SVG (vector graphic) or PNG format from within the phylogeny viewers in your TYGS result page. For publications the **SVG format is recommended** because it is lossless, always keeps its high resolution and can also be easily converted to other popular formats such as PDF or EPS. Please follow the link provided above!

Table 2: Identification

The below list contains the result of the TYGS species identification routine.

Explanation of remarks that might occur in the below table:

remark [R1]: The TYGS type strain database is automatically updated on an almost daily basis. However, if a particular type strain genome is not available in the TYGS database, this can have several reasons which are detailed in the FAQ. You can request an extended 16S rRNA gene analysis via the 16S tree viewer found in your result page to detect **not yet genome-sequenced** type strains relevant for your study.

remark [R2]: > 70% dDDH value (formula d_4) and (almost) minimal dDDH values for gene-content formulae d_0 and d_6 indicate a potentially unreliable identification result and should thus be checked via the 16S rRNA gene sequence similarity. Such strong deviations can, in principle, be caused by sequence contamination.

remark [R3]: G+C content difference of > 1 % indicates a potentially unreliable identification result because within species G+C content varies no more than 1 %, if computed from genome sequences (PMID: 24505073).

Strain	Conclusion	Identification result	Remark
**'bin.27'**	potential new species		see [R1]

Table 3: Pairwise comparisons of user genomes vs. type-strain genomes

The following table contains the pairwise dDDH values between your user genomes and the selected type-strain genomes. The dDDH values are provided along with their confidence intervals (C.I.) for the three different GBDP formulas:

- formula d_0 (a.k.a. GGDC formula 1): length of all HSPs divided by total genome length
- formula d_4 (a.k.a. GGDC formula 2): sum of all identities found in HSPs divided by overall HSP length
- formula d_6 (a.k.a. GGDC formula 3): sum of all identities found in HSPs divided by total genome length

Note: Formula d_4 is independent of genome length and is thus robust against the use of incomplete draft genomes. For other reasons for preferring formula d_4, see the FAQ.

Query	Subject	d_0	C.I. d_0	d_4	C.I. d_4	d_6	C.I. d_6	Diff. G+C Percent
**'bin.27.fa'**	*Luteococcus peritonei* JCM 11685	13.2	[10.4 - 16.5]	19.4	[17.2 - 21.7]	13.5	[11.1 - 16.3]	3.63
**'bin.27.fa'**	*Propioniciclava coleopterorum* JCM 33680T	14.7	[11.8 - 18.1]	19.2	[17.0 - 21.6]	14.8	[12.3 - 17.6]	5.19
**'bin.27.fa'**	*Aestuariimicrobium ganziense* YIM S02566T	13.3	[10.5 - 16.6]	19.1	[16.9 - 21.5]	13.6	[11.2 - 16.4]	1.96
**'bin.27.fa'**	*Propioniciclava tarda* DSM 22130	14.1	[11.3 - 17.5]	19.0	[16.8 - 21.4]	14.3	[11.9 - 17.1]	2.47
**'bin.27.fa'**	*Nocardioides ganghwensis* 9920	12.8	[10.1 - 16.1]	18.7	[16.5 - 21.1]	13.2	[10.8 - 15.9]	5.16
**'bin.27.fa'**	*Actinomadura bangladeshensis* DSM 45347	12.6	[10.0 - 15.9]	18.5	[16.3 - 20.9]	13.0	[10.7 - 15.8]	5.24
**'bin.27.fa'**	*Saccharomonospora iraqiensis* subsp. *paurometabolica* YIM90007	12.6	[10.0 - 15.9]	18.2	[16.1 - 20.6]	13.0	[10.7 - 15.8]	5.06
**'bin.27.fa'**	*Phycicoccus duodecadis* DSM 12806	12.8	[10.2 - 16.1]	17.9	[15.8 - 20.3]	13.2	[10.9 - 16.0]	6.71
**'bin.27.fa'**	*Nocardioides terrae* CGMCC 1.7056	12.8	[10.1 - 16.1]	17.8	[15.7 - 20.2]	13.1	[10.8 - 15.9]	4.62
**'bin.27.fa'**	*Nocardioides cynanchi* SB3-45	12.8	[10.1 - 16.1]	17.3	[15.2 - 19.6]	13.2	[10.8 - 15.9]	4.7

Table 4: Strains in your dataset

Joint dataset of automatically determined closest type strains (if this mode was chosen), manually selected type strains (if selected accordingly) and the provided user strains, if provided (marked in yellow).

Strain	Authority	Other deposits	Synonyms	Base pairs	Percent G+C	No. proteins	Goldstamp	Bioproject accession	Biosample accession	Assembly accession	IMG OID
Propioniciclava coleopterorum JCM 33680T	(Hyun et al. 2021) Li et al. 2022	KCTC 49320; HDW11; KACC 21335	*Brevilactibacter coleopterorum*; *Propioniciclava coleopterorum*	3671 901	72.1	3104		PRJNA609859	SAMN14260667	GCA_011393335	
Aestuariimicrobium ganziense YIM S02566T	Geng et al. 2021	CGMCC 1.18751; KCTC 49477	*Aestuariimicrobium ganziense*	3145 282	68.9	2976		PRJNA224116	SAMN16229861	GCF_015352975	
Nocardioides cynanchi SB3-45	Suh et al. 2021	KCTC 49133; NBRC 114107	*Nocardioides cynanchi*	3998 545	71.6	3867		PRJNA528366	SAMN11180602	GCA_008761635	
Nocardioides terrae CGMCC 1.7056	Zhang et al. 2009	DSM 28109; JCM 16799; NBRC 104259; VA15	*Nocardioides terrae*	3921 515	71.5	3710	Gp0112578	PRJEB17184	SAMN04487968	GCA_900112345	
Luteococcus peritonei JCM 11685	Collins et al. 2000	CIP 106441; ATCC BAA-60; CCUG 38120; DSM 15826; CCM 7172	*Luteococcus peritonei*	3311 412	70.5	3092		PRJDB10510	SAMD00645101	GCA_039526385	
Nocardioides ganghwensis 9920	Yi and Chun 2004	KCTC 9920; DSM 16411; JCM 12124; IMSNU 14028; JC2055	*Nocardioides ganghwensis*	4499 152	72.1	4235		PRJNA224116	SAMN16223356	GCF_014779655	
Actinomadura bangladeshensis DSM 45347	Ara et al. 2008	3-46-b(3); JCM 13933; MTCC 8057	*Actinomadura bangladeshensis*	8675 232	72.1	7899		PRJNA524765	SAMN11032588	GCA_004348335	

Strain	Authority	Other deposits	Synonyms	Base pairs	Percent G+C	No. proteins	Goldstamp	Bioproject accession	Biosample accession	Assembly accession	IMG OID
Propioniciclava tarda DSM 22130	Sugawara et al. 2011 emend. Li et al. 2021	JCM 15804; WR 61	*Propioniciclava tarda*	2974 143	69.4	2786	Gp0194200	PRJEB20678	SAMN06266982	GCA_900182665	2724679781
Phycicoccus duodecadis DSM 12806	(Lochhead 1958) Nouioui et al. 2018	LMG 17308; CIP 102696; NCIMB 9222; ATCC 13347; JCM 11564; IFO 12959; NBRC 12959; VKM Ac-1108; IAM 14868; NCIB 9222	*Arthrobacter duodecadis*; *Phycicoccus duodecadis*; *Tetrasphaera duodecadis*	3500 352	73.6	3212	Gp0117072	PRJNA303845	SAMN04489763	GCA_002846495	2630968219
Saccharomonospora iraqiensis subsp. *paurometabolica* YIM90007	(Li et al. 2003) Nouioui et al. 2018	BCRC 16315; CCRC 16315; DSM 44619; JCM 13241; CCTCC AA 1018	*Saccharomonospora iraqiensis* subsp. *paurometabolica*; *Saccharomonospora paurometabolica*	4574 497	72.0	4649	Gp0007947	PRJNA61823	SAMN02256527	GCA_000231035	2510461000
bin.27.fa				2191 639	66.9	2185					

Methods, Results and References

The genome sequence data were uploaded to the Type (Strain) Genome Server (TYGS), a free bioinformatics platform available under https://tygs.dsmz.de, for a whole genome-based taxonomic analysis [1]. The analysis also made use of recently introduced methodological updates and features [2]. Information on nomenclature, synonymy and associated taxonomic literature was provided by TYGS's sister database, the List of Prokaryotic names with Standing in Nomenclature (LPSN, available at https://lpsn.dsmz.de) [2]. The results were provided by the TYGS on 2024-06-16. The TYGS analysis was subdivided into the following steps:

Determination of closely related type strains

The determination of closely related type strains did not succeed because not a single 16S rDNA gene sequence was detected in the provided user genomes. The subsequent analyses are thus only based on the provided genome data and the manually selected type strains, if any.

Pairwise comparison of genome sequences

For the phylogenomic inference, all pairwise comparisons among the set of genomes were conducted using GBDP and accurate intergenomic distances inferred under the algorithm 'trimming' and distance formula d_5 [3]. 100 distance replicates were calculated each. Digital DDH values and confidence intervals were calculated using the recommended settings of the GGDC 4.0 [2,3].

Phylogenetic inference

The resulting intergenomic distances were used to infer a balanced minimum evolution tree with branch support via FASTME 2.1.6.1 including SPR postprocessing [4]. Branch support was inferred from 100 pseudo-bootstrap replicates each. The trees were rooted at the midpoint [5] and visualized with PhyD3 [6].

Type-based species and subspecies clustering

The type-based species clustering using a 70% dDDH radius around each of the 10 type strains was done as previously described [1]. The resulting groups are shown in Table 1 and 4. Subspecies clustering was done using a 79% dDDH threshold as previously introduced [7].

Results

Type-based species and subspecies clustering

The resulting species and subspecies clusters are listed in Table 4, whereas the taxonomic identification of the query strains is found in Table 1. Briefly, the clustering yielded 11 species clusters and the provided query strains were assigned to 1 of these. Moreover, user strains were located in 1 of 11 subspecies clusters.

Figure caption genome tree

Figure 2. Tree inferred with FastME 2.1.6.1 [4] from GBDP distances calculated from genome sequences. The branch lengths are scaled in terms of GBDP distance formula d_5. The numbers above branches are GBDP pseudo-bootstrap support values > 60 % from 100 replications, with an average branch support of 62.5 %. The tree was rooted at the midpoint [5].

References

[1] Meier-Kolthoff JP, Göker M. TYGS is an automated high-throughput platform for state-of-the-art genome-based taxonomy. Nat. Commun. 2019;10: 2182. DOI: 10.1038/s41467-019-10210-3

[2] Meier-Kolthoff JP, Sardà Carbasse J, Peinado-Olarte RL, Göker M. TYGS and LPSN: a database tandem for fast and reliable genome-based classification and nomenclature of prokaryotes. Nucleic Acid Res. 2022;50: D801–D807. DOI: 10.1093/nar/gkab902

[3] Meier-Kolthoff JP, Auch AF, Klenk H-P, Göker M. Genome sequence-based species delimitation with confidence intervals and improved distance functions. BMC Bioinformatics. 2013;14: 60. DOI: 10.1186/1471-2105-14-60

[4] Lefort V, Desper R, Gascuel O. FastME 2.0: A comprehensive, accurate, and fast distance-based phylogeny inference program. Mol Biol Evol. 2015;32: 2798–2800. DOI: 10.1093/molbev/msv150

[5] Farris JS. Estimating phylogenetic trees from distance matrices. Am Nat. 1972;106: 645–667.

[6] Kreft L, Botzki A, Coppens F, Vandepoele K, Van Bel M. PhyD3: A phylogenetic tree viewer with extended phyloXML support for functional genomics data visualization. Bioinformatics. 2017;33: 2946–2947. DOI: 10.1093/bioinformatics/btx324

[7] Meier-Kolthoff JP, Hahnke RL, Petersen J, Scheuner C, Michael V, Fiebig A, et al. Complete genome sequence of DSM 30083^T, the type strain (U5/41^T) of *Escherichia coli*, and a proposal for delineating subspecies in microbial taxonomy. Stand Genomic Sci. 2014;9: 2. DOI: 10.1186/1944-3277-9-2
