# Supplementary material for: Tracing active members in microbial communities by BONCAT and click chemistry-based enrichment of newly synthesized proteins
Source: ISME Commun. 2024 Dec 4;4(1):ycae153. doi: 10.1093/ismeco/ycae153 (PMC11683836; doi:10.1093/ismeco/ycae153)
Supplement: Genome_Server_ycae153 [file genome_server_ycae153.zip › Genome Server/Bin_36_TYGS_job_results.pdf]

TYPE (STRAIN) GENOME SERVER

JOB SUMMARY

PRINT DATE:	2024-06-17 09:05:29 +0200
JOB ID:	8ce6b427-b617-4191-b16f-6815bae704ea--12
RESULT PAGE:	https://tygs.dsmz.de/user_results/show?guid=8ce6b427-b617-4191-b16f-6815bae704ea--12

Table 1: Phylogenies

Publication-ready versions of both the genome-scale GBDP tree and the 16S rRNA gene sequence tree can be customized and exported either in SVG (vector graphic) or PNG format from within the phylogeny viewers in your TYGS result page. For publications the **SVG format is recommended** because it is lossless, always keeps its high resolution and can also be easily converted to other popular formats such as PDF or EPS. Please follow the link provided above!

Table 2: Identification

The below list contains the result of the TYGS species identification routine.

Explanation of remarks that might occur in the below table:

remark [R1]: The TYGS type strain database is automatically updated on an almost daily basis. However, if a particular type strain genome is not available in the TYGS database, this can have several reasons which are detailed in the FAQ. You can request an extended 16S rRNA gene analysis via the 16S tree viewer found in your result page to detect **not yet genome-sequenced** type strains relevant for your study.

remark [R2]: > 70% dDDH value (formula d_4) and (almost) minimal dDDH values for gene-content formulae d_0 and d_6 indicate a potentially unreliable identification result and should thus be checked via the 16S rRNA gene sequence similarity. Such strong deviations can, in principle, be caused by sequence contamination.

remark [R3]: G+C content difference of > 1 % indicates a potentially unreliable identification result because within species G+C content varies no more than 1 %, if computed from genome sequences (PMID: 24505073).

Strain	Conclusion	Identification result	Remark
**'bin.36'**	potential new species		see [R1]

Table 3: Pairwise comparisons of user genomes vs. type-strain genomes

The following table contains the pairwise dDDH values between your user genomes and the selected type-strain genomes. The dDDH values are provided along with their confidence intervals (C.I.) for the three different GBDP formulas:

- formula d_0 (a.k.a. GGDC formula 1): length of all HSPs divided by total genome length
- formula d_4 (a.k.a. GGDC formula 2): sum of all identities found in HSPs divided by overall HSP length
- formula d_6 (a.k.a. GGDC formula 3): sum of all identities found in HSPs divided by total genome length

Note: Formula d_4 is independent of genome length and is thus robust against the use of incomplete draft genomes. For other reasons for preferring formula d_4, see the FAQ.

Query	Subject	d_0	C.I. d_0	d_4	C.I. d_4	d_6	C.I. d_6	Diff. G+C Percent
**'bin.36.fa'**	*Alistipes finegoldii* DSM 17242	12.5	[9.8 - 15.7]	37.1	[34.7 - 39.6]	12.9	[10.6 - 15.6]	7.18
**'bin.36.fa'**	*Candidatus* Cryptobacteroides avistercoris B3-1481	12.5	[9.8 - 15.7]	21.0	[18.8 - 23.5]	12.9	[10.6 - 15.6]	9.8
**'bin.36.fa'**	*Propioniciclava sinopodophylli* KCTC 33808	12.5	[9.8 - 15.7]	15.4	[13.4 - 17.7]	12.9	[10.6 - 15.6]	21.36
**'bin.36.fa'**	*Brevundimonas pondensis* LVF1	12.5	[9.8 - 15.7]	15.3	[13.3 - 17.6]	12.9	[10.6 - 15.6]	17.57
**'bin.36.fa'**	*Candidatus* Alistipes stercoravium ChiHjej8B7-9257	12.5	[9.8 - 15.8]	14.1	[12.1 - 16.3]	12.9	[10.6 - 15.7]	11.87
**'bin.36.fa'**	*Lysobacter arseniciresistens* ZS79	12.5	[9.8 - 15.7]	3.7	[2.8 - 4.8]	12.9	[10.6 - 15.6]	20.1
**'bin.36.fa'**	*Bifidobacterium olomucense* DSM 109959	12.5	[9.8 - 15.7]	3.7	[2.8 - 4.8]	12.9	[10.6 - 15.6]	9.47
**'bin.36.fa'**	*Roseivivax marinus* DSM 27511	12.5	[9.8 - 15.7]	3.7	[2.8 - 4.8]	12.9	[10.6 - 15.6]	18.22
**'bin.36.fa'**	*Acidaminococcus fermentans* DSM 20731	12.5	[9.8 - 15.7]	3.7	[2.8 - 4.8]	12.9	[10.6 - 15.6]	6.38
**'bin.36.fa'**	*Geobacter lovleyi* SZ	12.5	[9.8 - 15.7]	3.7	[2.8 - 4.8]	12.9	[10.6 - 15.6]	5.27

Table 4: Strains in your dataset

Joint dataset of automatically determined closest type strains (if this mode was chosen), manually selected type strains (if selected accordingly) and the provided user strains, if provided (marked in yellow).

Strain	Authority	Other deposits	Synonyms	Base pairs	Percent G+C	No. proteins	Goldstamp	Bioproject accession	Biosample accession	Assembly accession	IMG OID
Brevundimonas pondensis LVF1	Friedrich et al. 2021	LMG 32096; CCUG 74982; DSM 112304	*Brevundimonas pondensis*	3550773	67.0	3360		PRJNA664909	SAMN16237053	GCA_017487345	
Candidatus Alistipes stercoravium ChiHjej8B7-9257	Gilroy et al. 2021		*Candidatus* Alistipes stercoravium	2117308	61.3	1758		PRJNA543206	SAMN15816640	GCA_019119615	
Candidatus Cryptobacteroides avistercoris B3-1481	Gilroy et al. 2021		*Candidatus* Cryptobacteroides avistercoris	1642025	59.3	1436		PRJNA543206	SAMN15817057	GCA_017695095	
Propioniciclava sinopodophylli KCTC 33808	Zhang et al. 2017	CCTCC AB 2015257; TEYR-7	*Brevilactibacter sinopodophylli*; *Propioniciclava sinopodophylli*	3066433	70.8	2783		PRJNA517058	SAMN10819038	GCA_004324755	
Bifidobacterium olomucense DSM 109959	Neuzil-Bunesova et al. 2021	CCUG 73845; VB26	*Bifidobacterium olomucense*	2972305	58.9	2372		PRJNA603912	SAMN13944614	GCA_012932375	
Alistipes finegoldii DSM 17242	Rautio et al. 2003	AHN 2437; CIP 107999; CCUG 46020; JCM 16770	*Alistipes finegoldii*	3734239	56.6	3110	Gp0001228	PRJNA40775	SAMN02232007	GCA_000265365	2509601035
Acidaminococcus fermentans DSM 20731	Rogosa 1969 emend. Cook et al. 1994	CIP 106432; ATCC 25085; CCUG 9996; VR4	*Acidaminococcus fermentans*	2329769	55.8	2026	Gp0001186	PRJNA33685	SAMN02598507	GCA_000025305	646311901
Lysobacter arseniciresistens ZS79	Luo et al. 2012	CGMCC 1.10752; KCTC 23365; DSM 27222	*Lysobacter arseniciresistens*	3085112	69.6	2363	Gp0048195	PRJNA214588	SAMN03098276	GCA_000768335	

Strain	Authority	Other deposits	Synonyms	Base pairs	Percent G+C	No. proteins	Goldstamp	Bioproject accession	Biosample accession	Assembly accession	IMG OID
Roseivivax marinus DSM 27511	Dai et al. 2014	KCTC 32470; JCM 19386; ZL136	*Roseivivax marinus*	4013 095	67.7	3746	Gp0148843	PRJNA331380	SAMN05444413	GCA_900109705	2693429879
Geobacter lovleyi SZ	Sung et al. 2009	ATCC BAA-1151; DSM 17278	*Geobacter lovleyi*; *Trichlorobacter lovleyi*	3994 874	54.7	3685	Gp0002255	PRJNA17423	SAMN00623044	GCA_000020385	642555130
bin.36.fa				2240 816	49.5	1991					

Methods, Results and References

The genome sequence data were uploaded to the Type (Strain) Genome Server (TYGS), a free bioinformatics platform available under https://tygs.dsmz.de, for a whole genome-based taxonomic analysis [1]. The analysis also made use of recently introduced methodological updates and features [2]. Information on nomenclature, synonymy and associated taxonomic literature was provided by TYGS's sister database, the List of Prokaryotic names with Standing in Nomenclature (LPSN, available at https://lpsn.dsmz.de) [2]. The results were provided by the TYGS on 2024-06-16. The TYGS analysis was subdivided into the following steps:

Determination of closely related type strains

The determination of closely related type strains did not succeed because not a single 16S rDNA gene sequence was detected in the provided user genomes. The subsequent analyses are thus only based on the provided genome data and the manually selected type strains, if any.

Pairwise comparison of genome sequences

For the phylogenomic inference, all pairwise comparisons among the set of genomes were conducted using GBDP and accurate intergenomic distances inferred under the algorithm 'trimming' and distance formula d_5 [3]. 100 distance replicates were calculated each. Digital DDH values and confidence intervals were calculated using the recommended settings of the GGDC 4.0 [2,3].

Phylogenetic inference

The resulting intergenomic distances were used to infer a balanced minimum evolution tree with branch support via FASTME 2.1.6.1 including SPR postprocessing [4]. Branch support was inferred from 100 pseudo-bootstrap replicates each. The trees were rooted at the midpoint [5] and visualized with PhyD3 [6].

Type-based species and subspecies clustering

The type-based species clustering using a 70% dDDH radius around each of the 10 type strains was done as previously described [1]. The resulting groups are shown in Table 1 and 4. Subspecies clustering was done using a 79% dDDH threshold as previously introduced [7].

Results

Type-based species and subspecies clustering

The resulting species and subspecies clusters are listed in Table 4, whereas the taxonomic identification of the query strains is found in Table 1. Briefly, the clustering yielded 11 species clusters and the provided query strains were assigned to 1 of these. Moreover, user strains were located in 1 of 11 subspecies clusters.

Figure caption genome tree

Figure 2. Tree inferred with FastME 2.1.6.1 [4] from GBDP distances calculated from genome sequences. The branch lengths are scaled in terms of GBDP distance formula d_5. The numbers above branches are GBDP pseudo-bootstrap support values > 60 % from 100 replications, with an average branch support of 76.0 %. The tree was rooted at the midpoint [5].

References

[1] Meier-Kolthoff JP, Göker M. TYGS is an automated high-throughput platform for state-of-the-art genome-based taxonomy. Nat. Commun. 2019;10: 2182. DOI: 10.1038/s41467-019-10210-3

[2] Meier-Kolthoff JP, Sardà Carbasse J, Peinado-Olarte RL, Göker M. TYGS and LPSN: a database tandem for fast and reliable genome-based classification and nomenclature of prokaryotes. Nucleic Acid Res. 2022;50: D801–D807. DOI: 10.1093/nar/gkab902

[3] Meier-Kolthoff JP, Auch AF, Klenk H-P, Göker M. Genome sequence-based species delimitation with confidence intervals and improved distance functions. BMC Bioinformatics. 2013;14: 60. DOI: 10.1186/1471-2105-14-60

[4] Lefort V, Desper R, Gascuel O. FastME 2.0: A comprehensive, accurate, and fast distance-based phylogeny inference program. Mol Biol Evol. 2015;32: 2798–2800. DOI: 10.1093/molbev/msv150

[5] Farris JS. Estimating phylogenetic trees from distance matrices. Am Nat. 1972;106: 645–667.

[6] Kreft L, Botzki A, Coppens F, Vandepoele K, Van Bel M. PhyD3: A phylogenetic tree viewer with extended phyloXML support for functional genomics data visualization. Bioinformatics. 2017;33: 2946–2947. DOI: 10.1093/bioinformatics/btx324

[7] Meier-Kolthoff JP, Hahnke RL, Petersen J, Scheuner C, Michael V, Fiebig A, et al. Complete genome sequence of DSM 30083^{T}, the type strain (U5/41^{T}) of *Escherichia coli*, and a proposal for delineating subspecies in microbial taxonomy. Stand Genomic Sci. 2014;9: 2. DOI: 10.1186/1944-3277-9-2
