# Supplementary material for: Tracing active members in microbial communities by BONCAT and click chemistry-based enrichment of newly synthesized proteins
Source: ISME Commun. 2024 Dec 4;4(1):ycae153. doi: 10.1093/ismeco/ycae153 (PMC11683836; doi:10.1093/ismeco/ycae153)
Supplement: Genome_Server_ycae153 [file genome_server_ycae153.zip › Genome Server/Bin_40_TYGS_job_results.pdf]

TYPE (STRAIN) GENOME SERVER

JOB SUMMARY

PRINT DATE:	2024-06-17 09:13:34 +0200
JOB ID:	8ce6b427-b617-4191-b16f-6815bae704ea--13
RESULT PAGE:	https://tygs.dsmz.de/user_results/show?guid=8ce6b427-b617-4191-b16f-6815bae704ea--13

Table 1: Phylogenies

Publication-ready versions of both the genome-scale GBDP tree and the 16S rRNA gene sequence tree can be customized and exported either in SVG (vector graphic) or PNG format from within the phylogeny viewers in your TYGS result page. For publications the **SVG format is recommended** because it is lossless, always keeps its high resolution and can also be easily converted to other popular formats such as PDF or EPS. Please follow the link provided above!

Table 2: Identification

The below list contains the result of the TYGS species identification routine.

Explanation of remarks that might occur in the below table:

remark [R1]: The TYGS type strain database is automatically updated on an almost daily basis. However, if a particular type strain genome is not available in the TYGS database, this can have several reasons which are detailed in the FAQ. You can request an extended 16S rRNA gene analysis via the 16S tree viewer found in your result page to detect **not yet genome-sequenced** type strains relevant for your study.

remark [R2]: > 70% dDDH value (formula d_4) and (almost) minimal dDDH values for gene-content formulae d_0 and d_6 indicate a potentially unreliable identification result and should thus be checked via the 16S rRNA gene sequence similarity. Such strong deviations can, in principle, be caused by sequence contamination.

remark [R3]: G+C content difference of > 1 % indicates a potentially unreliable identification result because within species G+C content varies no more than 1 %, if computed from genome sequences (PMID: 24505073).

Strain	Conclusion	Identification result	Remark
**'bin.40'**	potential new species		see [R1]

Table 3: Pairwise comparisons of user genomes vs. type-strain genomes

The following table contains the pairwise dDDH values between your user genomes and the selected type-strain genomes. The dDDH values are provided along with their confidence intervals (C.I.) for the three different GBDP formulas:

- formula d_0 (a.k.a. GGDC formula 1): length of all HSPs divided by total genome length
- formula d_4 (a.k.a. GGDC formula 2): sum of all identities found in HSPs divided by overall HSP length
- formula d_6 (a.k.a. GGDC formula 3): sum of all identities found in HSPs divided by total genome length

Note: Formula d_4 is independent of genome length and is thus robust against the use of incomplete draft genomes. For other reasons for preferring formula d_4, see the FAQ.

Query	Subject	d_0	C.I. d_0	d_4	C.I. d_4	d_6	C.I. d_6	Diff. G+C Percent
**'bin.40.fa'**	*Atribacter laminatus* RT761T	17.9	[14.8 - 21.4]	34.9	[32.5 - 37.4]	18.3	[15.6 - 21.3]	0.53
**'bin.40.fa'**	*Usitatibacter rugosus* DSM 104443T	12.5	[9.8 - 15.7]	3.7	[2.8 - 4.8]	12.9	[10.6 - 15.6]	28.86
**'bin.40.fa'**	*Paenibacillus prosopidis* CECT7506	12.5	[9.8 - 15.7]	3.7	[2.8 - 4.8]	12.9	[10.6 - 15.6]	8.86
**'bin.40.fa'**	*Candidatus* Enterousia avistercoris 8207	12.5	[9.8 - 15.7]	3.7	[2.8 - 4.8]	12.9	[10.6 - 15.6]	5.63
**'bin.40.fa'**	*Clostridium cadaveris* JCM 1392	12.5	[9.8 - 15.7]	3.7	[2.8 - 4.8]	12.9	[10.6 - 15.6]	7.11
**'bin.40.fa'**	*Clostridium polynesiense* MS1 T	12.5	[9.8 - 15.7]	3.7	[2.8 - 4.8]	12.9	[10.6 - 15.6]	4.18
**'bin.40.fa'**	*Chitinimonas taiwanensis* DSM 18899	12.5	[9.8 - 15.7]	3.7	[2.8 - 4.8]	12.9	[10.6 - 15.6]	24.71
**'bin.40.fa'**	*Solitalea canadensis* DSM 3403	12.5	[9.8 - 15.7]	3.7	[2.8 - 4.8]	12.9	[10.6 - 15.6]	0.85
**'bin.40.fa'**	*Methanobacterium bryantii* M. o. H.	12.5	[9.8 - 15.7]	3.7	[2.8 - 4.8]	12.9	[10.6 - 15.6]	4.97
**'bin.40.fa'**	*Alkaliphilus oremlandii* OhILAs	12.5	[9.8 - 15.7]	3.7	[2.8 - 4.8]	12.9	[10.6 - 15.6]	1.9

Table 4: Strains in your dataset

Joint dataset of automatically determined closest type strains (if this mode was chosen), manually selected type strains (if selected accordingly) and the provided user strains, if provided (marked in yellow).

Strain	Authority	Other deposits	Synonyms	Base pairs	Percent G+C	No. proteins	Goldstamp	Bioproject accession	Biosample accession	Assembly accession	IMG OID
Paenibacillus prosopidis CECT7506	Valverde et al. 2010	LMG 25259; DSM 22405; PW21	*Paenibacillus prosopidis*	6628600	47.0	6052	Gp0290074	PRJNA463302	SAMN09074970	GCA_003337375	
Usitatibacter rugosus DSM 104443T	Vieira et al. 2021	0125_3; LMG 29998; CECT 9241	*Usitatibacter rugosus*	4563145	67.0	4259		PRJNA224116	SAMN14766570	GCF_013003965	
Candidatus Enterousia avistercoris 8207	Gilroy et al. 2021		*Candidatus* Enterousia avistercoris	820108	43.8	835		PRJNA543206	SAMN15817150	GCA_017694255	
Clostridium cadaveris JCM 1392	(Klein 1899) McClung and McCoy 1957	CIP 104314; ATCC 25783; CCUG 24035; DSM 1284	*Bacillus cadaveris*; *Clostridium cadaveris*	3388931	31.0	3215		PRJDB10510	SAMD00644925	GCA_039521325	
Chitinimonas taiwanensis DSM 18899	Chang et al. 2004	LMG 22011; BCRC 17210; BCRC (formerly CCRC) 17210; CCRC 17210; cf	*Chitinimonas taiwanensis*	4074648	62.9	3700	Gp0013423	PRJNA220995	SAMN02745887	GCA_900119825	2599185263
Solitalea canadensis DSM 3403	(Christensen 1980) Weon et al. 2009	LMG 8368; CIP 104802; ATCC 29591; JCM 21819; IFO 15130; NBRC 15130; KACC 13276; UASM 9D	*Flexibacter canadensis*; *Solitalea canadensis*	5202069	37.3	4310	Gp0004685	PRJNA60421	SAMN02232052	GCA_000242635	2509276062

Strain	Authority	Other deposits	Synonyms	Base pairs	Percent G+C	No. proteins	Goldstamp	Bioproject accession	Biosample accession	Assembly accession	IMG OID
Methanobacterium bryantii M. o. H.	Balch and Wolfe 1981 emend. Judicial Commission 1992	ATCC 33272; DSM 863; VKM B-1629; OCM 110	*Methanobacterium bryantii*	3466 370	33.2	3168	Gp0322642	PRJNA300714	SAMN04229035	GCA_002287175	
Alkaliphilus oremlandii OhILAs	Fisher et al. 2009	ATCC BAA-1360; DSM 21761	*Alkaliphilus oremlandii*	3123 558	36.3	2836	Gp0000034	PRJNA16083	SAMN00623039	GCA_000018325	641228475
Clostridium polynesiense MS1 T	Sankar et al. 2023	DSM 27072; CSUR P630	*Clostridium polynesiense*	3552 641	34.0	3456		PRJNA224116	SAMEA2769924	GCF_000820705	
Atribacter laminatus RT761T	Katayama et al. 2021	DSM 105538; NBRC 112890	*Atribacter laminatus*	3101 225	38.7	2759		PRJNA528842	SAMN11246273	GCA_015775515	
bin.40.fa				4750 55	38.2	528					

Methods, Results and References

The genome sequence data were uploaded to the Type (Strain) Genome Server (TYGS), a free bioinformatics platform available under https://tygs.dsmz.de, for a whole genome-based taxonomic analysis [1]. The analysis also made use of recently introduced methodological updates and features [2]. Information on nomenclature, synonymy and associated taxonomic literature was provided by TYGS's sister database, the List of Prokaryotic names with Standing in Nomenclature (LPSN, available at https://lpsn.dsmz.de) [2]. The results were provided by the TYGS on 2024-06-16. The TYGS analysis was subdivided into the following steps:

Determination of closely related type strains

The determination of closely related type strains did not succeed because not a single 16S rDNA gene sequence was detected in the provided user genomes. The subsequent analyses are thus only based on the provided genome data and the manually selected type strains, if any.

Pairwise comparison of genome sequences

For the phylogenomic inference, all pairwise comparisons among the set of genomes were conducted using GBDP and accurate intergenomic distances inferred under the algorithm 'trimming' and distance formula d_5 [3]. 100 distance replicates were calculated each. Digital DDH values and confidence intervals were calculated using the recommended settings of the GGDC 4.0 [2,3].

Phylogenetic inference

The resulting intergenomic distances were used to infer a balanced minimum evolution tree with branch support via FASTME 2.1.6.1 including SPR postprocessing [4]. Branch support was inferred from 100 pseudo-bootstrap replicates each. The trees were rooted at the midpoint [5] and visualized with PhyD3 [6].

Type-based species and subspecies clustering

The type-based species clustering using a 70% dDDH radius around each of the 10 type strains was done as previously described [1]. The resulting groups are shown in Table 1 and 4. Subspecies clustering was done using a 79% dDDH threshold as previously introduced [7].

Results

Type-based species and subspecies clustering

The resulting species and subspecies clusters are listed in Table 4, whereas the taxonomic identification of the query strains is found in Table 1. Briefly, the clustering yielded 11 species clusters and the provided query strains were assigned to 1 of these. Moreover, user strains were located in 1 of 11 subspecies clusters.

Figure caption genome tree

Figure 2. Tree inferred with FastME 2.1.6.1 [4] from GBDP distances calculated from genome sequences. The branch lengths are scaled in terms of GBDP distance formula d_5. The numbers above branches are GBDP pseudo-bootstrap support values > 60 % from 100 replications, with an average branch support of 77.8 %. The tree was rooted at the midpoint [5].

References

[1] Meier-Kolthoff JP, Göker M. TYGS is an automated high-throughput platform for state-of-the-art genome-based taxonomy. Nat. Commun. 2019;10: 2182. DOI: 10.1038/s41467-019-10210-3

[2] Meier-Kolthoff JP, Sardà Carbasse J, Peinado-Olarte RL, Göker M. TYGS and LPSN: a database tandem for fast and reliable genome-based classification and nomenclature of prokaryotes. Nucleic Acid Res. 2022;50: D801–D807. DOI: 10.1093/nar/gkab902

[3] Meier-Kolthoff JP, Auch AF, Klenk H-P, Göker M. Genome sequence-based species delimitation with confidence intervals and improved distance functions. BMC Bioinformatics. 2013;14: 60. DOI: 10.1186/1471-2105-14-60

[4] Lefort V, Desper R, Gascuel O. FastME 2.0: A comprehensive, accurate, and fast distance-based phylogeny inference program. Mol Biol Evol. 2015;32: 2798–2800. DOI: 10.1093/molbev/msv150

[5] Farris JS. Estimating phylogenetic trees from distance matrices. Am Nat. 1972;106: 645–667.

[6] Kreft L, Botzki A, Coppens F, Vandepoele K, Van Bel M. PhyD3: A phylogenetic tree viewer with extended phyloXML support for functional genomics data visualization. Bioinformatics. 2017;33: 2946–2947. DOI: 10.1093/bioinformatics/btx324

[7] Meier-Kolthoff JP, Hahnke RL, Petersen J, Scheuner C, Michael V, Fiebig A, et al. Complete genome sequence of DSM 30083^T, the type strain (U5/41^T) of *Escherichia coli*, and a proposal for delineating subspecies in microbial taxonomy. Stand Genomic Sci. 2014;9: 2. DOI: 10.1186/1944-3277-9-2
