# Supplementary material for: Tracing active members in microbial communities by BONCAT and click chemistry-based enrichment of newly synthesized proteins
Source: ISME Commun. 2024 Dec 4;4(1):ycae153. doi: 10.1093/ismeco/ycae153 (PMC11683836; doi:10.1093/ismeco/ycae153)
Supplement: Genome_Server_ycae153 [file genome_server_ycae153.zip › Genome Server/Bin_41_TYGS_job_results.pdf]

TYPE (STRAIN) GENOME SERVER

JOB SUMMARY

PRINT DATE:	2024-06-17 09:07:55 +0200
JOB ID:	8ce6b427-b617-4191-b16f-6815bae704ea--14
RESULT PAGE:	https://tygs.dsmz.de/user_results/show?guid=8ce6b427-b617-4191-b16f-6815bae704ea--14

Table 1: Phylogenies

Publication-ready versions of both the genome-scale GBDP tree and the 16S rRNA gene sequence tree can be customized and exported either in SVG (vector graphic) or PNG format from within the phylogeny viewers in your TYGS result page. For publications the **SVG format is recommended** because it is lossless, always keeps its high resolution and can also be easily converted to other popular formats such as PDF or EPS. Please follow the link provided above!

Table 2: Identification

The below list contains the result of the TYGS species identification routine.

Explanation of remarks that might occur in the below table:

remark [R1]: The TYGS type strain database is automatically updated on an almost daily basis. However, if a particular type strain genome is not available in the TYGS database, this can have several reasons which are detailed in the FAQ. You can request an extended 16S rRNA gene analysis via the 16S tree viewer found in your result page to detect **not yet genome-sequenced** type strains relevant for your study.

remark [R2]: > 70% dDDH value (formula d_4) and (almost) minimal dDDH values for gene-content formulae d_0 and d_6 indicate a potentially unreliable identification result and should thus be checked via the 16S rRNA gene sequence similarity. Such strong deviations can, in principle, be caused by sequence contamination.

remark [R3]: G+C content difference of > 1 % indicates a potentially unreliable identification result because within species G+C content varies no more than 1 %, if computed from genome sequences (PMID: 24505073).

Strain	Conclusion	Identification result	Remark
**'bin.41'**	potential new species		see [R1]

Table 3: Pairwise comparisons of user genomes vs. type-strain genomes

The following table contains the pairwise dDDH values between your user genomes and the selected type-strain genomes. The dDDH values are provided along with their confidence intervals (C.I.) for the three different GBDP formulas:

- formula d_0 (a.k.a. GGDC formula 1): length of all HSPs divided by total genome length
- formula d_4 (a.k.a. GGDC formula 2): sum of all identities found in HSPs divided by overall HSP length
- formula d_6 (a.k.a. GGDC formula 3): sum of all identities found in HSPs divided by total genome length

Note: Formula d_4 is independent of genome length and is thus robust against the use of incomplete draft genomes. For other reasons for preferring formula d_4, see the FAQ.

Query	Subject	d_0	C.I. d_0	d_4	C.I. d_4	d_6	C.I. d_6	Diff. G+C Percent
**'bin.41.fa'**	*Zhaonella formicivorans* k32	12.5	[9.8 - 15.7]	29.3	[26.9 - 31.8]	12.9	[10.6 - 15.6]	4.78
**'bin.41.fa'**	*Candidatus* Evtepia excrementipullorum ChiSjej3B21-3892	12.5	[9.9 - 15.8]	19.4	[17.2 - 21.8]	12.9	[10.6 - 15.7]	12.27
**'bin.41.fa'**	*Pelotomaculum thermopropionicum* SI	13.2	[10.4 - 16.5]	18.8	[16.6 - 21.2]	13.5	[11.1 - 16.3]	2.62
**'bin.41.fa'**	*Candidatus* Oscillibacter avistercoris CHK176-14096	12.5	[9.8 - 15.8]	18.4	[16.3 - 20.8]	12.9	[10.6 - 15.6]	13.42
**'bin.41.fa'**	*Variovorax defluvii* JCM 17804	12.5	[9.8 - 15.7]	3.7	[2.8 - 4.8]	12.9	[10.6 - 15.6]	17.45
**'bin.41.fa'**	*Chryseobacterium hominis* DSM 22165	12.5	[9.8 - 15.7]	3.7	[2.8 - 4.8]	12.9	[10.6 - 15.6]	14.74
**'bin.41.fa'**	*Thermanaerovibrio acidaminovorans* DSM 6589	12.5	[9.8 - 15.7]	3.7	[2.8 - 4.8]	12.9	[10.6 - 15.6]	13.45
**'bin.41.fa'**	*Inhella proteolytica* 1Y17	12.5	[9.8 - 15.7]	3.7	[2.8 - 4.8]	12.9	[10.6 - 15.6]	18.96
**'bin.41.fa'**	*Pseudomonas shahriarae* SWRI52	12.5	[9.8 - 15.7]	3.7	[2.8 - 4.8]	12.9	[10.6 - 15.6]	10.25
**'bin.41.fa'**	*Nocardioides sediminis* KCTC 19271	12.5	[9.8 - 15.7]	3.7	[2.8 - 4.8]	12.9	[10.6 - 15.6]	21.97

Table 4: Strains in your dataset

Joint dataset of automatically determined closest type strains (if this mode was chosen), manually selected type strains (if selected accordingly) and the provided user strains, if provided (marked in yellow).

Strain	Authority	Other deposits	Synonyms	Base pairs	Percent G+C	No. proteins	Goldstamp	Bioproject accession	Biosample accession	Assembly accession	IMG OID
Zhaonella formicivorans k32	Lv et al. 2020	CGMCC 1.5297; DSM 107278; CCAM 584	*Zhaonella formicivorans*	3314 304	45.6	3225		PRJNA523471	SAMN10977731	GCA_004353525	
Inhella proteolytica 1Y17	Liu et al. 2021	GDMCC 1.1830; KACC 21948	*Inhella proteolytica*	4963 995	69.3	4447		PRJNA224116	SAMN17013839	GCF_016093275	
Candidatus Oscillibacter avistercoris CHK176-14096	Gilroy et al. 2021		*Candidatus* Oscillibacter avistercoris	2144 148	63.8	2109		PRJNA543206	SAMN15816820	GCA_019119105	
Candidatus Evtepia excrementipullorum ChiSjej3B21-3892	Gilroy et al. 2021		*Candidatus* Evtepia excrementipullorum	2108 333	62.6	2079		PRJNA543206	SAMN15816827	GCA_019118615	
Pseudomonas shahriarae SWRI52	Girard et al. 2022	CFBP 8859; LMG 32046	*Pseudomonas shahriarae*	6054 205	60.6	5318		PRJNA639797	SAMN15248350	GCA_014268455	
Chryseobacterium hominis DSM 22165	Vaneechoutte et al. 2007	CIP 109415; CCUG 52711; NF802	*Chryseobacterium hominis*; *Epilithonimonas hominis*	2913 671	35.6	2681	Gp0443797	PRJNA498692	SAMN01174621	GCA_003730015	
Pelotomaculum thermopropionicum SI	Imachi et al. 2002	DSM 13744; JCM 10971	*Pelotomaculum thermopropionicum*	3025 375	53.0	2920	Gp0000155	PRJDA19023	SAMD00060921	GCA_000010565	640427128
Nocardioides sediminis KCTC 19271	Dastager et al. 2009	DSM 19263; MSL-01	*Nocardioides sediminis*	4284 245	72.3	4124		PRJNA448055	SAMN08820071	GCA_003047295	
Variovorax defluvii JCM 17804	Jin et al. 2012	2C1-b; KCTC 12768; DSM 27259	*Variovorax defluvii*	6001 042	67.8	5626		PRJDB10510	SAMD00645733	GCA_039541515	

Strain	Authority	Other deposits	Synonyms	Base pairs	Percent G+C	No. proteins	Goldstamp	Bioproject accession	Biosample accession	Assembly accession	IMG OID
Thermanaerovibrio acidaminovorans DSM 6589	(Guangsheng et al. 1997) Baena et al. 1999 emend. Palaniappan et al. 2013	ATCC 49978; Su883	*Selenomonas acidaminovorans*; *Thermanaerovibrio acidaminovorans*	1848474	63.8	1738	Gp0001224	PRJNA29531	SAMN00002595	GCA_000024905	646311961
bin.41.fa				2310074	50.3	2331					

Methods, Results and References

The genome sequence data were uploaded to the Type (Strain) Genome Server (TYGS), a free bioinformatics platform available under https://tygs.dsmz.de, for a whole genome-based taxonomic analysis [1]. The analysis also made use of recently introduced methodological updates and features [2]. Information on nomenclature, synonymy and associated taxonomic literature was provided by TYGS's sister database, the List of Prokaryotic names with Standing in Nomenclature (LPSN, available at https://lpsn.dsmz.de) [2]. The results were provided by the TYGS on 2024-06-16. The TYGS analysis was subdivided into the following steps:

Determination of closely related type strains

The determination of closely related type strains did not succeed because not a single 16S rDNA gene sequence was detected in the provided user genomes. The subsequent analyses are thus only based on the provided genome data and the manually selected type strains, if any.

Pairwise comparison of genome sequences

For the phylogenomic inference, all pairwise comparisons among the set of genomes were conducted using GBDP and accurate intergenomic distances inferred under the algorithm 'trimming' and distance formula d_5 [3]. 100 distance replicates were calculated each. Digital DDH values and confidence intervals were calculated using the recommended settings of the GGDC 4.0 [2,3].

Phylogenetic inference

The resulting intergenomic distances were used to infer a balanced minimum evolution tree with branch support via FASTME 2.1.6.1 including SPR postprocessing [4]. Branch support was inferred from 100 pseudo-bootstrap replicates each. The trees were rooted at the midpoint [5] and visualized with PhyD3 [6].

Type-based species and subspecies clustering

The type-based species clustering using a 70% dDDH radius around each of the 10 type strains was done as previously described [1]. The resulting groups are shown in Table 1 and 4. Subspecies clustering was done using a 79% dDDH threshold as previously introduced [7].

Results

Type-based species and subspecies clustering

The resulting species and subspecies clusters are listed in Table 4, whereas the taxonomic identification of the query strains is found in Table 1. Briefly, the clustering yielded 11 species clusters and the provided query strains were assigned to 1 of these. Moreover, user strains were located in 1 of 11 subspecies clusters.

Figure caption genome tree

Figure 2. Tree inferred with FastME 2.1.6.1 [4] from GBDP distances calculated from genome sequences. The branch lengths are scaled in terms of GBDP distance formula d_5. The numbers above branches are GBDP pseudo-bootstrap support values > 60 % from 100 replications, with an average branch support of 88.6 %. The tree was rooted at the midpoint [5].

References

[1] Meier-Kolthoff JP, Göker M. TYGS is an automated high-throughput platform for state-of-the-art genome-based taxonomy. Nat. Commun. 2019;10: 2182. DOI: 10.1038/s41467-019-10210-3

[2] Meier-Kolthoff JP, Sardà Carbasse J, Peinado-Olarte RL, Göker M. TYGS and LPSN: a database tandem for fast and reliable genome-based classification and nomenclature of prokaryotes. Nucleic Acid Res. 2022;50: D801–D807. DOI: 10.1093/nar/gkab902

[3] Meier-Kolthoff JP, Auch AF, Klenk H-P, Göker M. Genome sequence-based species delimitation with confidence intervals and improved distance functions. BMC Bioinformatics. 2013;14: 60. DOI: 10.1186/1471-2105-14-60

[4] Lefort V, Desper R, Gascuel O. FastME 2.0: A comprehensive, accurate, and fast distance-based phylogeny inference program. Mol Biol Evol. 2015;32: 2798–2800. DOI: 10.1093/molbev/msv150

[5] Farris JS. Estimating phylogenetic trees from distance matrices. Am Nat. 1972;106: 645–667.

[6] Kreft L, Botzki A, Coppens F, Vandepoele K, Van Bel M. PhyD3: A phylogenetic tree viewer with extended phyloXML support for functional genomics data visualization. Bioinformatics. 2017;33: 2946–2947. DOI: 10.1093/bioinformatics/btx324

[7] Meier-Kolthoff JP, Hahnke RL, Petersen J, Scheuner C, Michael V, Fiebig A, et al. Complete genome sequence of DSM 30083^T, the type strain (U5/41^T) of *Escherichia coli*, and a proposal for delineating subspecies in microbial taxonomy. Stand Genomic Sci. 2014;9: 2. DOI: 10.1186/1944-3277-9-2
