# Supplementary material for: Tracing active members in microbial communities by BONCAT and click chemistry-based enrichment of newly synthesized proteins
Source: ISME Commun. 2024 Dec 4;4(1):ycae153. doi: 10.1093/ismeco/ycae153 (PMC11683836; doi:10.1093/ismeco/ycae153)
Supplement: Genome_Server_ycae153 [file genome_server_ycae153.zip › Genome Server/Bin_42_TYGS_job_results.pdf]

TYPE (STRAIN) GENOME SERVER

JOB SUMMARY

PRINT DATE:	2024-06-17 09:12:33 +0200
JOB ID:	8ce6b427-b617-4191-b16f-6815bae704ea--15
RESULT PAGE:	https://tygs.dsmz.de/user_results/show?guid=8ce6b427-b617-4191-b16f-6815bae704ea--15

Table 1: Phylogenies

Publication-ready versions of both the genome-scale GBDP tree and the 16S rRNA gene sequence tree can be customized and exported either in SVG (vector graphic) or PNG format from within the phylogeny viewers in your TYGS result page. For publications the **SVG format is recommended** because it is lossless, always keeps its high resolution and can also be easily converted to other popular formats such as PDF or EPS. Please follow the link provided above!

Table 2: Identification

The below list contains the result of the TYGS species identification routine.

Explanation of remarks that might occur in the below table:

remark [R1]: The TYGS type strain database is automatically updated on an almost daily basis. However, if a particular type strain genome is not available in the TYGS database, this can have several reasons which are detailed in the FAQ. You can request an extended 16S rRNA gene analysis via the 16S tree viewer found in your result page to detect **not yet genome-sequenced** type strains relevant for your study.

remark [R2]: > 70% dDDH value (formula d_4) and (almost) minimal dDDH values for gene-content formulae d_0 and d_6 indicate a potentially unreliable identification result and should thus be checked via the 16S rRNA gene sequence similarity. Such strong deviations can, in principle, be caused by sequence contamination.

remark [R3]: G+C content difference of > 1 % indicates a potentially unreliable identification result because within species G+C content varies no more than 1 %, if computed from genome sequences (PMID: 24505073).

Strain	Conclusion	Identification result	Remark
**'bin.42'**	belongs to known species	*Candidatus* Syntrophosphaera thermopropionivorans	

Table 3: Pairwise comparisons of user genomes vs. type-strain genomes

The following table contains the pairwise dDDH values between your user genomes and the selected type-strain genomes. The dDDH values are provided along with their confidence intervals (C.I.) for the three different GBDP formulas:

- formula d_0 (a.k.a. GGDC formula 1): length of all HSPs divided by total genome length
- formula d_4 (a.k.a. GGDC formula 2): sum of all identities found in HSPs divided by overall HSP length
- formula d_6 (a.k.a. GGDC formula 3): sum of all identities found in HSPs divided by total genome length

Note: Formula d_4 is independent of genome length and is thus robust against the use of incomplete draft genomes. For other reasons for preferring formula d_4, see the FAQ.

Query	Subject	d_0	C.I. d_0	d_4	C.I. d_4	d_6	C.I. d_6	Diff. G+C Percent
**'bin.42.fa'**	*Candidatus* Syntrophosphaera thermopropionivorans W5	85.1	[81.3 - 88.2]	78.8	[75.9 - 81.5]	86.9	[83.9 - 89.5]	0.17
**'bin.42.fa'**	*Lactobacillus pasteurii* CRBIP 24.76	12.5	[9.8 - 15.8]	28.9	[26.5 - 31.4]	12.9	[10.6 - 15.6]	2.16
**'bin.42.fa'**	*Candidatus* Syntrophocurvum alkaliphilum B(2M) T	12.5	[9.8 - 15.7]	26.5	[24.2 - 29.0]	12.9	[10.6 - 15.6]	4.0
**'bin.42.fa'**	*Clostridium weizhouense* YB-6	12.5	[9.8 - 15.7]	24.9	[22.6 - 27.4]	12.9	[10.6 - 15.6]	10.01
**'bin.42.fa'**	*Clostridium frigoriphilum* DSM 17811	12.5	[9.8 - 15.7]	21.1	[18.8 - 23.5]	12.9	[10.6 - 15.6]	5.7
**'bin.42.fa'**	*Selenobaculum gibii* ICN-92133	12.5	[9.8 - 15.8]	20.8	[18.6 - 23.2]	12.9	[10.6 - 15.7]	0.98
**'bin.42.fa'**	*Candidatus* Cloacimonas acidaminivorans Evry	12.8	[10.1 - 16.1]	18.8	[16.6 - 21.2]	13.2	[10.8 - 15.9]	1.42
**'bin.42.fa'**	*Mesoplasma chauliocola* ATCC 49578	12.5	[9.8 - 15.7]	3.7	[2.8 - 4.8]	12.9	[10.6 - 15.6]	9.59
**'bin.42.fa'**	*Candidatus* Fimicola merdigallinarum F6-4510	12.5	[9.8 - 15.7]	3.7	[2.8 - 4.8]	12.9	[10.6 - 15.6]	3.99
**'bin.42.fa'**	*Candidatus* Nitrosopumilus koreensis AR1	12.5	[9.8 - 15.7]	3.7	[2.8 - 4.8]	12.9	[10.6 - 15.6]	2.27

Table 4: Strains in your dataset

Joint dataset of automatically determined closest type strains (if this mode was chosen), manually selected type strains (if selected accordingly) and the provided user strains, if provided (marked in yellow).

Strain	Authority	Other deposits	Synonyms	Base pairs	Percent G+C	No. proteins	Goldstamp	Bioproject accession	Biosample accession	Assembly accession	IMG OID
Candidatus Syntrophocurvum alkaliphilum B(2M) T	Sorokin et al. 2016	UNIQEMU998	*Candidatus* Syntrophocurvum alkaliphilum	2360781	32.5	2301		PRJNA592435	SAMN13426661	GCA_009734445	
Clostridium frigoriphilum DSM 17811	Dorn-In et al. 2018	14F	*Clostridium frigoriphilum*	5099045	30.7	4783		PRJNA1041631	SAMN38289517	GCA_036541405	
Candidatus Fimicola merdigallinarum F6-4510	Gilroy et al. 2021		*Candidatus* Fimicola merdigallinarum	1804002	32.5	1666		PRJNA543206	SAMN15817136	GCA_017694425	
Clostridium weizhouense YB-6	Li et al. 2022	JCM 34754; GDMCC 1.2529	*Clostridium weizhouense*	3955783	26.4	3500		PRJNA746791	SAMN20295133	GCA_019431045	
Mesoplasma chauliocola ATCC 49578	Tully et al. 1994	CHPA-2	*Entomoplasma chauliocola*; *Mesoplasma chauliocola*	842399	26.9	742	Gp0018898	PRJNA204049	SAMN02584961	GCA_000518825	2545555831
Candidatus Cloacimonas acidaminivorans Evry	Pelletier et al. 2008		*Candidatus* Cloacimonas acidaminivorans	2246820	37.9	1725		PRJNA224116	SAMEA2272155	GCF_000146065	
Candidatus Nitrosopumilus koreensis AR1	Kim et al. 2011		*Candidatus* Nitrosopumilus koreensis	1639964	34.2	1890	Gp0025903	PRJNA174387	SAMN02603137	GCA_000299365	2518645532
Lactobacillus pasteurii CRBIP 24.76	Cousin et al. 2013	1517; DSM 23907	*Lactobacillus pasteurii*	1905398	38.6	1845	Gp0035270	PRJEB103	SAMEA2272206	GCA_000297025	2563366755
Candidatus Syntrophosphaera thermopropionivorans W5	Dyksma and Gallert 2019	Cloacimonetes bacterium	*Candidatus* Syntrophosphaera thermopropionivorans	1749786	36.6	1372		PRJNA525756	SAMN11044455	GCA_004353895	
Selenobaculum gibii ICN-92133	Yeo et al. 2024	KCTC 25622; JCM 36070	*Selenobaculum gibii*	2679003	35.5	2444		PRJNA944274	SAMN33746654	GCA_030273445	

Strain	Authority	Other deposits	Synonyms	Base pairs	Percent G+C	No. proteins	Goldstamp	Bioproject accession	Biosample accession	Assembly accession	IMG OID
bin.42.fa				1709 333	36.5	1470					

Methods, Results and References

The genome sequence data were uploaded to the Type (Strain) Genome Server (TYGS), a free bioinformatics platform available under https://tygs.dsmz.de, for a whole genome-based taxonomic analysis [1]. The analysis also made use of recently introduced methodological updates and features [2]. Information on nomenclature, synonymy and associated taxonomic literature was provided by TYGS's sister database, the List of Prokaryotic names with Standing in Nomenclature (LPSN, available at https://lpsn.dsmz.de) [2]. The results were provided by the TYGS on 2024-06-16. The TYGS analysis was subdivided into the following steps:

Determination of closely related type strains

The determination of closely related type strains did not succeed because not a single 16S rDNA gene sequence was detected in the provided user genomes. The subsequent analyses are thus only based on the provided genome data and the manually selected type strains, if any.

Pairwise comparison of genome sequences

For the phylogenomic inference, all pairwise comparisons among the set of genomes were conducted using GBDP and accurate intergenomic distances inferred under the algorithm 'trimming' and distance formula d_5 [3]. 100 distance replicates were calculated each. Digital DDH values and confidence intervals were calculated using the recommended settings of the GGDC 4.0 [2,3].

Phylogenetic inference

The resulting intergenomic distances were used to infer a balanced minimum evolution tree with branch support via FASTME 2.1.6.1 including SPR postprocessing [4]. Branch support was inferred from 100 pseudo-bootstrap replicates each. The trees were rooted at the midpoint [5] and visualized with PhyD3 [6].

Type-based species and subspecies clustering

The type-based species clustering using a 70% dDDH radius around each of the 10 type strains was done as previously described [1]. The resulting groups are shown in Table 1 and 4. Subspecies clustering was done using a 79% dDDH threshold as previously introduced [7].

Results

Type-based species and subspecies clustering

The resulting species and subspecies clusters are listed in Table 4, whereas the taxonomic identification of the query strains is found in Table 1. Briefly, the clustering yielded 9 species clusters and the provided query strains were assigned to 1 of these. Moreover, user strains were located in 1 of 10 subspecies clusters.

Figure caption genome tree

Figure 2. Tree inferred with FastME 2.1.6.1 [4] from GBDP distances calculated from genome sequences. The branch lengths are scaled in terms of GBDP distance formula d_5. The numbers above branches are GBDP pseudo-bootstrap support values > 60 % from 100 replications, with an average branch support of 85.5 %. The tree was rooted at the midpoint [5].

References

[1] Meier-Kolthoff JP, Göker M. TYGS is an automated high-throughput platform for state-of-the-art genome-based taxonomy. Nat. Commun. 2019;10: 2182. DOI: 10.1038/s41467-019-10210-3

[2] Meier-Kolthoff JP, Sardà Carbasse J, Peinado-Olarte RL, Göker M. TYGS and LPSN: a database tandem for fast and reliable genome-based classification and nomenclature of prokaryotes. Nucleic Acid Res. 2022;50: D801–D807. DOI: 10.1093/nar/gkab902

[3] Meier-Kolthoff JP, Auch AF, Klenk H-P, Göker M. Genome sequence-based species delimitation with confidence intervals and improved distance functions. BMC Bioinformatics. 2013;14: 60. DOI: 10.1186/1471-2105-14-60

[4] Lefort V, Desper R, Gascuel O. FastME 2.0: A comprehensive, accurate, and fast distance-based phylogeny inference program. Mol Biol Evol. 2015;32: 2798–2800. DOI: 10.1093/molbev/msv150

[5] Farris JS. Estimating phylogenetic trees from distance matrices. Am Nat. 1972;106: 645–667.

[6] Kreft L, Botzki A, Coppens F, Vandepoele K, Van Bel M. PhyD3: A phylogenetic tree viewer with extended phyloXML support for functional genomics data visualization. Bioinformatics. 2017;33: 2946–2947. DOI: 10.1093/bioinformatics/btx324

[7] Meier-Kolthoff JP, Hahnke RL, Petersen J, Scheuner C, Michael V, Fiebig A, et al. Complete genome sequence of DSM 30083^T, the type strain (U5/41^T) of *Escherichia coli*, and a proposal for delineating subspecies in microbial taxonomy. Stand Genomic Sci. 2014;9: 2. DOI: 10.1186/1944-3277-9-2
