# Supplementary material for: Tracing active members in microbial communities by BONCAT and click chemistry-based enrichment of newly synthesized proteins
Source: ISME Commun. 2024 Dec 4;4(1):ycae153. doi: 10.1093/ismeco/ycae153 (PMC11683836; doi:10.1093/ismeco/ycae153)
Supplement: Genome_Server_ycae153 [file genome_server_ycae153.zip › Genome Server/Bin_43_Bin_53_TYGS_job_results.pdf]

# TYPE (STRAIN) GENOME SERVER

# JOB SUMMARY

| | |
|---|---|
| PRINT DATE: | 2024-06-17 09:14:42 +0200 |
| JOB ID: | 8ce6b427-b617-4191-b16f-6815bae704ea--16 |
| RESULT PAGE: | https://tygs.dsmz.de/user_results/show?guid=8ce6b427-b617-4191-b16f-6815bae704ea--16 |

## Table 1: Phylogenies

**Publication-ready versions** of both the genome-scale GBDP tree and the 16S rRNA gene sequence tree can be customized and exported either in SVG (vector graphic) or PNG format from within the phylogeny viewers in your TYGS result page. For publications the **SVG format is recommended** because it is lossless, always keeps its high resolution and can also be easily converted to other popular formats such as PDF or EPS. Please follow the link provided above!

## Table 2: Identification

The below list contains the result of the TYGS species identification routine.

Explanation of remarks that might occur in the below table:

**remark [R1]:** The TYGS type strain database is automatically updated on an almost daily basis. However, if a particular type strain genome is not available in the TYGS database, this can have several reasons which are detailed in the FAQ. You can request an extended 16S rRNA gene analysis via the 16S tree viewer found in your result page to detect **not yet genome-sequenced** type strains relevant for your study.

**remark [R2]:** > 70% dDDH value (formula $d_4$) and (almost) minimal dDDH values for gene-content formulae $d_0$ and $d_6$ indicate a potentially unreliable identification result and should thus be checked via the 16S rRNA gene sequence similarity. Such strong deviations can, in principle, be caused by sequence contamination.

**remark [R3]:** G+C content difference of > 1 % indicates a potentially unreliable identification result because within species G+C content varies no more than 1 %, if computed from genome sequences (PMID: 24505073).

| Strain | Conclusion | Identification result | Remark |
|---|---|---|---|
| **'bin.53'** | belongs to known species | *Candidatus* Methanoculleus thermohydrogenitrophicus | |
| **'bin.43'** | potential new species | | see [R1] |

## Table 3: Pairwise comparisons of user genomes vs. type-strain genomes

The following table contains the pairwise dDDH values between your user genomes and the selected type-strain genomes. The dDDH values are provided along with their confidence intervals (C.I.) for the three different GBDP formulas:

• formula $d_0$ (a.k.a. GGDC formula 1): length of all HSPs divided by total genome length
• formula $d_4$ (a.k.a. GGDC formula 2): sum of all identities found in HSPs divided by overall HSP length
• formula $d_6$ (a.k.a. GGDC formula 3): sum of all identities found in HSPs divided by total genome length

**Note:** Formula $d_4$ is independent of genome length and is thus robust against the use of incomplete draft genomes. For other reasons for preferring formula $d_4$, see the FAQ.

| Query | Subject | $d_0$ | C.I. $d_0$ | $d_4$ | C.I. $d_4$ | $d_6$ | C.I. $d_6$ | Diff. G+C Percent |
|---|---|---|---|---|---|---|---|---|
| **'bin.53.fa'** | *Candidatus* Methanoculleus thermohydrogenitrophicus DTU006 | 83.6 | [79.8 - 86.8] | 95.1 | [93.6 - 96.3] | 88.4 | [85.5 - 90.8] | 0.03 |
| **'bin.43.fa'** | *Methanoculleus bourgensis* MS2 | 42.2 | [38.8 - 45.6] | 65.4 | [62.5 - 68.2] | 45.0 | [42.0 - 48.0] | 1.19 |
| **'bin.43.fa'** | *Candidatus* Methanoculleus thermohydrogenitrophicus DTU006 | 30.0 | [26.7 - 33.6] | 36.5 | [34.0 - 39.0] | 29.8 | [26.9 - 32.9] | 2.38 |
| **'bin.53.fa'** | *Methanoculleus bourgensis* MS2 | 40.1 | [36.8 - 43.6] | 35.2 | [32.8 - 37.7] | 38.2 | [35.3 - 41.3] | 1.16 |
| **'bin.43.fa'** | **'bin.53.fa'** | 27.6 | [24.2 - 31.2] | 33.0 | [30.6 - 35.5] | 27.1 | [24.2 - 30.3] | 2.35 |
| **'bin.43.fa'** | *Methanoculleus sediminis* DSM 29354 | 24.0 | [20.7 - 27.6] | 26.6 | [24.2 - 29.1] | 23.2 | [20.4 - 26.3] | 0.5 |
| **'bin.43.fa'** | *Methanoculleus marisnigri* JR1 | 24.8 | [21.5 - 28.5] | 26.4 | [24.1 - 28.9] | 23.9 | [21.1 - 27.0] | 0.23 |
| **'bin.43.fa'** | *Methanoculleus submarinus* DSM 15122 | 24.8 | [21.5 - 28.4] | 26.4 | [24.0 - 28.9] | 23.9 | [21.0 - 27.0] | 0.24 |
| **'bin.43.fa'** | *Methanoculleus hydrogenitrophicus* DSM 25996 | 22.5 | [19.3 - 26.2] | 26.3 | [24.0 - 28.8] | 22.0 | [19.2 - 25.1] | 0.42 |
| **'bin.43.fa'** | *Methanoculleus horonobensis* T10 | 23.6 | [20.3 - 27.2] | 26.1 | [23.8 - 28.6] | 22.8 | [20.0 - 25.9] | 0.21 |
| **'bin.53.fa'** | *Methanoculleus sediminis* DSM 29354 | 24.4 | [21.1 - 28.0] | 25.0 | [22.7 - 27.5] | 23.4 | [20.5 - 26.5] | 2.85 |
| **'bin.43.fa'** | *Methanoculleus chikugoensis* JCM 10825 | 22.1 | [18.9 - 25.8] | 25.0 | [22.7 - 27.5] | 21.5 | [18.7 - 24.6] | 0.53 |
| **'bin.53.fa'** | *Methanoculleus submarinus* DSM 15122 | 24.6 | [21.3 - 28.3] | 24.9 | [22.6 - 27.4] | 23.5 | [20.7 - 26.6] | 2.59 |
| **'bin.53.fa'** | *Methanoculleus marisnigri* JR1 | 24.7 | [21.4 - 28.3] | 24.9 | [22.6 - 27.4] | 23.6 | [20.7 - 26.7] | 2.58 |
| **'bin.53.fa'** | *Methanoculleus hydrogenitrophicus* DSM 25996 | 23.5 | [20.2 - 27.1] | 24.7 | [22.4 - 27.2] | 22.6 | [19.8 - 25.7] | 2.77 |
| **'bin.43.fa'** | *Methanoculleus thermophilus* DSM 2373 | 21.1 | [17.9 - 24.7] | 24.4 | [22.1 - 26.9] | 20.6 | [17.8 - 23.6] | 2.5 |
| **'bin.53.fa'** | *Methanoculleus horonobensis* T10 | 24.5 | [21.2 - 28.1] | 24.4 | [22.1 - 26.9] | 23.4 | [20.5 - 26.5] | 2.56 |
| **'bin.53.fa'** | *Methanoculleus chikugoensis* JCM 10825 | 21.3 | [18.1 - 25.0] | 24.0 | [21.7 - 26.5] | 20.7 | [18.0 - 23.8] | 2.88 |
| **'bin.53.fa'** | *Methanoculleus thermophilus* DSM 2373 | 22.5 | [19.3 - 26.2] | 23.4 | [21.2 - 25.9] | 21.6 | [18.9 - 24.7] | 0.15 |
| **'bin.43.fa'** | *Methanoculleus receptaculi* DSM 18860 | 16.9 | [13.9 - 20.4] | 22.5 | [20.2 - 24.9] | 16.9 | [14.3 - 19.8] | 2.74 |

| Query | Subject | $d_0$ | C.I. $d_0$ | $d_4$ | C.I. $d_4$ | $d_6$ | C.I. $d_6$ | Diff. G+C Percent |
|---|---|---|---|---|---|---|---|---|
| **'bin.53.fa'** | *Methanoculleus receptaculi* DSM 18860 | 19.3 | [16.2 - 22.9] | 22.1 | [19.8 - 24.5] | 18.9 | [16.2 - 21.9] | 0.39 |

## Table 4: Strains in your dataset

Joint dataset of automatically determined closest type strains (if this mode was chosen), manually selected type strains (if selected accordingly) and the provided user strains, if provided (marked in yellow).

| Strain | Authority | Other deposits | Synonyms | Base pairs | Percent G+C | No. proteins | Goldstamp | Bioproject accession | Biosample accession | Assembly accession | IMG OID |
|---|---|---|---|---|---|---|---|---|---|---|---|
| *Methanoculleus submarinus* DSM 15122 | Mikucki et al. 2003 | Nankai-1; OCM 780; SMCC 780W | *Methanoculleus submarinus* | 2462 514 | 62.1 | 2446 | | PRJNA891094 | SAMN31309218 | GCA_025914035 | |
| *Methanoculleus receptaculi* DSM 18860 | Cheng et al. 2008 | CGMCC 1.5087; ZC-2 | *Methanoculleus receptaculi* | 1980 381 | 59.1 | 1992 | | PRJNA1033202 | SAMN38028080 | GCA_033472595 | |
| *Methanoculleus hydrogenitrophicus* DSM 25996 | Tian et al. 2010 | CGMCC 1.5146; JCM 16311; HC | *Methanoculleus hydrogenitrophicus* | 2434 343 | 62.3 | 2494 | Gp0502680 | | | | 2913463320 |
| *Candidatus* Methanoculleus thermohydrogenitrophicus DTU006 | Kougias et al. 2017 | | *Candidatus* Methanoculleus thermohydrogenitrophicus | 2112 513 | 59.5 | 2233 | | PRJNA283298 | SAMN03776724 | GCA_001512375 | |
| *Methanoculleus thermophilus* DSM 2373 | (Rivard and Smith 1982) Maestrojuán et al. 1990 emend. Spring et al. 2005 | ATCC 33837; CR-1; OCM 174 | *Methanoculleus thermophilus*; *Methanogenium thermophilicum* | 2217 720 | 59.3 | 2249 | Gp0116609 | PRJNA303693 | SAMN04488571 | GCA_900101055 | 2634166298 |
| *Methanoculleus sediminis* DSM 29354 | Chen et al. 2015 | BCRC AR10044; S3Fa | *Methanoculleus sediminis* | 2488 374 | 62.3 | 2268 | Gp0111444 | PRJNA273166 | SAMN03288802 | GCA_001017125 | |
| *Methanoculleus horonobensis* T10 | Shimizu et al. 2013 | DSM 21626; JCM 15517 | *Methanoculleus horonobensis* | 2446 106 | 62.0 | 2435 | Gp0139332 | PRJDB4449 | SAMD00044723 | GCA_001602375 | |
| *Methanoculleus chikugoensis* JCM 10825 | Dianou et al. 2001 | DSM 13459; NBRC 101202; MG62 | *Methanoculleus chikugoensis* | 2627 540 | 62.4 | 4149 | Gp0139344 | PRJDB789 | SAMD00000410 | GCA_001315965 | |
| *Methanoculleus bourgensis* MS2 | (Ollivier et al. 1986) Maestrojuán et al. 1990 emend. Asakawa and Nagaoka 2003 | ATCC 43281; DSM 3045; OGC 15 | *Methanoculleus bourgensis*; *Methanogenium bourgense* | 2789 773 | 60.6 | 2619 | Gp0035868 | PRJEB167 | SAMEA2272323 | GCA_000304355 | 2540341105 |

| Strain | Authority | Other deposits | Synonyms | Base pairs | Percent G+C | No. proteins | Goldstamp | Bioproject accession | Biosample accession | Assembly accession | IMG OID |
|---|---|---|---|---|---|---|---|---|---|---|---|
| *Methanoculleus marisnigri* JR1 | (Romesser et al. 1981) Maestrojuán et al. 1990 | ATCC 35101; DSM 1498; OCM 56 | *Methanoculleus marisnigri*; *Methanogenium marisnigri* | 2478 095 | 62.1 | 2489 | Gp0000201 | PRJNA16330 | SAMN02598365 | GCA_000015825 | 640069318 |
| bin.43.fa | | | | 1522 531 | 61.8 | 1708 | | | | | |
| bin.53.fa | | | | 1990 662 | 59.5 | 2119 | | | | | |

## Methods, Results and References

The genome sequence data were uploaded to the Type (Strain) Genome Server (TYGS), a free bioinformatics platform available under https://tygs.dsmz.de, for a whole genome-based taxonomic analysis [1]. The analysis also made use of recently introduced methodological updates and features [2]. Information on nomenclature, synonymy and associated taxonomic literature was provided by TYGS's sister database, the List of Prokaryotic names with Standing in Nomenclature (LPSN, available at https://lpsn.dsmz.de) [2]. The results were provided by the TYGS on 2024-06-16. The TYGS analysis was subdivided into the following steps:

### Determination of closely related type strains

The determination of closely related type strains did not succeed because not a single 16S rDNA gene sequence was detected in the provided user genomes. The subsequent analyses are thus only based on the provided genome data and the manually selected type strains, if any.

### Pairwise comparison of genome sequences

For the phylogenomic inference, all pairwise comparisons among the set of genomes were conducted using GBDP and accurate intergenomic distances inferred under the algorithm 'trimming' and distance formula $d_5$ [3]. 100 distance replicates were calculated each. Digital DDH values and confidence intervals were calculated using the recommended settings of the GGDC 4.0 [2,3].

### Phylogenetic inference

The resulting intergenomic distances were used to infer a balanced minimum evolution tree with branch support via FASTME 2.1.6.1 including SPR postprocessing [4]. Branch support was inferred from 100 pseudo-bootstrap replicates each. The trees were rooted at the midpoint [5] and visualized with PhyD3 [6].

### Type-based species and subspecies clustering

The type-based species clustering using a 70% dDDH radius around each of the 10 type strains was done as previously described [1]. The resulting groups are shown in Table 1 and 4. Subspecies clustering was done using a 79% dDDH threshold as previously introduced [7].

## Results

### Type-based species and subspecies clustering

The resulting species and subspecies clusters are listed in Table 4, whereas the taxonomic identification of the query strains is found in Table 1. Briefly, the clustering yielded 10 species clusters and the provided query strains were assigned to 2 of these. Moreover, user strains were located in 2 of 10 subspecies clusters.

### Figure caption genome tree

**Figure 2.** Tree inferred with FastME 2.1.6.1 [4] from GBDP distances calculated from genome sequences. The branch lengths are scaled in terms of GBDP distance formula $d_5$. The numbers above branches are GBDP pseudo-bootstrap support values > 60 % from 100 replications, with an average branch support of 78.0 %. The tree was rooted at the midpoint [5].

## References

[1] Meier-Kolthoff JP, Göker M. TYGS is an automated high-throughput platform for state-of-the-art genome-based taxonomy. Nat. Commun. 2019;10: 2182. DOI: 10.1038/s41467-019-10210-3

[2] Meier-Kolthoff JP, Sardà Carbasse J, Peinado-Olarte RL, Göker M. TYGS and LPSN: a database tandem for fast and reliable genome-based classification and nomenclature of prokaryotes. Nucleic Acid Res. 2022;50: D801–D807. DOI: 10.1093/nar/gkab902

[3] Meier-Kolthoff JP, Auch AF, Klenk H-P, Göker M. Genome sequence-based species delimitation with confidence intervals and improved distance functions. BMC Bioinformatics. 2013;14: 60. DOI: 10.1186/1471-2105-14-60

[4] Lefort V, Desper R, Gascuel O. FastME 2.0: A comprehensive, accurate, and fast distance-based phylogeny inference program. Mol Biol Evol. 2015;32: 2798–2800. DOI: 10.1093/molbev/msv150

[5] Farris JS. Estimating phylogenetic trees from distance matrices. Am Nat. 1972;106: 645–667.

[6] Kreft L, Botzki A, Coppens F, Vandepoele K, Van Bel M. PhyD3: A phylogenetic tree viewer with extended phyloXML support for functional genomics data visualization. Bioinformatics. 2017;33: 2946–2947. DOI: 10.1093/bioinformatics/btx324

[7] Meier-Kolthoff JP, Hahnke RL, Petersen J, Scheuner C, Michael V, Fiebig A, et al. Complete genome sequence of DSM 30083$^{T}$, the type strain (U5/41$^{T}$) of *Escherichia coli*, and a proposal for delineating subspecies in microbial taxonomy. Stand Genomic Sci. 2014;9: 2. DOI: 10.1186/1944-3277-9-2
