# Supplementary material for: Tracing active members in microbial communities by BONCAT and click chemistry-based enrichment of newly synthesized proteins
Source: ISME Commun. 2024 Dec 4;4(1):ycae153. doi: 10.1093/ismeco/ycae153 (PMC11683836; doi:10.1093/ismeco/ycae153)
Supplement: Genome_Server_ycae153 [file genome_server_ycae153.zip › Genome Server/Bin_46_TYGS_job_results.pdf]

TYPE (STRAIN) GENOME SERVER

JOB SUMMARY

PRINT DATE:	2024-06-17 09:23:54 +0200
JOB ID:	8ce6b427-b617-4191-b16f-6815bae704ea--17
RESULT PAGE:	https://tygs.dsmz.de/user_results/show?guid=8ce6b427-b617-4191-b16f-6815bae704ea--17

Table 1: Phylogenies

Publication-ready versions of both the genome-scale GBDP tree and the 16S rRNA gene sequence tree can be customized and exported either in SVG (vector graphic) or PNG format from within the phylogeny viewers in your TYGS result page. For publications the **SVG format is recommended** because it is lossless, always keeps its high resolution and can also be easily converted to other popular formats such as PDF or EPS. Please follow the link provided above!

Table 2: Identification

The below list contains the result of the TYGS species identification routine.

Explanation of remarks that might occur in the below table:

remark [R1]: The TYGS type strain database is automatically updated on an almost daily basis. However, if a particular type strain genome is not available in the TYGS database, this can have several reasons which are detailed in the FAQ. You can request an extended 16S rRNA gene analysis via the 16S tree viewer found in your result page to detect **not yet genome-sequenced** type strains relevant for your study.

remark [R2]: > 70% dDDH value (formula d_4) and (almost) minimal dDDH values for gene-content formulae d_0 and d_6 indicate a potentially unreliable identification result and should thus be checked via the 16S rRNA gene sequence similarity. Such strong deviations can, in principle, be caused by sequence contamination.

remark [R3]: G+C content difference of > 1 % indicates a potentially unreliable identification result because within species G+C content varies no more than 1 %, if computed from genome sequences (PMID: 24505073).

Strain	Conclusion	Identification result	Remark
**'bin.46'**	potential new species		see [R1]

Table 3: Pairwise comparisons of user genomes vs. type-strain genomes

The following table contains the pairwise dDDH values between your user genomes and the selected type-strain genomes. The dDDH values are provided along with their confidence intervals (C.I.) for the three different GBDP formulas:

• formula d_0 (a.k.a. GGDC formula 1): length of all HSPs divided by total genome length
• formula d_4 (a.k.a. GGDC formula 2): sum of all identities found in HSPs divided by overall HSP length
• formula d_6 (a.k.a. GGDC formula 3): sum of all identities found in HSPs divided by total genome length

Note: Formula d_4 is independent of genome length and is thus robust against the use of incomplete draft genomes. For other reasons for preferring formula d_4, see the FAQ.

Query	Subject	d_0	C.I. d_0	d_4	C.I. d_4	d_6	C.I. d_6	Diff. G+C Percent
**'bin.46.fa'**	*Yanghanlia caeni* LG-2	12.5	[9.8 - 15.8]	34.1	[31.7 - 36.6]	12.9	[10.6 - 15.7]	2.33
**'bin.46.fa'**	*Limisphaera ngatamarikiensis* NGM72.4	12.5	[9.8 - 15.7]	21.7	[19.5 - 24.2]	12.9	[10.6 - 15.6]	4.42
**'bin.46.fa'**	*Frankia gtarii* Agncl-4T	12.5	[9.8 - 15.7]	21.5	[19.3 - 24.0]	12.9	[10.6 - 15.6]	10.95
**'bin.46.fa'**	*Streptomyces phaeofaciens* JCM 4125	12.5	[9.8 - 15.7]	20.7	[18.5 - 23.1]	12.9	[10.6 - 15.6]	11.23
**'bin.46.fa'**	*Kiritimatiella glycovorans* DSM 26986	12.5	[9.8 - 15.8]	18.4	[16.2 - 20.7]	12.9	[10.6 - 15.7]	2.77
**'bin.46.fa'**	*Victivallis lenta* DSM 107290	12.5	[9.9 - 15.8]	17.8	[15.7 - 20.2]	12.9	[10.6 - 15.7]	1.2
**'bin.46.fa'**	*Luteolibacter marinus* NBU1238	12.5	[9.8 - 15.8]	17.5	[15.3 - 19.8]	12.9	[10.6 - 15.6]	4.46
**'bin.46.fa'**	*Leucobacter rhizosphaerae* KACC 21837	12.5	[9.8 - 15.8]	16.3	[14.2 - 18.6]	12.9	[10.6 - 15.6]	8.76
**'bin.46.fa'**	*Pontiella sulfatireligans* F21 T	12.5	[9.8 - 15.8]	16.2	[14.2 - 18.5]	12.9	[10.6 - 15.6]	6.1
**'bin.46.fa'**	*Pseudonocardia humida* S2-4	12.5	[9.8 - 15.7]	15.4	[13.4 - 17.7]	12.9	[10.6 - 15.6]	14.02
**'bin.46.fa'**	*Streptomyces echinoruber* JCM 5016	12.5	[9.8 - 15.8]	15.4	[13.4 - 17.7]	12.9	[10.6 - 15.6]	12.66
**'bin.46.fa'**	*Variovorax beijingensis* 502T	12.5	[9.8 - 15.7]	13.9	[12.0 - 16.1]	12.9	[10.6 - 15.6]	6.86
**'bin.46.fa'**	*Aureimonas leprariae* YIM 132180T	12.5	[9.8 - 15.7]	3.7	[2.8 - 4.8]	12.9	[10.6 - 15.6]	7.88
**'bin.46.fa'**	*Candidatus* Solincola tengchongensis DRTY-6.bin.58	12.5	[9.8 - 15.7]	3.7	[2.8 - 4.8]	12.9	[10.6 - 15.6]	2.98
**'bin.46.fa'**	*Defluviimonas sediminis* FT324T	12.5	[9.8 - 15.7]	3.7	[2.8 - 4.8]	12.9	[10.6 - 15.6]	6.46
**'bin.46.fa'**	*Oleidesulfovibrio alaskensis* DSM 16109	12.5	[9.8 - 15.7]	3.7	[2.8 - 4.8]	12.9	[10.6 - 15.6]	2.53
**'bin.46.fa'**	*Halohasta litchfieldiae* DSM 22187	12.5	[9.8 - 15.7]	3.7	[2.8 - 4.8]	12.9	[10.6 - 15.6]	1.74
**'bin.46.fa'**	*Roseibium denhamense* DSM 15949	12.5	[9.8 - 15.7]	3.7	[2.8 - 4.8]	12.9	[10.6 - 15.6]	3.25
**'bin.46.fa'**	*Variovorax paradoxus* NBRC 15149	12.5	[9.8 - 15.7]	3.7	[2.8 - 4.8]	12.9	[10.6 - 15.6]	7.2
**'bin.46.fa'**	*Methylomarinum vadi* IT-4	12.5	[9.8 - 15.7]	3.7	[2.8 - 4.8]	12.9	[10.6 - 15.6]	9.26

Table 4: Strains in your dataset

Joint dataset of automatically determined closest type strains (if this mode was chosen), manually selected type strains (if selected accordingly) and the provided user strains, if provided (marked in yellow).

Strain	Authority	Other deposits	Synonyms	Base pairs	Percent G+C	No. proteins	Goldstamp	Bioproject accession	Biosample accession	Assembly accession	IMG OID
Victivallis lenta DSM 107290	Wylensek et al. 2021	JCM 34375; BBE-744-WT-12	*Victivallis lenta*	5543 238	59.3	4456		PRJNA224116	SAMN12619173	GCF_009695545	
Aureimonas leprariae YIM 132180T	Zhang et al. 2021	CGMCC 1.17389; KCTC 72462	*Aureimonas leprariae*	4779 519	68.4	4511		PRJNA573581	SAMN12817691	GCA_008802405	
Limisphaera ngatamarikiensis NGM72.4	Anders et al. 2015	ICMP 20182; DSM 27329	*Limisphaera ngatamarikiensis*	3891 337	65.0	2810		PRJNA606647	SAMN14102279	GCA_011044775	
Oleidesulfovibrio alaskensis DSM 16109	(Feio et al. 2004) Waite et al. 2020	Al; Al1; NCIMB 13491	*Desulfovibrio alaskensis*; *Oleidesulfovibrio alaskensis*	3556 414	58.0	3202	Gp0013958	PRJNA188879	SAMN02441442	GCA_000482745	2528311093
Roseibium denhamense DSM 15949	Suzuki et al. 2000 emend. Hördt et al. 2020	CIP 107047; ATCC BAA-251; JCM 10543; IFO 16782; NBRC 16782; OCh 254	*Roseibium denhamense*	4848 572	57.3	4447	Gp0194237	PRJNA363564	SAMN06265374	GCA_900182805	2724679813
Kiritimatiella glycovorans DSM 26986	Spring et al. 2017	JCM 19195; L21-Fru-AB	*Kiritimatiella glycovorans*	2949 723	63.3	2420	Gp0110288	PRJNA274602	SAMN03329217	GCA_001017655	
Yanghanlia caeni LG-2	Ruan et al. 2024	KCTC 8084; CCTCC AB 2023123	*Yanghanlia caeni*	3202 748	62.9	2963		PRJNA1002479	SAMN36845266	GCA_030733815	
Pontiella sulfatireligans F21 T	van Vliet et al. 2020	KCTC 15642; DSM 106829	*Pontiella sulfatireligans*	7395 795	54.4	5640		PRJEB24761	SAMEA5207385	GCA_900890705	

Strain	Authority	Other deposits	Synonyms	Base pairs	Percent G+C	No. proteins	Goldstamp	Bioproject accession	Biosample accession	Assembly accession	IMG OID
Variovorax paradoxus NBRC 15149	(Davis 1969) Willems et al. 1991	LMG 1797; CIP 103459; ATCC 17713; CCUG 1777; DSM 30034; DSM 66; JCM 20526; JCM 20895; IFO 15149; VKM B-1329	*Alcaligenes paradoxus*; *Variovorax paradoxus*	6664 268	67.7	6213	Gp0042371	PRJDB1090	SAMD00046749	GCA_001591365	
Streptomyces phaeofaciens JCM 4125	Maeda et al. 1952	IFM 1177; NRRL B-1516; NRRL ISP-5367; CBS 426.64; CBS 673.72; ATCC 15034; DSM 40367; JCM 4814; IFO 13372; NBRC 13372; VKM Ac-1865; RIA 1333	*Streptomyces phaeofaciens*	1040 6418	71.8	9270		PRJDB10510	SAMD00245390	GCA_014648915	
Streptomyces echinoruber JCM 5016	Palleroni et al. 1981	AS 4.1707; CGMCC 4.1707; KCTC 9725; NRRL 8144; NCIMB 12831; DSM 41696; IFO 14238; NBRC 14238; X-14077	*Streptomyces echinoruber*	7326 606	73.2	6669		PRJDB10510	SAMD00245545	GCA_014651135	
Methylomarinum vadi IT-4	Hirayama et al. 2013	DSM 18976; JCM 13665	*Methylomarinum vadi*	4335 162	51.3	4038	Gp0035621	PRJNA246204	SAMN02745036	GCA_000733935	2574179779

Strain	Authority	Other deposits	Synonyms	Base pairs	Percent G+C	No. proteins	Goldstamp	Bioproject accession	Biosample accession	Assembly accession	IMG OID
Halohasta litchfieldiae DSM 22187	Mou et al. 2013	JCM 15066; tADL	*Halohasta litchfieldiae*	3284 590	58.8	3434	Gp0148826	PRJNA335225	SAMN05444271	GCA_900109065	2693429898
Variovorax beijingensis 502T	Gao et al. 2020	CGMCC 1.16560; DSM 106862	*Variovorax beijingensis*	6762 924	67.4	6310		PRJNA224116	SAMN10578850	GCF_003951285	
Pseudonocardia humida S2-4	Zan et al. 2022	CGMCC 4.7706; JCM 34291	*Pseudonocardia humida*	8281 783	74.6	7781		PRJNA722583	SAMN18780209	GCA_023898865	
Luteolibacter marinus NBU1238	Xie et al. 2022	MCCC 1K04772; KCTC 82227	*Luteolibacter marinus*	6103 989	65.0	4871		PRJNA666138	SAMN16280491	GCA_014904755	
Defluviimonas sediminis FT324T	Liu et al. 2023	MCCC 1K07685; KCTC 92477	*Defluviimonas sediminis*	4287 551	67.0	4159		PRJNA224116	SAMN30734388	GCF_025380365	
Candidatus Solincola tengchongensis DRTY-6.bin.58	Jiao et al. 2021		*Candidatus* Solincola tengchongensis	1355 422	63.5	1209		PRJNA649850	SAMN15683674	GCA_015711895	
Leucobacter rhizosphaerae KACC 21837	Kim et al. 2023	DSM 114346; JCM 35239; NBRC 115479; H25R-14	*Leucobacter rhizosphaerae*	3337 834	69.3	2996		PRJNA822884	SAMN27281701	GCA_022919175	
Frankia gtarii Agncl-4T	Nouioui et al. 2023	CECT 9711; DSM 107976	*Frankia gtarii*	7354 442	71.5	5861		PRJNA224116	SAMN28795899	GCF_025403375	
bin.46.fa				3007 150	60.5	2596					

Methods, Results and References

The genome sequence data were uploaded to the Type (Strain) Genome Server (TYGS), a free bioinformatics platform available under https://tygs.dsmz.de, for a whole genome-based taxonomic analysis [1]. The analysis also made use of recently introduced methodological updates and features [2]. Information on nomenclature, synonymy and associated taxonomic literature was provided by TYGS's sister database, the List of Prokaryotic names with Standing in Nomenclature (LPSN, available at https://lpsn.dsmz.de) [2]. The results were provided by the TYGS on 2024-06-16. The TYGS analysis was subdivided into the following steps:

Determination of closely related type strains

Determination of closest type strain genomes was done in two complementary ways: First, all user genomes were compared against all type strain genomes available in the TYGS database via the MASH algorithm, a fast approximation of intergenomic relatedness [3], and, the ten type strains with the smallest MASH distances chosen per user genome. Second, an additional set of ten closely related type strains was determined via the 16S rDNA gene sequences. These were extracted from the user genomes using RNAmmer [4] and each sequence was subsequently BLASTed [5] against the 16S rDNA gene sequence of each of the currently 21253 type strains available in the TYGS database. This was used as a proxy to find the best 50 matching type strains (according to the bitscore) for each user genome and to subsequently calculate precise distances using the Genome BLAST Distance Phylogeny approach (GBDP) under the algorithm 'coverage' and distance formula d_5 [6]. These distances were finally used to determine the 10 closest type strain genomes for each of the user genomes.

Pairwise comparison of genome sequences

For the phylogenomic inference, all pairwise comparisons among the set of genomes were conducted using GBDP and accurate intergenomic distances inferred under the algorithm 'trimming' and distance formula d_5 [6]. 100 distance replicates were calculated each. Digital DDH values and confidence intervals were calculated using the recommended settings of the GGDC 4.0 [2,6].

Phylogenetic inference

The resulting intergenomic distances were used to infer a balanced minimum evolution tree with branch support via FASTME 2.1.6.1 including SPR postprocessing [7]. Branch support was inferred from 100 pseudo-bootstrap replicates each. The trees were rooted at the midpoint [8] and visualized with PhyD3 [9].

Type-based species and subspecies clustering

The type-based species clustering using a 70% dDDH radius around each of the 20 type strains was done as previously described [1]. The resulting groups are shown in Table 1 and 4. Subspecies clustering was done using a 79% dDDH threshold as previously introduced [10].

Results

Type-based species and subspecies clustering

The resulting species and subspecies clusters are listed in Table 4, whereas the taxonomic identification of the query strains is found in Table 1. Briefly, the clustering yielded 21 species clusters and the provided query strains were assigned to 1 of these. Moreover, user strains were located in 1 of 21 subspecies clusters.

Figure caption SSU tree

Figure 1. Tree inferred with FastME 2.1.6.1 [7] from GBDP distances calculated from 16S rDNA gene sequences. The branch lengths are scaled in terms of GBDP distance formula d_5. The numbers above branches are GBDP pseudo-bootstrap support values > 60 % from 100 replications, with an average branch support of 86.2 %. The tree was rooted at the midpoint [8].

Figure caption genome tree

Figure 2. Tree inferred with FastME 2.1.6.1 [7] from GBDP distances calculated from genome sequences. The branch lengths are scaled in terms of GBDP distance formula d_5. The numbers above branches are GBDP pseudo-bootstrap support values > 60 % from 100 replications, with an average branch support of 50.7 %. The tree was rooted at the midpoint [8].

References

[1] Meier-Kolthoff JP, Göker M. TYGS is an automated high-throughput platform for state-of-the-art genome-based taxonomy. Nat. Commun. 2019;10: 2182. DOI: 10.1038/s41467-019-10210-3

[2] Meier-Kolthoff JP, Sardà Carbasse J, Peinado-Olarte RL, Göker M. TYGS and LPSN: a database tandem for fast and reliable genome-based classification and nomenclature of prokaryotes. Nucleic Acid Res. 2022;50: D801–D807. DOI: 10.1093/nar/gkab902

[3] Ondov BD, Treangen TJ, Melsted P, et al. Mash: Fast genome and metagenome distance estimation using MinHash. Genome Biol 2016;17: 1–14. DOI: 10.1186/s13059-016-0997-x

[4] Lagesen K, Hallin P. RNAmmer: consistent and rapid annotation of ribosomal RNA genes. Nucleic Acids Res. Oxford Univ Press; 2007;35: 3100–3108. DOI: 10.1093/nar/gkm160

[5] Camacho C, Coulouris G, Avagyan V, Ma N, Papadopoulos J, Bealer K, et al. BLAST+: architecture and applications. BMC Bioinformatics. 2009;10: 421. DOI: 10.1186/1471-2105-10-421

[6] Meier-Kolthoff JP, Auch AF, Klenk H-P, Göker M. Genome sequence-based species delimitation with confidence intervals and improved distance functions. BMC Bioinformatics. 2013;14: 60. DOI: 10.1186/1471-2105-14-60

[7] Lefort V, Desper R, Gascuel O. FastME 2.0: A comprehensive, accurate, and fast distance-based phylogeny inference program. Mol Biol Evol. 2015;32: 2798–2800. DOI: 10.1093/molbev/msv150

[8] Farris JS. Estimating phylogenetic trees from distance matrices. Am Nat. 1972;106: 645–667.

[9] Kreft L, Botzki A, Coppens F, Vandepoele K, Van Bel M. PhyD3: A phylogenetic tree viewer with extended phyloXML support for functional genomics data visualization. Bioinformatics. 2017;33: 2946–2947. DOI: 10.1093/bioinformatics/btx324

[10] Meier-Kolthoff JP, Hahnke RL, Petersen J, Scheuner C, Michael V, Fiebig A, et al. Complete genome sequence of DSM 30083^{T}, the type strain (U5/41^{T}) of *Escherichia coli*, and a proposal for delineating subspecies in microbial taxonomy. Stand Genomic Sci. 2014;9: 2. DOI: 10.1186/1944-3277-9-2
