# Supplementary material for: Tracing active members in microbial communities by BONCAT and click chemistry-based enrichment of newly synthesized proteins
Source: ISME Commun. 2024 Dec 4;4(1):ycae153. doi: 10.1093/ismeco/ycae153 (PMC11683836; doi:10.1093/ismeco/ycae153)
Supplement: Genome_Server_ycae153 [file genome_server_ycae153.zip › Genome Server/Bin_48_TYGS_job_results.pdf]

TYPE (STRAIN) GENOME SERVER

JOB SUMMARY

PRINT DATE:	2024-06-17 09:18:53 +0200
JOB ID:	8ce6b427-b617-4191-b16f-6815bae704ea--18
RESULT PAGE:	https://tygs.dsmz.de/user_results/show?guid=8ce6b427-b617-4191-b16f-6815bae704ea--18

Table 1: Phylogenies

Publication-ready versions of both the genome-scale GBDP tree and the 16S rRNA gene sequence tree can be customized and exported either in SVG (vector graphic) or PNG format from within the phylogeny viewers in your TYGS result page. For publications the **SVG format is recommended** because it is lossless, always keeps its high resolution and can also be easily converted to other popular formats such as PDF or EPS. Please follow the link provided above!

Table 2: Identification

The below list contains the result of the TYGS species identification routine.

Explanation of remarks that might occur in the below table:

remark [R1]: The TYGS type strain database is automatically updated on an almost daily basis. However, if a particular type strain genome is not available in the TYGS database, this can have several reasons which are detailed in the FAQ. You can request an extended 16S rRNA gene analysis via the 16S tree viewer found in your result page to detect **not yet genome-sequenced** type strains relevant for your study.

remark [R2]: > 70% dDDH value (formula d_4) and (almost) minimal dDDH values for gene-content formulae d_0 and d_6 indicate a potentially unreliable identification result and should thus be checked via the 16S rRNA gene sequence similarity. Such strong deviations can, in principle, be caused by sequence contamination.

remark [R3]: G+C content difference of > 1 % indicates a potentially unreliable identification result because within species G+C content varies no more than 1 %, if computed from genome sequences (PMID: 24505073).

Strain	Conclusion	Identification result	Remark
**'bin.48'**	potential new species		see [R1]

Table 3: Pairwise comparisons of user genomes vs. type-strain genomes

The following table contains the pairwise dDDH values between your user genomes and the selected type-strain genomes. The dDDH values are provided along with their confidence intervals (C.I.) for the three different GBDP formulas:

- formula d_0 (a.k.a. GGDC formula 1): length of all HSPs divided by total genome length
- formula d_4 (a.k.a. GGDC formula 2): sum of all identities found in HSPs divided by overall HSP length
- formula d_6 (a.k.a. GGDC formula 3): sum of all identities found in HSPs divided by total genome length

Note: Formula d_4 is independent of genome length and is thus robust against the use of incomplete draft genomes. For other reasons for preferring formula d_4, see the FAQ.

Query	Subject	d_0	C.I. d_0	d_4	C.I. d_4	d_6	C.I. d_6	Diff. G+C Percent
**'bin.48.fa'**	*Agathobaculum desmolans* ATCC 43058	12.5	[9.9 - 15.8]	66.4	[63.4 - 69.2]	12.9	[10.6 - 15.7]	14.11
**'bin.48.fa'**	*Annamia dubia* NIES 4383	12.5	[9.8 - 15.7]	43.1	[40.6 - 45.6]	12.9	[10.6 - 15.6]	6.76
**'bin.48.fa'**	*Acetivibrio saccincola* DSM 101079	12.6	[9.9 - 15.8]	41.4	[38.9 - 44.0]	13.0	[10.6 - 15.7]	5.21
**'bin.48.fa'**	*Defluviitalea raffinosedens* DSM 28090	12.5	[9.9 - 15.8]	36.3	[33.8 - 38.8]	13.0	[10.6 - 15.7]	4.25
**'bin.48.fa'**	*Fonticella tunisiensis* DSM 24455	12.5	[9.8 - 15.8]	35.3	[32.9 - 37.9]	12.9	[10.6 - 15.7]	3.04
**'bin.48.fa'**	*Acetivibrio mesophilus* DSM 107956	12.6	[9.9 - 15.9]	32.8	[30.4 - 35.3]	13.0	[10.7 - 15.7]	1.99
**'bin.48.fa'**	*Ruminiclostridium josui* JCM 17888	12.6	[9.9 - 15.8]	31.7	[29.3 - 34.2]	13.0	[10.7 - 15.7]	3.94
**'bin.48.fa'**	*Clostridium thermosuccinogenes* DSM 5807	12.6	[9.9 - 15.9]	29.6	[27.2 - 32.1]	13.0	[10.7 - 15.8]	1.52
**'bin.48.fa'**	*Thermoclostridium caenicola* DSM 19027	12.7	[10.0 - 16.0]	21.6	[19.3 - 24.0]	13.1	[10.7 - 15.8]	10.92
**'bin.48.fa'**	*Maribellus sediminis* Y2-1-60	12.5	[9.8 - 15.7]	14.6	[12.7 - 16.9]	12.9	[10.6 - 15.6]	1.99

Table 4: Strains in your dataset

Joint dataset of automatically determined closest type strains (if this mode was chosen), manually selected type strains (if selected accordingly) and the provided user strains, if provided (marked in yellow).

Strain	Authority	Other deposits	Synonyms	Base pairs	Percent G+C	No. proteins	Goldstamp	Bioproject accession	Biosample accession	Assembly accession	IMG OID
Ruminiclostridium josui JCM 17888	(Sukhumavasi et al. 1988) Zhang et al. 2018	DSM 25723; FERM P-9684; III	*Clostridium josui*; *Ruminiclostridium josui*	4469680	36.1	3892	Gp0033251	PRJNA195880	SAMN02584941	GCA_000526495	2541046955
Acetivibrio saccincola DSM 101079	(Koeck et al. 2016) Tindall 2019	CECT 9155; GGR1	*Acetivibrio saccincola*; *Herbivorax saccincola*; *Hungateiclostridium saccincola*	3604547	34.8	3125	Gp0302128	PRJNA398183	SAMN07503206	GCA_002844395	
Defluviitalea raffinosedens DSM 28090	Ma et al. 2017	A6; ACCC 19951	*Defluviitalea raffinosedens*	3191925	35.8	3027	Gp0505781				2901198348
Thermoclostridium caenicola DSM 19027	(Shiratori et al. 2009) Zhang et al. 2018	NBRC 102590; EBR596	*Clostridium caenicola*; *Thermoclostridium caenicola*	2890049	50.9	2618	Gp0148792	PRJNA335191	SAMN05444373	GCA_900142095	2700988698
Annamia dubia NIES 4383	Tuji et al. 2021	TNS-AL 58964	*Annamia dubia*	4023243	33.3	3581		PRJDB10714	SAMD00282208		
Acetivibrio mesophilus DSM 107956	(Rettenmaier et al. 2019) Tindall 2019	CECT 9704; N2K1	*Acetivibrio mesophilus*; *Hungateiclostridium mesophilum*	4039456	38.0	3299		PRJNA504010	SAMN10395150	GCA_004102745	
Agathobaculum desmolans ATCC 43058	(Morris et al. 1986) Ahn et al. 2016	CCUG 27818; JCM 6566	*Agathobaculum desmolans*; *Butyricicoccus desmolans*; *Eubacterium desmolans*	2649504	54.1	2695	Gp0044078	PRJNA223055	SAMN02841159	GCA_000701665	2561511082
Clostridium thermosuccinogenes DSM 5807	Drent et al. 1995	IC	*Clostridium thermosuccinogenes*; *Pseudoclostridium thermosuccinogenes*	4731216	41.5	3940	Gp0307796	PRJNA388583	SAMN07200985	GCA_002896855	
Maribellus sediminis Y2-1-60	Huang et al. 2020	MCCC 1K04285; KCTC 72884	*Maribellus sediminis*	5589935	42.0	4343		PRJNA598407	SAMN13705161	GCA_011799805	

Strain	Authority	Other deposits	Synonyms	Base pairs	Percent G+C	No. proteins	Goldstamp	Bioproject accession	Biosample accession	Assembly accession	IMG OID
Fonticella tunisiensis DSM 24455	Fraj et al. 2013	JCM 17559; BELH25	*Fonticella tunisiensis*	3097 060	37.0	2947	Gp0290634	PRJNA500711	SAMN10362907	GCA_004364155	2784746797
bin.48.fa				5523 114	40.0	5142					

Methods, Results and References

The genome sequence data were uploaded to the Type (Strain) Genome Server (TYGS), a free bioinformatics platform available under https://tygs.dsmz.de, for a whole genome-based taxonomic analysis [1]. The analysis also made use of recently introduced methodological updates and features [2]. Information on nomenclature, synonymy and associated taxonomic literature was provided by TYGS's sister database, the List of Prokaryotic names with Standing in Nomenclature (LPSN, available at https://lpsn.dsmz.de) [2]. The results were provided by the TYGS on 2024-06-16. The TYGS analysis was subdivided into the following steps:

Determination of closely related type strains

The determination of closely related type strains did not succeed because not a single 16S rDNA gene sequence was detected in the provided user genomes. The subsequent analyses are thus only based on the provided genome data and the manually selected type strains, if any.

Pairwise comparison of genome sequences

For the phylogenomic inference, all pairwise comparisons among the set of genomes were conducted using GBDP and accurate intergenomic distances inferred under the algorithm 'trimming' and distance formula d_5 [3]. 100 distance replicates were calculated each. Digital DDH values and confidence intervals were calculated using the recommended settings of the GGDC 4.0 [2,3].

Phylogenetic inference

The resulting intergenomic distances were used to infer a balanced minimum evolution tree with branch support via FASTME 2.1.6.1 including SPR postprocessing [4]. Branch support was inferred from 100 pseudo-bootstrap replicates each. The trees were rooted at the midpoint [5] and visualized with PhyD3 [6].

Type-based species and subspecies clustering

The type-based species clustering using a 70% dDDH radius around each of the 10 type strains was done as previously described [1]. The resulting groups are shown in Table 1 and 4. Subspecies clustering was done using a 79% dDDH threshold as previously introduced [7].

Results

Type-based species and subspecies clustering

The resulting species and subspecies clusters are listed in Table 4, whereas the taxonomic identification of the query strains is found in Table 1. Briefly, the clustering yielded 11 species clusters and the provided query strains were assigned to 1 of these. Moreover, user strains were located in 1 of 11 subspecies clusters.

Figure caption genome tree

Figure 2. Tree inferred with FastME 2.1.6.1 [4] from GBDP distances calculated from genome sequences. The branch lengths are scaled in terms of GBDP distance formula d_5. The numbers above branches are GBDP pseudo-bootstrap support values > 60 % from 100 replications, with an average branch support of 35.6 %. The tree was rooted at the midpoint [5].

References

[1] Meier-Kolthoff JP, Göker M. TYGS is an automated high-throughput platform for state-of-the-art genome-based taxonomy. Nat. Commun. 2019;10: 2182. DOI: 10.1038/s41467-019-10210-3

[2] Meier-Kolthoff JP, Sardà Carbasse J, Peinado-Olarte RL, Göker M. TYGS and LPSN: a database tandem for fast and reliable genome-based classification and nomenclature of prokaryotes. Nucleic Acid Res. 2022;50: D801–D807. DOI: 10.1093/nar/gkab902

[3] Meier-Kolthoff JP, Auch AF, Klenk H-P, Göker M. Genome sequence-based species delimitation with confidence intervals and improved distance functions. BMC Bioinformatics. 2013;14: 60. DOI: 10.1186/1471-2105-14-60

[4] Lefort V, Desper R, Gascuel O. FastME 2.0: A comprehensive, accurate, and fast distance-based phylogeny inference program. Mol Biol Evol. 2015;32: 2798–2800. DOI: 10.1093/molbev/msv150

[5] Farris JS. Estimating phylogenetic trees from distance matrices. Am Nat. 1972;106: 645–667.

[6] Kreft L, Botzki A, Coppens F, Vandepoele K, Van Bel M. PhyD3: A phylogenetic tree viewer with extended phyloXML support for functional genomics data visualization. Bioinformatics. 2017;33: 2946–2947. DOI: 10.1093/bioinformatics/btx324

[7] Meier-Kolthoff JP, Hahnke RL, Petersen J, Scheuner C, Michael V, Fiebig A, et al. Complete genome sequence of DSM 30083^{T}, the type strain (U5/41^{T}) of *Escherichia coli*, and a proposal for delineating subspecies in microbial taxonomy. Stand Genomic Sci. 2014;9: 2. DOI: 10.1186/1944-3277-9-2
