# Supplementary material for: Tracing active members in microbial communities by BONCAT and click chemistry-based enrichment of newly synthesized proteins
Source: ISME Commun. 2024 Dec 4;4(1):ycae153. doi: 10.1093/ismeco/ycae153 (PMC11683836; doi:10.1093/ismeco/ycae153)
Supplement: Genome_Server_ycae153 [file genome_server_ycae153.zip › Genome Server/Bin_58_TYGS_job_results.pdf]

TYPE (STRAIN) GENOME SERVER

JOB SUMMARY

PRINT DATE:	2024-06-17 09:21:21 +0200
JOB ID:	8ce6b427-b617-4191-b16f-6815bae704ea--19
RESULT PAGE:	https://tygs.dsmz.de/user_results/show?guid=8ce6b427-b617-4191-b16f-6815bae704ea--19

Table 1: Phylogenies

Publication-ready versions of both the genome-scale GBDP tree and the 16S rRNA gene sequence tree can be customized and exported either in SVG (vector graphic) or PNG format from within the phylogeny viewers in your TYGS result page. For publications the **SVG format is recommended** because it is lossless, always keeps its high resolution and can also be easily converted to other popular formats such as PDF or EPS. Please follow the link provided above!

Table 2: Identification

The below list contains the result of the TYGS species identification routine.

Explanation of remarks that might occur in the below table:

remark [R1]: The TYGS type strain database is automatically updated on an almost daily basis. However, if a particular type strain genome is not available in the TYGS database, this can have several reasons which are detailed in the FAQ. You can request an extended 16S rRNA gene analysis via the 16S tree viewer found in your result page to detect **not yet genome-sequenced** type strains relevant for your study.

remark [R2]: > 70% dDDH value (formula d_4) and (almost) minimal dDDH values for gene-content formulae d_0 and d_6 indicate a potentially unreliable identification result and should thus be checked via the 16S rRNA gene sequence similarity. Such strong deviations can, in principle, be caused by sequence contamination.

remark [R3]: G+C content difference of > 1 % indicates a potentially unreliable identification result because within species G+C content varies no more than 1 %, if computed from genome sequences (PMID: 24505073).

Strain	Conclusion	Identification result	Remark
**'bin.58'**	potential new species		see [R1]

Table 3: Pairwise comparisons of user genomes vs. type-strain genomes

The following table contains the pairwise dDDH values between your user genomes and the selected type-strain genomes. The dDDH values are provided along with their confidence intervals (C.I.) for the three different GBDP formulas:

- formula d_0 (a.k.a. GGDC formula 1): length of all HSPs divided by total genome length
- formula d_4 (a.k.a. GGDC formula 2): sum of all identities found in HSPs divided by overall HSP length
- formula d_6 (a.k.a. GGDC formula 3): sum of all identities found in HSPs divided by total genome length

Note: Formula d_4 is independent of genome length and is thus robust against the use of incomplete draft genomes. For other reasons for preferring formula d_4, see the FAQ.

Query	Subject	d_0	C.I. d_0	d_4	C.I. d_4	d_6	C.I. d_6	Diff. G+C Percent
**'bin.58.fa'**	*Aeromonas encheleia* CECT 4342	12.5	[9.8 - 15.7]	17.9	[15.8 - 20.3]	12.9	[10.6 - 15.6]	1.09
**'bin.58.fa'**	*Aeromonas hydrophila* ATCC 7966	12.5	[9.8 - 15.7]	17.5	[15.4 - 19.9]	12.9	[10.6 - 15.6]	0.68
**'bin.58.fa'**	*Streptomyces demainii* DSM 41600	12.5	[9.8 - 15.7]	17.0	[14.9 - 19.3]	12.9	[10.6 - 15.6]	10.98
**'bin.58.fa'**	*Nocardioides flavescens* YIM 123512	12.5	[9.8 - 15.8]	16.4	[14.4 - 18.7]	12.9	[10.6 - 15.6]	11.89
**'bin.58.fa'**	*Streptomyces sporocinereus* NBRC 100766	12.5	[9.8 - 15.7]	15.9	[13.9 - 18.2]	12.9	[10.6 - 15.6]	11.15
**'bin.58.fa'**	*Streptomyces malaysiense* MUSC 136	12.5	[9.8 - 15.8]	15.5	[13.5 - 17.8]	12.9	[10.6 - 15.6]	11.36
**'bin.58.fa'**	*Tepidiforma thermophila* G233T	12.6	[9.9 - 15.8]	15.1	[13.0 - 17.3]	13.0	[10.6 - 15.7]	8.5
**'bin.58.fa'**	*Aeromonas hydrophila* subsp. *ranae* CIP 107985	12.5	[9.8 - 15.7]	14.6	[12.6 - 16.8]	12.9	[10.6 - 15.6]	0.68
**'bin.58.fa'**	*Streptomyces pluricolorescens* JCM 4602	12.5	[9.8 - 15.8]	14.2	[12.2 - 16.4]	12.9	[10.6 - 15.6]	10.83
**'bin.58.fa'**	*Nocardioides bruguierae* BSK12Z-3T	12.5	[9.8 - 15.8]	14.2	[12.3 - 16.4]	12.9	[10.6 - 15.6]	12.62

Table 4: Strains in your dataset

Joint dataset of automatically determined closest type strains (if this mode was chosen), manually selected type strains (if selected accordingly) and the provided user strains, if provided (marked in yellow).

Strain	Authority	Other deposits	Synonyms	Base pairs	Percent G+C	No. proteins	Goldstamp	Bioproject accession	Biosample accession	Assembly accession	IMG OID
Nocardioides bruguierae BSK12Z-3T	Chen et al. 2023	CGMCC 4.7709; JCM 34554	*Nocardioides bruguierae*	4381 337	73.5	3995		PRJNA224116	SAMN28546152	GCF_023554635	
Nocardioides flavescens YIM 123512	Zhang et al. 2020	CGMCC 4.7628; KCTC 49303	*Nocardioides flavescens*	4633 568	72.8	4296		PRJNA597291	SAMN13671869	GCA_009823805	
Streptomyces demainii DSM 41600	Goodfellow et al. 2008	NRRL B-1478	*Streptomyces demainii*	1054 2352	71.8	8775	Gp0502523				2923609139
Streptomyces malaysiense MUSC 136	Ser et al. 2016	MCCC 1K01246; DSM 100712	*Streptomyces malaysiense*	7929 788	72.2	7025	Gp0119032	PRJNA224116	SAMN03445833	GCF_000980885	
Streptomyces sporocinereus NBRC 100766	(ex Krassilnikov 1970) Preobrazhenskaya 1986 emend. Nouioui et al. 2018	NRRL B-16376; ATCC 43692; DSM 41460; JCM 9093; VKM Ac-312; INMI 32	*Streptomyces hygroscopicus* subsp. *sporocinereus*; *Streptomyces sporocinereus*	1014 5487	72.0	8375	Gp0144610	PRJDB3204	SAMD00040622	GCA_001570645	
Aeromonas encheleia CECT 4342	Esteve et al. 1995 emend. Huys et al. 1997	LMG 16330; CIP 104608; ATCC 51929; DSM 11577; NCTC 12917; S181	*Aeromonas encheleia*	4468 019	62.0	4045	Gp0122577	PRJEB7027	SAMEA2752421	GCA_000819825	

Strain	Authority	Other deposits	Synonyms	Base pairs	Percent G+C	No. proteins	Goldstamp	Bioproject accession	Biosample accession	Assembly accession	IMG OID
Streptomyces pluricolorescens JCM 4602	Okami and Umezawa 1961	BCRC 13657; CCRC 13657; IFM 1101; NRRL B-2121; NRRL ISP-5019; NCIMB 9813; CBS 550.68; ATCC 19798; DSM 40019; JCM 4302; IFO 12808; NBRC 12808; VKM Ac-765; NCIB 9813; RIA 1077; UNIQEM 184	*Streptomyces pluricolorescens*	7329 982	71.7	6782		PRJDB10510	SAMD00245484	GCA_014650395	
Aeromonas hydrophila subsp. *ranae* CIP 107985	Huys et al. 2003	LMG 19707; CCUG 46211; DSM 17695; Au-1D12	*Aeromonas hydrophila* subsp. *ranae*	4681 175	61.6	4279	Gp0122583	PRJEB7049	SAMEA2752399	GCA_000820325	

Strain	Authority	Other deposits	Synonyms	Base pairs	Percent G+C	No. proteins	Goldstamp	Bioproject accession	Biosample accession	Assembly accession	IMG OID
Aeromonas hydrophila ATCC 7966	(Chester 1901) Stanier 1943 emend. Beaz-Hidalgo et al. 2013	LMG 12156; LMG 13439; LMG 2844; BCRC 13018; BCRC (formerly CCRC) 13018; CCRC 13018; CIP 76.14; NCIMB 9240; CCUG 14551; DSM 30187; JCM 1027; NBIMCC 8962; NCTC 8049; CCM 7232; CDC 359-60; HAMBI 1847; HAMBI 1973; IAM 12460; NCIB 9240	*Aeromonas hydrophila*; *Aeromonas hydrophila* subsp. *hydrophila*; *Bacillus hydrophilus*	4744 448	61.5	4122	Gp0000262	PRJNA16697	SAMN02604052	GCA_000014805	639633004
Tepidiforma thermophila G233T	Palmer et al. 2023	CGMCC 1.13589; KCTC 52669	*Tepidiforma thermophila*	2743 337	69.4	2627	Gp0112437	PRJNA323209	SAMN05216314	GCA_002563855	
bin.58.fa				3216 994	60.9	3380					

Methods, Results and References

The genome sequence data were uploaded to the Type (Strain) Genome Server (TYGS), a free bioinformatics platform available under https://tygs.dsmz.de, for a whole genome-based taxonomic analysis [1]. The analysis also made use of recently introduced methodological updates and features [2]. Information on nomenclature, synonymy and associated taxonomic literature was provided by TYGS's sister database, the List of Prokaryotic names with Standing in Nomenclature (LPSN, available at https://lpsn.dsmz.de) [2]. The results were provided by the TYGS on 2024-06-16. The TYGS analysis was subdivided into the following steps:

Determination of closely related type strains

The determination of closely related type strains did not succeed because not a single 16S rDNA gene sequence was detected in the provided user genomes. The subsequent analyses are thus only based on the provided genome data and the manually selected type strains, if any.

Pairwise comparison of genome sequences

For the phylogenomic inference, all pairwise comparisons among the set of genomes were conducted using GBDP and accurate intergenomic distances inferred under the algorithm 'trimming' and distance formula d_5 [3]. 100 distance replicates were calculated each. Digital DDH values and confidence intervals were calculated using the recommended settings of the GGDC 4.0 [2,3].

Phylogenetic inference

The resulting intergenomic distances were used to infer a balanced minimum evolution tree with branch support via FASTME 2.1.6.1 including SPR postprocessing [4]. Branch support was inferred from 100 pseudo-bootstrap replicates each. The trees were rooted at the midpoint [5] and visualized with PhyD3 [6].

Type-based species and subspecies clustering

The type-based species clustering using a 70% dDDH radius around each of the 10 type strains was done as previously described [1]. The resulting groups are shown in Table 1 and 4. Subspecies clustering was done using a 79% dDDH threshold as previously introduced [7].

Results

Type-based species and subspecies clustering

The resulting species and subspecies clusters are listed in Table 4, whereas the taxonomic identification of the query strains is found in Table 1. Briefly, the clustering yielded 9 species clusters and the provided query strains were assigned to 1 of these. Moreover, user strains were located in 1 of 10 subspecies clusters.

Figure caption genome tree

Figure 2. Tree inferred with FastME 2.1.6.1 [4] from GBDP distances calculated from genome sequences. The branch lengths are scaled in terms of GBDP distance formula d_5. The numbers above branches are GBDP pseudo-bootstrap support values > 60 % from 100 replications, with an average branch support of 85.6 %. The tree was rooted at the midpoint [5].

References

[1] Meier-Kolthoff JP, Göker M. TYGS is an automated high-throughput platform for state-of-the-art genome-based taxonomy. Nat. Commun. 2019;10: 2182. DOI: 10.1038/s41467-019-10210-3

[2] Meier-Kolthoff JP, Sardà Carbasse J, Peinado-Olarte RL, Göker M. TYGS and LPSN: a database tandem for fast and reliable genome-based classification and nomenclature of prokaryotes. Nucleic Acid Res. 2022;50: D801–D807. DOI: 10.1093/nar/gkab902

[3] Meier-Kolthoff JP, Auch AF, Klenk H-P, Göker M. Genome sequence-based species delimitation with confidence intervals and improved distance functions. BMC Bioinformatics. 2013;14: 60. DOI: 10.1186/1471-2105-14-60

[4] Lefort V, Desper R, Gascuel O. FastME 2.0: A comprehensive, accurate, and fast distance-based phylogeny inference program. Mol Biol Evol. 2015;32: 2798–2800. DOI: 10.1093/molbev/msv150

[5] Farris JS. Estimating phylogenetic trees from distance matrices. Am Nat. 1972;106: 645–667.

[6] Kreft L, Botzki A, Coppens F, Vandepoele K, Van Bel M. PhyD3: A phylogenetic tree viewer with extended phyloXML support for functional genomics data visualization. Bioinformatics. 2017;33: 2946–2947. DOI: 10.1093/bioinformatics/btx324

[7] Meier-Kolthoff JP, Hahnke RL, Petersen J, Scheuner C, Michael V, Fiebig A, et al. Complete genome sequence of DSM 30083^T, the type strain (U5/41^T) of *Escherichia coli*, and a proposal for delineating subspecies in microbial taxonomy. Stand Genomic Sci. 2014;9: 2. DOI: 10.1186/1944-3277-9-2
