# Supplementary material for: Tracing active members in microbial communities by BONCAT and click chemistry-based enrichment of newly synthesized proteins
Source: ISME Commun. 2024 Dec 4;4(1):ycae153. doi: 10.1093/ismeco/ycae153 (PMC11683836; doi:10.1093/ismeco/ycae153)
Supplement: Genome_Server_ycae153 [file genome_server_ycae153.zip › Genome Server/Bin_5_TYGS_job_results.pdf]

TYPE (STRAIN) GENOME SERVER

JOB SUMMARY

PRINT DATE: 2024-06-17 08:45:42 +0200

JOB ID: 8ce6b427-b617-4191-b16f-6815bae704ea--01

RESULT PAGE: https://tygs.dsmz.de/user_results/show?guid=8ce6b427-b617-4191-b16f-6815bae704ea--01

Table 1: Phylogenies

Publication-ready versions of both the genome-scale GBDP tree and the 16S rRNA gene sequence tree can be customized and exported either in SVG (vector graphic) or PNG format from within the phylogeny viewers in your TYGS result page. For publications the **SVG format is recommended** because it is lossless, always keeps its high resolution and can also be easily converted to other popular formats such as PDF or EPS. Please follow the link provided above!

Table 2: Identification

The below list contains the result of the TYGS species identification routine.

Explanation of remarks that might occur in the below table:

remark [R1]: The TYGS type strain database is automatically updated on an almost daily basis. However, if a particular type strain genome is not available in the TYGS database, this can have several reasons which are detailed in the FAQ. You can request an extended 16S rRNA gene analysis via the 16S tree viewer found in your result page to detect **not yet genome-sequenced** type strains relevant for your study.

remark [R2]: > 70% dDDH value (formula d_4) and (almost) minimal dDDH values for gene-content formulae d_0 and d_6 indicate a potentially unreliable identification result and should thus be checked via the 16S rRNA gene sequence similarity. Such strong deviations can, in principle, be caused by sequence contamination.

remark [R3]: G+C content difference of > 1 % indicates a potentially unreliable identification result because within species G+C content varies no more than 1 %, if computed from genome sequences (PMID: 24505073).

Strain	Conclusion	Identification result	Remark
**'bin.5'**	belongs to known species	*Acidilutibacter cellobiosedens*	see [R2]<br>see [R3]

Table 3: Pairwise comparisons of user genomes vs. type-strain genomes

The following table contains the pairwise dDDH values between your user genomes and the selected type-strain genomes. The dDDH values are provided along with their confidence intervals (C.I.) for the three different GBDP formulas:

• formula d_0 (a.k.a. GGDC formula 1): length of all HSPs divided by total genome length
• formula d_4 (a.k.a. GGDC formula 2): sum of all identities found in HSPs divided by overall HSP length
• formula d_6 (a.k.a. GGDC formula 3): sum of all identities found in HSPs divided by total genome length

Note: Formula d_4 is independent of genome length and is thus robust against the use of incomplete draft genomes. For other reasons for preferring formula d_4, see the FAQ.

Query	Subject	d_0	C.I. d_0	d_4	C.I. d_4	d_6	C.I. d_6	Diff. G+C Percent
**'bin.5.fa'**	*Acidilutibacter cellobiosedens* JN-28	12.5	[9.8 - 15.8]	74.8	[71.8 - 77.6]	12.9	[10.6 - 15.7]	1.35
**'bin.5.fa'**	*Acetivibrio thermocellus* ATCC 27405	12.6	[9.9 - 15.8]	69.4	[66.4 - 72.3]	13.0	[10.6 - 15.7]	3.91
**'bin.5.fa'**	*Desulfofalx alkaliphila* DSM 12257	12.5	[9.9 - 15.8]	64.6	[61.7 - 67.4]	13.0	[10.6 - 15.7]	9.14
**'bin.5.fa'**	*Clostridium brassicae* ZC22-4	12.5	[9.9 - 15.8]	54.1	[51.4 - 56.8]	12.9	[10.6 - 15.7]	6.37
**'bin.5.fa'**	*Acetivibrio clariflavus* DSM 19732	12.6	[9.9 - 15.9]	47.6	[45.0 - 50.2]	13.0	[10.7 - 15.8]	0.64
**'bin.5.fa'**	*Gudongella oleilytica* W6	12.5	[9.9 - 15.8]	43.9	[41.3 - 46.4]	13.0	[10.6 - 15.7]	7.34
**'bin.5.fa'**	*Thermovorax subterraneus* DSM 21563	12.5	[9.8 - 15.7]	3.7	[2.8 - 4.8]	12.9	[10.6 - 15.6]	8.67
**'bin.5.fa'**	*Thermobrachium celere* DSM 8682	12.5	[9.8 - 15.7]	3.7	[2.8 - 4.8]	12.9	[10.6 - 15.6]	3.94
**'bin.5.fa'**	*Clostridium sardiniense* DSM 2632	12.5	[9.8 - 15.7]	3.7	[2.8 - 4.8]	12.9	[10.6 - 15.6]	6.66
**'bin.5.fa'**	*Streptobacillus canis* IHIT1603-19	12.5	[9.8 - 15.7]	3.7	[2.8 - 4.8]	12.9	[10.6 - 15.6]	8.55

Table 4: Strains in your dataset

Joint dataset of automatically determined closest type strains (if this mode was chosen), manually selected type strains (if selected accordingly) and the provided user strains, if provided (marked in yellow).

Strain	Authority	Other deposits	Synonyms	Base pairs	Percent G+C	No. proteins	Goldstamp	Bioproject accession	Biosample accession	Assembly accession	IMG OID
Clostridium sardiniense DSM 2632	Prévot 1938 emend. Wang et al. 2005	ATCC 33455; VPI 2971	*Clostridium sardiniense*	3927 989	28.4	3786	Gp0505729				2901040167
Clostridium brassicae ZC22-4	Wang et al. 2023	MCCC 1K07510; JCM 35370	*Clostridium brassicae*	4091 695	28.7	3744		PRJNA906960	SAMN31943988	GCA_026738875	
Gudongella oleilytica W6	Wu et al. 2020	CGMCC 1.5291; DSM 28124; CCAM 534	*Gudongella oleilytica*	2356 157	42.4	2236		PRJNA498902	SAMN10336855	GCA_004101785	
Desulfofalx alkaliphila DSM 12257	(Pikuta et al. 2000) Watanabe et al. 2021	ATCC 700784; VKM B-2192; S1	*Desulfofalx alkaliphila*; *Desulfohalotomaculum alkaliphilum*; *Desulfotomaculum alkaliphilum*	2620 599	44.2	2681	Gp0013893	PRJNA234958	SAMN02745670	GCA_000711975	2565956519
Acetivibrio thermocellus ATCC 27405	(Viljoen et al. 1926) Tindall 2019	LMG 10435; NRRL B-4536; DSM 1237; JCM 9322; NBRC 103400	*Acetivibrio thermocellus*; *Clostridium thermocellum*; *Hungateiclostridium thermocellum*; *Ruminiclostridium thermocellum*	3843 301	39.0	3173	Gp0000203	PRJNA314	SAMN02598481	GCA_000015865	640069309
Thermobrachium celere DSM 8682	Engle et al. 1996	ATCC 700318; JW/YL-NZ35; NZ35T	*Caloramator celer*; *Thermobrachium celere*	2413 383	31.1	2381	Gp0009733	PRJEA61471	SAMEA2271948	GCA_000430995	2519899721
Acidilutibacter cellobiosedens JN-28	Fan et al. 2023	JCM 39087; CCAM 418	*Acidilutibacter cellobiosedens*	3743 723	33.7	3578		PRJNA224116	SAMN10743633	GCF_004103715	
Thermovorax subterraneus DSM 21563	Mäkinen et al. 2012	70B; JCM 15541	*Thermovorax subterraneus*	2446 663	43.7	2431		PRJNA797675	SAMN25026613	GCA_021608145	

Strain	Authority	Other deposits	Synonyms	Base pairs	Percent G+C	No. proteins	Goldstamp	Bioproject accession	Biosample accession	Assembly accession	IMG OID
Streptobacillus canis IHIT1603-19	Eisenberg et al. 2020	CIP 111795; CCUG 74118; DSM 110501	*Streptobacillus canis*	1579950	26.5	1551		PRJNA591425	SAMN13381658	GCA_009733925	
Acetivibrio clariflavus DSM 19732	(Shiratori et al. 2009) Tindall 2019	NBRC 101661; EBR-02E-0045; EBR45	*Acetivibrio clariflavus*; *Clostridium clariflavum*; *Hungateiclostridium clariflavum*	4897678	35.7	3892	Gp0010603	PRJNA72805	SAMN02261428	GCA_000237085	2507262051
bin.5.fa				2546956	35.1	2611					

Methods, Results and References

The genome sequence data were uploaded to the Type (Strain) Genome Server (TYGS), a free bioinformatics platform available under https://tygs.dsmz.de, for a whole genome-based taxonomic analysis [1]. The analysis also made use of recently introduced methodological updates and features [2]. Information on nomenclature, synonymy and associated taxonomic literature was provided by TYGS's sister database, the List of Prokaryotic names with Standing in Nomenclature (LPSN, available at https://lpsn.dsmz.de) [2]. The results were provided by the TYGS on 2024-06-16. The TYGS analysis was subdivided into the following steps:

Determination of closely related type strains

The determination of closely related type strains did not succeed because not a single 16S rDNA gene sequence was detected in the provided user genomes. The subsequent analyses are thus only based on the provided genome data and the manually selected type strains, if any.

Pairwise comparison of genome sequences

For the phylogenomic inference, all pairwise comparisons among the set of genomes were conducted using GBDP and accurate intergenomic distances inferred under the algorithm 'trimming' and distance formula d_5 [3]. 100 distance replicates were calculated each. Digital DDH values and confidence intervals were calculated using the recommended settings of the GGDC 4.0 [2,3].

Phylogenetic inference

The resulting intergenomic distances were used to infer a balanced minimum evolution tree with branch support via FASTME 2.1.6.1 including SPR postprocessing [4]. Branch support was inferred from 100 pseudo-bootstrap replicates each. The trees were rooted at the midpoint [5] and visualized with PhyD3 [6].

Type-based species and subspecies clustering

The type-based species clustering using a 70% dDDH radius around each of the 10 type strains was done as previously described [1]. The resulting groups are shown in Table 1 and 4. Subspecies clustering was done using a 79% dDDH threshold as previously introduced [7].

Results

Type-based species and subspecies clustering

The resulting species and subspecies clusters are listed in Table 4, whereas the taxonomic identification of the query strains is found in Table 1. Briefly, the clustering yielded 10 species clusters and the provided query strains were assigned to 1 of these. Moreover, user strains were located in 1 of 11 subspecies clusters.

Figure caption genome tree

Figure 2. Tree inferred with FastME 2.1.6.1 [4] from GBDP distances calculated from genome sequences. The branch lengths are scaled in terms of GBDP distance formula d_5. The numbers above branches are GBDP pseudo-bootstrap support values > 60 % from 100 replications, with an average branch support of 48.9 %. The tree was rooted at the midpoint [5].

References

[1] Meier-Kolthoff JP, Göker M. TYGS is an automated high-throughput platform for state-of-the-art genome-based taxonomy. Nat. Commun. 2019;10: 2182. DOI: 10.1038/s41467-019-10210-3

[2] Meier-Kolthoff JP, Sardà Carbasse J, Peinado-Olarte RL, Göker M. TYGS and LPSN: a database tandem for fast and reliable genome-based classification and nomenclature of prokaryotes. Nucleic Acid Res. 2022;50: D801–D807. DOI: 10.1093/nar/gkab902

[3] Meier-Kolthoff JP, Auch AF, Klenk H-P, Göker M. Genome sequence-based species delimitation with confidence intervals and improved distance functions. BMC Bioinformatics. 2013;14: 60. DOI: 10.1186/1471-2105-14-60

[4] Lefort V, Desper R, Gascuel O. FastME 2.0: A comprehensive, accurate, and fast distance-based phylogeny inference program. Mol Biol Evol. 2015;32: 2798–2800. DOI: 10.1093/molbev/msv150

[5] Farris JS. Estimating phylogenetic trees from distance matrices. Am Nat. 1972;106: 645–667.

[6] Kreft L, Botzki A, Coppens F, Vandepoele K, Van Bel M. PhyD3: A phylogenetic tree viewer with extended phyloXML support for functional genomics data visualization. Bioinformatics. 2017;33: 2946–2947. DOI: 10.1093/bioinformatics/btx324

[7] Meier-Kolthoff JP, Hahnke RL, Petersen J, Scheuner C, Michael V, Fiebig A, et al. Complete genome sequence of DSM 30083^{T}, the type strain (U5/41^{T}) of *Escherichia coli*, and a proposal for delineating subspecies in microbial taxonomy. Stand Genomic Sci. 2014;9: 2. DOI: 10.1186/1944-3277-9-2
