# Supplementary material for: Tracing active members in microbial communities by BONCAT and click chemistry-based enrichment of newly synthesized proteins
Source: ISME Commun. 2024 Dec 4;4(1):ycae153. doi: 10.1093/ismeco/ycae153 (PMC11683836; doi:10.1093/ismeco/ycae153)
Supplement: Genome_Server_ycae153 [file genome_server_ycae153.zip › Genome Server/Bin_66_TYGS_job_results.pdf]

TYPE (STRAIN) GENOME SERVER

JOB SUMMARY

PRINT DATE:	2024-06-17 09:27:47 +0200
JOB ID:	8ce6b427-b617-4191-b16f-6815bae704ea--21
RESULT PAGE:	https://tygs.dsmz.de/user_results/show?guid=8ce6b427-b617-4191-b16f-6815bae704ea--21

Table 1: Phylogenies

Publication-ready versions of both the genome-scale GBDP tree and the 16S rRNA gene sequence tree can be customized and exported either in SVG (vector graphic) or PNG format from within the phylogeny viewers in your TYGS result page. For publications the **SVG format is recommended** because it is lossless, always keeps its high resolution and can also be easily converted to other popular formats such as PDF or EPS. Please follow the link provided above!

Table 2: Identification

The below list contains the result of the TYGS species identification routine.

Explanation of remarks that might occur in the below table:

remark [R1]: The TYGS type strain database is automatically updated on an almost daily basis. However, if a particular type strain genome is not available in the TYGS database, this can have several reasons which are detailed in the FAQ. You can request an extended 16S rRNA gene analysis via the 16S tree viewer found in your result page to detect **not yet genome-sequenced** type strains relevant for your study.

remark [R2]: > 70% dDDH value (formula d_4) and (almost) minimal dDDH values for gene-content formulae d_0 and d_6 indicate a potentially unreliable identification result and should thus be checked via the 16S rRNA gene sequence similarity. Such strong deviations can, in principle, be caused by sequence contamination.

remark [R3]: G+C content difference of > 1 % indicates a potentially unreliable identification result because within species G+C content varies no more than 1 %, if computed from genome sequences (PMID: 24505073).

Strain	Conclusion	Identification result	Remark
**'bin.66'**	potential new species		see [R1]

Table 3: Pairwise comparisons of user genomes vs. type-strain genomes

The following table contains the pairwise dDDH values between your user genomes and the selected type-strain genomes. The dDDH values are provided along with their confidence intervals (C.I.) for the three different GBDP formulas:

- formula d_0 (a.k.a. GGDC formula 1): length of all HSPs divided by total genome length
- formula d_4 (a.k.a. GGDC formula 2): sum of all identities found in HSPs divided by overall HSP length
- formula d_6 (a.k.a. GGDC formula 3): sum of all identities found in HSPs divided by total genome length

Note: Formula d_4 is independent of genome length and is thus robust against the use of incomplete draft genomes. For other reasons for preferring formula d_4, see the FAQ.

Query	Subject	d_0	C.I. d_0	d_4	C.I. d_4	d_6	C.I. d_6	Diff. G+C Percent
**'bin.66.fa'**	*Brevundimonas vesicularis* NBRC 12165	12.5	[9.8 - 15.7]	25.8	[23.4 - 28.3]	12.9	[10.6 - 15.6]	1.27
**'bin.66.fa'**	*Baekduia alba* 0141_2T	12.5	[9.8 - 15.7]	24.2	[21.9 - 26.6]	12.9	[10.6 - 15.6]	7.81
**'bin.66.fa'**	*Roseomonas stagni* DSM 19981	12.5	[9.8 - 15.7]	20.0	[17.8 - 22.4]	12.9	[10.6 - 15.6]	5.65
**'bin.66.fa'**	*Acidovorax oryzae* ATCC 19882	12.5	[9.8 - 15.8]	17.3	[15.1 - 19.6]	12.9	[10.6 - 15.6]	3.69
**'bin.66.fa'**	*Candidatus* Evtepia excrementipullorum ChiSjej3B21-3892	12.5	[9.8 - 15.8]	17.3	[15.2 - 19.6]	12.9	[10.6 - 15.7]	2.44
**'bin.66.fa'**	*Marihabitans asiaticum* DSM 18935	12.5	[9.8 - 15.8]	15.4	[13.4 - 17.7]	12.9	[10.6 - 15.6]	6.03
**'bin.66.fa'**	*Stigmatella erecta* DSM 16858	12.5	[9.8 - 15.7]	15.0	[13.0 - 17.2]	12.9	[10.6 - 15.6]	4.35
**'bin.66.fa'**	*Candidatus* Evtepia faecigallinarum ChiHcec3-601	12.5	[9.8 - 15.8]	14.8	[12.8 - 17.0]	12.9	[10.6 - 15.6]	1.46
**'bin.66.fa'**	*Novosphingobium capsulatum* NBRC 12533	12.5	[9.8 - 15.7]	13.9	[12.0 - 16.1]	12.9	[10.6 - 15.6]	0.66
**'bin.66.fa'**	*Streptomyces montanus* NEAU-C151	12.5	[9.8 - 15.7]	3.7	[2.8 - 4.8]	12.9	[10.6 - 15.6]	4.81

Table 4: Strains in your dataset

Joint dataset of automatically determined closest type strains (if this mode was chosen), manually selected type strains (if selected accordingly) and the provided user strains, if provided (marked in yellow).

Strain	Authority	Other deposits	Synonyms	Base pairs	Percent G+C	No. proteins	Goldstamp	Bioproject accession	Biosample accession	Assembly accession	IMG OID
Marihabitans asiaticum DSM 18935	Kageyama et al. 2008	JCM 16543; HG667; MBIC 7497	*Marihabitans asiaticum*	2953873	71.1	2783	Gp0304930	PRJNA538389	SAMN11512784	GCA_007828725	2816332116
Candidatus Evtepia faecigallinarum ChiHcec3-601	Gilroy et al. 2021		*Candidatus* Evtepia faecigallinarum	2322393	63.6	2250		PRJNA543206	SAMN15816724	GCA_019115225	
Candidatus Evtepia excrementipullorum ChiSjej3B21-3892	Gilroy et al. 2021		*Candidatus* Evtepia excrementipullorum	2108333	62.6	2079		PRJNA543206	SAMN15816827	GCA_019118615	
Streptomyces montanus NEAU-C151	Jiang et al. 2020	CGMCC 4.7498; DSM 107808	*Streptomyces montanus*	10219292	69.9	8758		PRJNA542769	SAMN11638123	GCA_005786655	
Acidovorax oryzae ATCC 19882	Schaad et al. 2009	LMG 10904; LMG 1806; NCPPB 1392; ICMP 3960; FC-143; ICPB 30003	*Acidovorax oryzae*; *Paracidovorax oryzae*	5527201	68.7	4874	Gp0044038	PRJNA223028	SAMN02743377	GCA_000687165	2563366540
Roseomonas stagni DSM 19981	Furuhata et al. 2008 emend. Hördt et al. 2020	KCTC 22213; JCM 15034; HS-69	*Falsiroseomonas stagni*; *Roseomonas stagni*	6377797	70.7	5924	Gp0102854	PRJNA262325	SAMN02745775	GCA_900114315	2602042029

Strain	Authority	Other deposits	Synonyms	Base pairs	Percent G+C	No. proteins	Goldstamp	Bioproject accession	Biosample accession	Assembly accession	IMG OID
Brevundimonas vesicularis NBRC 12165	(Büsing et al. 1953) Segers et al. 1994 emend. Hördt et al. 2020	CFBP 3032; LMG 2350; BCRC 11012; CCRC 11012; CIP 101035; NCIMB 1945; NCCB 76049; ATCC 11426; CCUG 2032; CCUG 28332; CCUG 28333; DSM 7226; DSM 7233; JCM 1477; IFO 12165; NCTC 10900; CCM 3398; VKM B-974; IAM 12105; NCIB 1945; NCMB 1945	*Brevundimonas vesicularis*; *Corynebacterium vesiculare*; *Pseudomonas vesicularis*	3358429	66.3	3264	Gp0075761	PRJDB1343	SAMD00047217	GCA_001592205	
Novosphingobium capsulatum NBRC 12533	(Leifson 1962) Takeuchi et al. 2001 emend. Hördt et al. 2020	LMG 2830; CIP 82.103; ATCC 14666; CCUG 17697; CCUG 31202; DSM 30196; JCM 7452; JCM 7508; IFO 12533; VKM B-1564; GIFU 11526; HAMBI 103	*Flavobacterium capsulatum*; *Novosphingobium capsulatum*; *Sphingomonas capsulata*	4836455	65.7	4299	Gp0023611	PRJDB321	SAMD00046742	GCA_001598375	
Stigmatella erecta DSM 16858	(Schroeter 1886) McCurdy 1971	ATCC 25191	*Cystobacter erectus*; *Stigmatella erecta*	9217225	69.4	7394	Gp0148787	PRJNA335186	SAMN05443639	GCA_900111745	2693429888

Strain	Authority	Other deposits	Synonyms	Base pairs	Percent G+C	No. proteins	Goldstamp	Bioproject accession	Biosample accession	Assembly accession	IMG OID
Baekduia alba 0141_2T	Vieira et al. 2023	LMG 30000; CECT 9239; DSM 104299	*Baekduia alba*	5863246	72.9	5762		PRJNA904972	SAMN31859443	GCA_028416635	
bin.66.fa				3301666	65.1	3108					

Methods, Results and References

The genome sequence data were uploaded to the Type (Strain) Genome Server (TYGS), a free bioinformatics platform available under https://tygs.dsmz.de, for a whole genome-based taxonomic analysis [1]. The analysis also made use of recently introduced methodological updates and features [2]. Information on nomenclature, synonymy and associated taxonomic literature was provided by TYGS's sister database, the List of Prokaryotic names with Standing in Nomenclature (LPSN, available at https://lpsn.dsmz.de) [2]. The results were provided by the TYGS on 2024-06-16. The TYGS analysis was subdivided into the following steps:

Determination of closely related type strains

The determination of closely related type strains did not succeed because not a single 16S rDNA gene sequence was detected in the provided user genomes. The subsequent analyses are thus only based on the provided genome data and the manually selected type strains, if any.

Pairwise comparison of genome sequences

For the phylogenomic inference, all pairwise comparisons among the set of genomes were conducted using GBDP and accurate intergenomic distances inferred under the algorithm 'trimming' and distance formula d_5 [3]. 100 distance replicates were calculated each. Digital DDH values and confidence intervals were calculated using the recommended settings of the GGDC 4.0 [2,3].

Phylogenetic inference

The resulting intergenomic distances were used to infer a balanced minimum evolution tree with branch support via FASTME 2.1.6.1 including SPR postprocessing [4]. Branch support was inferred from 100 pseudo-bootstrap replicates each. The trees were rooted at the midpoint [5] and visualized with PhyD3 [6].

Type-based species and subspecies clustering

The type-based species clustering using a 70% dDDH radius around each of the 10 type strains was done as previously described [1]. The resulting groups are shown in Table 1 and 4. Subspecies clustering was done using a 79% dDDH threshold as previously introduced [7].

Results

Type-based species and subspecies clustering

The resulting species and subspecies clusters are listed in Table 4, whereas the taxonomic identification of the query strains is found in Table 1. Briefly, the clustering yielded 11 species clusters and the provided query strains were assigned to 1 of these. Moreover, user strains were located in 1 of 11 subspecies clusters.

Figure caption genome tree

Figure 2. Tree inferred with FastME 2.1.6.1 [4] from GBDP distances calculated from genome sequences. The branch lengths are scaled in terms of GBDP distance formula d_5. The numbers above branches are GBDP pseudo-bootstrap support values > 60 % from 100 replications, with an average branch support of 41.4 %. The tree was rooted at the midpoint [5].

References

[1] Meier-Kolthoff JP, Göker M. TYGS is an automated high-throughput platform for state-of-the-art genome-based taxonomy. Nat. Commun. 2019;10: 2182. DOI: 10.1038/s41467-019-10210-3

[2] Meier-Kolthoff JP, Sardà Carbasse J, Peinado-Olarte RL, Göker M. TYGS and LPSN: a database tandem for fast and reliable genome-based classification and nomenclature of prokaryotes. Nucleic Acid Res. 2022;50: D801–D807. DOI: 10.1093/nar/gkab902

[3] Meier-Kolthoff JP, Auch AF, Klenk H-P, Göker M. Genome sequence-based species delimitation with confidence intervals and improved distance functions. BMC Bioinformatics. 2013;14: 60. DOI: 10.1186/1471-2105-14-60

[4] Lefort V, Desper R, Gascuel O. FastME 2.0: A comprehensive, accurate, and fast distance-based phylogeny inference program. Mol Biol Evol. 2015;32: 2798–2800. DOI: 10.1093/molbev/msv150

[5] Farris JS. Estimating phylogenetic trees from distance matrices. Am Nat. 1972;106: 645–667.

[6] Kreft L, Botzki A, Coppens F, Vandepoele K, Van Bel M. PhyD3: A phylogenetic tree viewer with extended phyloXML support for functional genomics data visualization. Bioinformatics. 2017;33: 2946–2947. DOI: 10.1093/bioinformatics/btx324

[7] Meier-Kolthoff JP, Hahnke RL, Petersen J, Scheuner C, Michael V, Fiebig A, et al. Complete genome sequence of DSM 30083^T, the type strain (U5/41^T) of *Escherichia coli*, and a proposal for delineating subspecies in microbial taxonomy. Stand Genomic Sci. 2014;9: 2. DOI: 10.1186/1944-3277-9-2
