# Supplementary material for: Tracing active members in microbial communities by BONCAT and click chemistry-based enrichment of newly synthesized proteins
Source: ISME Commun. 2024 Dec 4;4(1):ycae153. doi: 10.1093/ismeco/ycae153 (PMC11683836; doi:10.1093/ismeco/ycae153)
Supplement: Genome_Server_ycae153 [file genome_server_ycae153.zip › Genome Server/Bin_68_TYGS_job_results.pdf]

TYPE (STRAIN) GENOME SERVER

JOB SUMMARY

PRINT DATE:	2024-06-17 09:28:54 +0200
JOB ID:	8ce6b427-b617-4191-b16f-6815bae704ea--23
RESULT PAGE:	https://tygs.dsmz.de/user_results/show?guid=8ce6b427-b617-4191-b16f-6815bae704ea--23

Table 1: Phylogenies

Publication-ready versions of both the genome-scale GBDP tree and the 16S rRNA gene sequence tree can be customized and exported either in SVG (vector graphic) or PNG format from within the phylogeny viewers in your TYGS result page. For publications the **SVG format is recommended** because it is lossless, always keeps its high resolution and can also be easily converted to other popular formats such as PDF or EPS. Please follow the link provided above!

Table 2: Identification

The below list contains the result of the TYGS species identification routine.

Explanation of remarks that might occur in the below table:

remark [R1]: The TYGS type strain database is automatically updated on an almost daily basis. However, if a particular type strain genome is not available in the TYGS database, this can have several reasons which are detailed in the FAQ. You can request an extended 16S rRNA gene analysis via the 16S tree viewer found in your result page to detect **not yet genome-sequenced** type strains relevant for your study.

remark [R2]: > 70% dDDH value (formula d_4) and (almost) minimal dDDH values for gene-content formulae d_0 and d_6 indicate a potentially unreliable identification result and should thus be checked via the 16S rRNA gene sequence similarity. Such strong deviations can, in principle, be caused by sequence contamination.

remark [R3]: G+C content difference of > 1 % indicates a potentially unreliable identification result because within species G+C content varies no more than 1 %, if computed from genome sequences (PMID: 24505073).

Strain	Conclusion	Identification result	Remark
**'bin.68'**	belongs to known species	*Candidatus* Syntrophosphaera thermopropionivorans	see [R2]<br>see [R3]

Table 3: Pairwise comparisons of user genomes vs. type-strain genomes

The following table contains the pairwise dDDH values between your user genomes and the selected type-strain genomes. The dDDH values are provided along with their confidence intervals (C.I.) for the three different GBDP formulas:

- formula d_0 (a.k.a. GGDC formula 1): length of all HSPs divided by total genome length
- formula d_4 (a.k.a. GGDC formula 2): sum of all identities found in HSPs divided by overall HSP length
- formula d_6 (a.k.a. GGDC formula 3): sum of all identities found in HSPs divided by total genome length

Note: Formula d_4 is independent of genome length and is thus robust against the use of incomplete draft genomes. For other reasons for preferring formula d_4, see the FAQ.

Query	Subject	d_0	C.I. d_0	d_4	C.I. d_4	d_6	C.I. d_6	Diff. G+C Percent
**'bin.68.fa'**	*Candidatus* Syntrophosphaera thermopropionivorans W5	12.5	[9.9 - 15.8]	88.0	[85.5 - 90.1]	13.0	[10.6 - 15.7]	4.99
**'bin.68.fa'**	*Acetomicrobium flavidum* DSM 20664	12.5	[9.8 - 15.7]	64.7	[61.7 - 67.5]	12.9	[10.6 - 15.6]	6.67
**'bin.68.fa'**	*Desulfofundulus luciae* DSM 12396	12.5	[9.8 - 15.7]	41.3	[38.8 - 43.8]	12.9	[10.6 - 15.6]	13.57
**'bin.68.fa'**	*Anaerosolibacter carboniphilus* DSM 103526	12.5	[9.8 - 15.7]	3.7	[2.8 - 4.8]	12.9	[10.6 - 15.6]	3.69
**'bin.68.fa'**	*Ohtaekwangia koreensis* DSM 25262	12.5	[9.8 - 15.7]	3.7	[2.8 - 4.8]	12.9	[10.6 - 15.6]	0.31
**'bin.68.fa'**	*Desulfofundulus salinum* 435	12.5	[9.8 - 15.7]	3.7	[2.8 - 4.8]	12.9	[10.6 - 15.6]	13.47
**'bin.68.fa'**	*Dictyobacter formicarum* BCRC 81204T	12.5	[9.8 - 15.7]	3.7	[2.8 - 4.8]	12.9	[10.6 - 15.6]	9.49
**'bin.68.fa'**	*Anaerobium acetethylicum* DSM 29698	12.5	[9.8 - 15.7]	3.7	[2.8 - 4.8]	12.9	[10.6 - 15.6]	1.92
**'bin.68.fa'**	*Christiangramia forsetii* CGMCC 1.15422	12.5	[9.8 - 15.7]	3.7	[2.8 - 4.8]	12.9	[10.6 - 15.6]	5.09
**'bin.68.fa'**	*Christiangramia forsetii* KT0803	12.5	[9.8 - 15.7]	3.7	[2.8 - 4.8]	12.9	[10.6 - 15.6]	5.0

Table 4: Strains in your dataset

Joint dataset of automatically determined closest type strains (if this mode was chosen), manually selected type strains (if selected accordingly) and the provided user strains, if provided (marked in yellow).

Strain	Authority	Other deposits	Synonyms	Base pairs	Percent G+C	No. proteins	Goldstamp	Bioproject accession	Biosample accession	Assembly accession	IMG OID
Dictyobacter formicarum BCRC 81204T	Yabe et al. 2021	CGMCC 1.17206; SOSP1-9	*Dictyobacter formicarum*	9218854	51.1	7534		PRJNA224116	SAMD00251983	GCF_016587435	
Desulfofundulus luciae DSM 12396	(Liu et al. 1997) Watanabe et al. 2018	ATCC 700428; SLT; SMCC W644	*Desulfofundulus luciae*; *Desulfotomaculum luciae*	2941140	55.2	2983	Gp0505783				2926666083
Anaerosolibacter carboniphilus DSM 103526	Hong et al. 2015	KCTC 15396; JCM 19988; IRF19	*Anaerosolibacter carboniphilus*	5175869	37.9	5125	Gp0456343				2861187593
Ohtaekwangia koreensis DSM 25262	Yoon et al. 2011	3B-2; KCTC 23018; CCUG 58939	*Ohtaekwangia koreensis*	7235705	41.9	5891	Gp0103669	PRJNA262292	SAMN05660236	GCA_900167975	2595698225
Christiangramia forsetii KT0803	(Panschin et al. 2017) Deshmukh and Oren 2023	CGMCC 1.15422; DSM 17595; KT0803	*Christiangramia forsetii*; *Gramella forsetii*	3798465	36.6	3584	Gp0000261	PRJEA19061	SAMEA2272667	GCA_000060345	639633025
Christiangramia forsetii CGMCC 1.15422	(Panschin et al. 2017) Deshmukh and Oren 2023	CGMCC 1.15422; DSM 17595; KT0803	*Christiangramia forsetii*; *Gramella forsetii*	3746569	36.5	3349		PRJDB10509	SAMD00245059	GCA_014642775	
Anaerobium acetethylicum DSM 29698	Patil et al. 2015	LMG 28619; KCTC 15450; GluBS11	*Anaerobium acetethylicum*	4584081	43.5	3986	Gp0139288	PRJEB15475	SAMN05421730	GCA_900096945	
Acetomicrobium flavidum DSM 20664	Soutschek et al. 1985	LMG 6941; ATCC 43122	*Acetomicrobium flavidum*	2021699	48.3	1944	Gp0148757	PRJNA331522	SAMN05444368	GCA_900129645	2695420966
Candidatus Syntrophosphaera thermopropionivorans W5	Dyksma and Gallert 2019	Cloacimonetes bacterium	*Candidatus* Syntrophosphaera thermopropionivorans	1749786	36.6	1372		PRJNA525756	SAMN11044455	GCA_004353895	

Strain	Authority	Other deposits	Synonyms	Base pairs	Percent G+C	No. proteins	Goldstamp	Bioproject accession	Biosample accession	Assembly accession	IMG OID
Desulfofundulus salinum 435	Grouzdev et al. 2018	DSM 23196; VKM B-1492	*Desulfofundulus salinum*; *Desulfotomaculum nigrificans* subsp. *salinus*; *Desulfotomaculum salinum*	2880 898	55.1	2755		PRJNA494082	SAMN10148148	GCA_003627965	
bin.68.fa				2146 380	41.6	2137					

Methods, Results and References

The genome sequence data were uploaded to the Type (Strain) Genome Server (TYGS), a free bioinformatics platform available under https://tygs.dsmz.de, for a whole genome-based taxonomic analysis [1]. The analysis also made use of recently introduced methodological updates and features [2]. Information on nomenclature, synonymy and associated taxonomic literature was provided by TYGS's sister database, the List of Prokaryotic names with Standing in Nomenclature (LPSN, available at https://lpsn.dsmz.de) [2]. The results were provided by the TYGS on 2024-06-16. The TYGS analysis was subdivided into the following steps:

Determination of closely related type strains

The determination of closely related type strains did not succeed because not a single 16S rDNA gene sequence was detected in the provided user genomes. The subsequent analyses are thus only based on the provided genome data and the manually selected type strains, if any.

Pairwise comparison of genome sequences

For the phylogenomic inference, all pairwise comparisons among the set of genomes were conducted using GBDP and accurate intergenomic distances inferred under the algorithm 'trimming' and distance formula d_5 [3]. 100 distance replicates were calculated each. Digital DDH values and confidence intervals were calculated using the recommended settings of the GGDC 4.0 [2,3].

Phylogenetic inference

The resulting intergenomic distances were used to infer a balanced minimum evolution tree with branch support via FASTME 2.1.6.1 including SPR postprocessing [4]. Branch support was inferred from 100 pseudo-bootstrap replicates each. The trees were rooted at the midpoint [5] and visualized with PhyD3 [6].

Type-based species and subspecies clustering

The type-based species clustering using a 70% dDDH radius around each of the 10 type strains was done as previously described [1]. The resulting groups are shown in Table 1 and 4. Subspecies clustering was done using a 79% dDDH threshold as previously introduced [7].

Results

Type-based species and subspecies clustering

The resulting species and subspecies clusters are listed in Table 4, whereas the taxonomic identification of the query strains is found in Table 1. Briefly, the clustering yielded 9 species clusters and the provided query strains were assigned to 1 of these. Moreover, user strains were located in 1 of 9 subspecies clusters.

Figure caption genome tree

Figure 2. Tree inferred with FastME 2.1.6.1 [4] from GBDP distances calculated from genome sequences. The branch lengths are scaled in terms of GBDP distance formula d_5. The numbers above branches are GBDP pseudo-bootstrap support values > 60 % from 100 replications, with an average branch support of 82.1 %. The tree was rooted at the midpoint [5].

References

[1] Meier-Kolthoff JP, Göker M. TYGS is an automated high-throughput platform for state-of-the-art genome-based taxonomy. Nat. Commun. 2019;10: 2182. DOI: 10.1038/s41467-019-10210-3

[2] Meier-Kolthoff JP, Sardà Carbasse J, Peinado-Olarte RL, Göker M. TYGS and LPSN: a database tandem for fast and reliable genome-based classification and nomenclature of prokaryotes. Nucleic Acid Res. 2022;50: D801–D807. DOI: 10.1093/nar/gkab902

[3] Meier-Kolthoff JP, Auch AF, Klenk H-P, Göker M. Genome sequence-based species delimitation with confidence intervals and improved distance functions. BMC Bioinformatics. 2013;14: 60. DOI: 10.1186/1471-2105-14-60

[4] Lefort V, Desper R, Gascuel O. FastME 2.0: A comprehensive, accurate, and fast distance-based phylogeny inference program. Mol Biol Evol. 2015;32: 2798–2800. DOI: 10.1093/molbev/msv150

[5] Farris JS. Estimating phylogenetic trees from distance matrices. Am Nat. 1972;106: 645–667.

[6] Kreft L, Botzki A, Coppens F, Vandepoele K, Van Bel M. PhyD3: A phylogenetic tree viewer with extended phyloXML support for functional genomics data visualization. Bioinformatics. 2017;33: 2946–2947. DOI: 10.1093/bioinformatics/btx324

[7] Meier-Kolthoff JP, Hahnke RL, Petersen J, Scheuner C, Michael V, Fiebig A, et al. Complete genome sequence of DSM 30083^{T}, the type strain (U5/41^{T}) of *Escherichia coli*, and a proposal for delineating subspecies in microbial taxonomy. Stand Genomic Sci. 2014;9: 2. DOI: 10.1186/1944-3277-9-2
