# Supplementary material for: Tracing active members in microbial communities by BONCAT and click chemistry-based enrichment of newly synthesized proteins
Source: ISME Commun. 2024 Dec 4;4(1):ycae153. doi: 10.1093/ismeco/ycae153 (PMC11683836; doi:10.1093/ismeco/ycae153)
Supplement: Genome_Server_ycae153 [file genome_server_ycae153.zip › Genome Server/Bin_69_TYGS_job_results.pdf]

TYPE (STRAIN) GENOME SERVER

JOB SUMMARY

PRINT DATE:	2024-06-17 09:25:40 +0200
JOB ID:	8ce6b427-b617-4191-b16f-6815bae704ea--24
RESULT PAGE:	https://tygs.dsmz.de/user_results/show?guid=8ce6b427-b617-4191-b16f-6815bae704ea--24

Table 1: Phylogenies

Publication-ready versions of both the genome-scale GBDP tree and the 16S rRNA gene sequence tree can be customized and exported either in SVG (vector graphic) or PNG format from within the phylogeny viewers in your TYGS result page. For publications the **SVG format is recommended** because it is lossless, always keeps its high resolution and can also be easily converted to other popular formats such as PDF or EPS. Please follow the link provided above!

Table 2: Identification

The below list contains the result of the TYGS species identification routine.

Explanation of remarks that might occur in the below table:

remark [R1]: The TYGS type strain database is automatically updated on an almost daily basis. However, if a particular type strain genome is not available in the TYGS database, this can have several reasons which are detailed in the FAQ. You can request an extended 16S rRNA gene analysis via the 16S tree viewer found in your result page to detect **not yet genome-sequenced** type strains relevant for your study.

remark [R2]: > 70% dDDH value (formula d_4) and (almost) minimal dDDH values for gene-content formulae d_0 and d_6 indicate a potentially unreliable identification result and should thus be checked via the 16S rRNA gene sequence similarity. Such strong deviations can, in principle, be caused by sequence contamination.

remark [R3]: G+C content difference of > 1 % indicates a potentially unreliable identification result because within species G+C content varies no more than 1 %, if computed from genome sequences (PMID: 24505073).

Strain	Conclusion	Identification result	Remark
**'bin.69'**	potential new species		see [R1]

Table 3: Pairwise comparisons of user genomes vs. type-strain genomes

The following table contains the pairwise dDDH values between your user genomes and the selected type-strain genomes. The dDDH values are provided along with their confidence intervals (C.I.) for the three different GBDP formulas:

- formula d_0 (a.k.a. GGDC formula 1): length of all HSPs divided by total genome length
- formula d_4 (a.k.a. GGDC formula 2): sum of all identities found in HSPs divided by overall HSP length
- formula d_6 (a.k.a. GGDC formula 3): sum of all identities found in HSPs divided by total genome length

Note: Formula d_4 is independent of genome length and is thus robust against the use of incomplete draft genomes. For other reasons for preferring formula d_4, see the FAQ.

Query	Subject	d_0	C.I. d_0	d_4	C.I. d_4	d_6	C.I. d_6	Diff. G+C Percent
**'bin.69.fa'**	*Autumnicola patrickiae* F188	12.5	[9.8 - 15.7]	24.8	[22.4 - 27.2]	12.9	[10.6 - 15.6]	7.44
**'bin.69.fa'**	*Faecalibacterium prausnitzii* ATCC 27768	12.5	[9.8 - 15.7]	3.7	[2.8 - 4.8]	12.9	[10.6 - 15.6]	10.59
**'bin.69.fa'**	*Halopseudomonas xinjiangensis* NRRL B-51270	12.5	[9.8 - 15.7]	3.7	[2.8 - 4.8]	12.9	[10.6 - 15.6]	14.82
**'bin.69.fa'**	*Formosa agariphila* KMM 3901	12.5	[9.8 - 15.7]	3.7	[2.8 - 4.8]	12.9	[10.6 - 15.6]	12.4
**'bin.69.fa'**	*Flavobacterium hibisci* THG-HG1.4	12.5	[9.8 - 15.7]	3.7	[2.8 - 4.8]	12.9	[10.6 - 15.6]	11.48
**'bin.69.fa'**	*Caldanaerobacter subterraneus* subsp. *yonseiensis* DSM 13777	12.5	[9.8 - 15.7]	3.7	[2.8 - 4.8]	12.9	[10.6 - 15.6]	8.32
**'bin.69.fa'**	*Staphylococcus stepanovicii* DSM 26319	12.5	[9.8 - 15.7]	3.7	[2.8 - 4.8]	12.9	[10.6 - 15.6]	13.84
**'bin.69.fa'**	*Clostridium culturomicium* CL-6	12.5	[9.8 - 15.7]	3.7	[2.8 - 4.8]	12.9	[10.6 - 15.6]	13.65
**'bin.69.fa'**	*Cedecea neteri* ATCC 33855	12.5	[9.8 - 15.7]	3.7	[2.8 - 4.8]	12.9	[10.6 - 15.6]	8.25
**'bin.69.fa'**	*Limibacter armeniacum* KCTC 22132	12.5	[9.8 - 15.7]	3.7	[2.8 - 4.8]	12.9	[10.6 - 15.6]	5.4

Table 4: Strains in your dataset

Joint dataset of automatically determined closest type strains (if this mode was chosen), manually selected type strains (if selected accordingly) and the provided user strains, if provided (marked in yellow).

Strain	Authority	Other deposits	Synonyms	Base pairs	Percent G+C	No. proteins	Goldstamp	Bioproject accession	Biosample accession	Assembly accession	IMG OID
Staphylococcus stepanovicii DSM 26319	Hauschild et al. 2012	196; CCUG 72271; DSM 26319; NCTC 13839; CCM 7717; PCM 2693	*Mammaliicoccus stepanovicii*; *Staphylococcus stepanovicii*	2405 523	32.0	2373	Gp0357017	PRJNA224116	SAMN05978003	GCF_002902525	
Faecalibacterium prausnitzii ATCC 27768	(Hauduroy et al. 1937) Duncan et al. 2002 emend. Zou et al. 2021	NCIMB 13872	*Bacteroides praussnitzii*; *Faecalibacterium moorei*; *Faecalibacterium prausnitzii*; *Fusobacterium prausnitzii*	3024 736	56.5	2798	Gp0442026	PRJNA432550	SAMN08707943	GCA_003324185	
Clostridium culturomicium CL-6	Kieu et al. 2021	DSM 100507; CSUR P1184	*Clostridium culturomicium*	4323 983	32.2	4122		PRJEB6474	SAMEA2602735	GCA_000751675	
Flavobacterium hibisci THG-HG1.4	Moya et al. 2017	CCTCC AB 2016178; KACC 18852	*Flavobacterium hibisci*	4934 729	34.4	4069		PRJNA759806	SAMN21197698	GCA_020026925	
Limibacter armeniacum KCTC 22132	Yoon et al. 2008	DSM 26026; MBIC 8286; YM 11-185	*Limibacter armeniacum*	8038 033	40.5	5966		PRJNA224116	SAMN39988393	GCF_036880985	
Autumnicola patrickiae F188	Rey-Velasco et al. 2024	CECT 30805; CCM 9341	*Autumnicola patrickiae*	4331 166	38.4	3866		PRJNA224116	SAMN37377045	GCF_031846525	
Formosa agariphila KMM 3901	Nedashkovskaya et al. 2006 emend. Hahnke et al. 2016	LMG 23005; KCTC 12365; DSM 15362; DSM 19837	*Formosa agariphila*	4204 854	33.5	3570	Gp0100822	PRJEB3965	SAMEA3139019	GCA_000723205	

Strain	Authority	Other deposits	Synonyms	Base pairs	Percent G+C	No. proteins	Goldstamp	Bioproject accession	Biosample accession	Assembly accession	IMG OID
Caldanaerobacter subterraneus subsp. *yonseiensis* DSM 13777	(Kim et al. 2001) Fardeau et al. 2004	KB-1; KFCC 11116	*Caldanaerobacter subterraneus* subsp. *yonseiensis*; *Thermoanaerobacter yonseiensis*	2700541	37.5	2711	Gp0026979	PRJNA215704	SAMN02470224	GCA_000473865	2563367176
Cedecea neteri ATCC 33855	Farmer et al. 1983	002 of Grimont et al.; LMG 7864; CIP 103241; CCUG 18763; DSM 13693; JCM 7582; NCTC 12120; CDC 621-75; GTC 1717	*Cedecea neteri*	5203266	54.1	4843	Gp0023567	PRJDB240	SAMD00046737	GCA_001571265	
Halopseudomonas xinjiangensis NRRL B-51270	(Liu et al. 2009) Rudra and Gupta 2021	DSM 23391; CCTCC AB 207151; S3-3	*Halopseudomonas xinjiangensis*; *Neopseudomonas xinjiangensis*; *Pseudomonas xinjiangensis*	3536992	60.7	3246	Gp0127169	PRJEB16376	SAMN05216421	GCA_900104945	
bin.69.fa				2410580	45.9	2203					

Methods, Results and References

The genome sequence data were uploaded to the Type (Strain) Genome Server (TYGS), a free bioinformatics platform available under https://tygs.dsmz.de, for a whole genome-based taxonomic analysis [1]. The analysis also made use of recently introduced methodological updates and features [2]. Information on nomenclature, synonymy and associated taxonomic literature was provided by TYGS's sister database, the List of Prokaryotic names with Standing in Nomenclature (LPSN, available at https://lpsn.dsmz.de) [2]. The results were provided by the TYGS on 2024-06-16. The TYGS analysis was subdivided into the following steps:

Determination of closely related type strains

The determination of closely related type strains did not succeed because not a single 16S rDNA gene sequence was detected in the provided user genomes. The subsequent analyses are thus only based on the provided genome data and the manually selected type strains, if any.

Pairwise comparison of genome sequences

For the phylogenomic inference, all pairwise comparisons among the set of genomes were conducted using GBDP and accurate intergenomic distances inferred under the algorithm 'trimming' and distance formula d_5 [3]. 100 distance replicates were calculated each. Digital DDH values and confidence intervals were calculated using the recommended settings of the GGDC 4.0 [2,3].

Phylogenetic inference

The resulting intergenomic distances were used to infer a balanced minimum evolution tree with branch support via FASTME 2.1.6.1 including SPR postprocessing [4]. Branch support was inferred from 100 pseudo-bootstrap replicates each. The trees were rooted at the midpoint [5] and visualized with PhyD3 [6].

Type-based species and subspecies clustering

The type-based species clustering using a 70% dDDH radius around each of the 10 type strains was done as previously described [1]. The resulting groups are shown in Table 1 and 4. Subspecies clustering was done using a 79% dDDH threshold as previously introduced [7].

Results

Type-based species and subspecies clustering

The resulting species and subspecies clusters are listed in Table 4, whereas the taxonomic identification of the query strains is found in Table 1. Briefly, the clustering yielded 11 species clusters and the provided query strains were assigned to 1 of these. Moreover, user strains were located in 1 of 11 subspecies clusters.

Figure caption genome tree

Figure 2. Tree inferred with FastME 2.1.6.1 [4] from GBDP distances calculated from genome sequences. The branch lengths are scaled in terms of GBDP distance formula d_5. The numbers above branches are GBDP pseudo-bootstrap support values > 60 % from 100 replications, with an average branch support of 62.9 %. The tree was rooted at the midpoint [5].

References

[1] Meier-Kolthoff JP, Göker M. TYGS is an automated high-throughput platform for state-of-the-art genome-based taxonomy. Nat. Commun. 2019;10: 2182. DOI: 10.1038/s41467-019-10210-3

[2] Meier-Kolthoff JP, Sardà Carbasse J, Peinado-Olarte RL, Göker M. TYGS and LPSN: a database tandem for fast and reliable genome-based classification and nomenclature of prokaryotes. Nucleic Acid Res. 2022;50: D801–D807. DOI: 10.1093/nar/gkab902

[3] Meier-Kolthoff JP, Auch AF, Klenk H-P, Göker M. Genome sequence-based species delimitation with confidence intervals and improved distance functions. BMC Bioinformatics. 2013;14: 60. DOI: 10.1186/1471-2105-14-60

[4] Lefort V, Desper R, Gascuel O. FastME 2.0: A comprehensive, accurate, and fast distance-based phylogeny inference program. Mol Biol Evol. 2015;32: 2798–2800. DOI: 10.1093/molbev/msv150

[5] Farris JS. Estimating phylogenetic trees from distance matrices. Am Nat. 1972;106: 645–667.

[6] Kreft L, Botzki A, Coppens F, Vandepoele K, Van Bel M. PhyD3: A phylogenetic tree viewer with extended phyloXML support for functional genomics data visualization. Bioinformatics. 2017;33: 2946–2947. DOI: 10.1093/bioinformatics/btx324

[7] Meier-Kolthoff JP, Hahnke RL, Petersen J, Scheuner C, Michael V, Fiebig A, et al. Complete genome sequence of DSM 30083^T, the type strain (U5/41^T) of *Escherichia coli*, and a proposal for delineating subspecies in microbial taxonomy. Stand Genomic Sci. 2014;9: 2. DOI: 10.1186/1944-3277-9-2
