# Supplementary material for: Tracing active members in microbial communities by BONCAT and click chemistry-based enrichment of newly synthesized proteins
Source: ISME Commun. 2024 Dec 4;4(1):ycae153. doi: 10.1093/ismeco/ycae153 (PMC11683836; doi:10.1093/ismeco/ycae153)
Supplement: Genome_Server_ycae153 [file genome_server_ycae153.zip › Genome Server/Bin_70_TYGS_job_results.pdf]

TYPE (STRAIN) GENOME SERVER

JOB SUMMARY

PRINT DATE:	2024-06-17 08:40:23 +0200
JOB ID:	0a9b15f4-4f5a-4257-960e-91c2f7ea3a67
RESULT PAGE:	https://tygs.dsmz.de/user_results/show?guid=0a9b15f4-4f5a-4257-960e-91c2f7ea3a67

Table 1: Phylogenies

Publication-ready versions of both the genome-scale GBDP tree and the 16S rRNA gene sequence tree can be customized and exported either in SVG (vector graphic) or PNG format from within the phylogeny viewers in your TYGS result page. For publications the **SVG format is recommended** because it is lossless, always keeps its high resolution and can also be easily converted to other popular formats such as PDF or EPS. Please follow the link provided above!

Table 2: Identification

The below list contains the result of the TYGS species identification routine.

Explanation of remarks that might occur in the below table:

remark [R1]: The TYGS type strain database is automatically updated on an almost daily basis. However, if a particular type strain genome is not available in the TYGS database, this can have several reasons which are detailed in the FAQ. You can request an extended 16S rRNA gene analysis via the 16S tree viewer found in your result page to detect **not yet genome-sequenced** type strains relevant for your study.

remark [R2]: > 70% dDDH value (formula d_4) and (almost) minimal dDDH values for gene-content formulae d_0 and d_6 indicate a potentially unreliable identification result and should thus be checked via the 16S rRNA gene sequence similarity. Such strong deviations can, in principle, be caused by sequence contamination.

remark [R3]: G+C content difference of > 1 % indicates a potentially unreliable identification result because within species G+C content varies no more than 1 %, if computed from genome sequences (PMID: 24505073).

Strain	Conclusion	Identification result	Remark
**'bin.70'**	potential new species		see [R1]

Table 3: Pairwise comparisons of user genomes vs. type-strain genomes

The following table contains the pairwise dDDH values between your user genomes and the selected type-strain genomes. The dDDH values are provided along with their confidence intervals (C.I.) for the three different GBDP formulas:

- formula d_0 (a.k.a. GGDC formula 1): length of all HSPs divided by total genome length
- formula d_4 (a.k.a. GGDC formula 2): sum of all identities found in HSPs divided by overall HSP length
- formula d_6 (a.k.a. GGDC formula 3): sum of all identities found in HSPs divided by total genome length

Note: Formula d_4 is independent of genome length and is thus robust against the use of incomplete draft genomes. For other reasons for preferring formula d_4, see the FAQ.

Query	Subject	d_0	C.I. d_0	d_4	C.I. d_4	d_6	C.I. d_6	Diff. G+C Percent
**'bin.70.fa'**	*Tepidiphilus baoligensis* B18-69	40.0	[36.6 - 43.4]	53.3	[50.6 - 56.0]	41.3	[38.3 - 44.3]	0.89
**'bin.70.fa'**	*Tepidiphilus olei* J18	35.7	[32.3 - 39.2]	51.9	[49.2 - 54.6]	36.9	[34.0 - 40.0]	1.14
**'bin.70.fa'**	*Tepidiphilus succinatimandens* DSM 15512	34.4	[31.0 - 37.9]	41.6	[39.1 - 44.1]	34.4	[31.5 - 37.5]	0.38
**'bin.70.fa'**	*Tepidiphilus thermophilus* JCM 19170	35.8	[32.5 - 39.4]	41.4	[38.9 - 44.0]	35.7	[32.8 - 38.8]	0.67
**'bin.70.fa'**	*Tepidiphilus margaritifer* DSM 15129	35.4	[32.1 - 39.0]	38.9	[36.4 - 41.4]	35.0	[32.0 - 38.0]	0.67
**'bin.70.fa'**	*Tepidimonas fonticaldi* AT-A2	13.0	[10.3 - 16.3]	20.6	[18.4 - 23.0]	13.4	[11.0 - 16.1]	3.47
**'bin.70.fa'**	*Thauera aromatica* DSM 6984	13.2	[10.5 - 16.5]	18.8	[16.6 - 21.1]	13.5	[11.2 - 16.3]	1.64
**'bin.70.fa'**	*Azospira restricta* DSM18626T	13.2	[10.4 - 16.5]	17.2	[15.1 - 19.5]	13.5	[11.1 - 16.2]	3.11
**'bin.70.fa'**	*Ralstonia pickettii* NBRC 102503	12.6	[9.9 - 15.9]	17.2	[15.1 - 19.5]	13.0	[10.7 - 15.7]	1.56
**'bin.70.fa'**	*Stenotrophomonas daejeonensis* JCM 16244	12.6	[9.9 - 15.9]	16.5	[14.5 - 18.8]	13.0	[10.7 - 15.8]	3.04

Table 4: Strains in your dataset

Joint dataset of automatically determined closest type strains (if this mode was chosen), manually selected type strains (if selected accordingly) and the provided user strains, if provided (marked in yellow).

Strain	Authority	Other deposits	Synonyms	Base pairs	Percent G+C	No. proteins	Goldstamp	Bioproject accession	Biosample accession	Assembly accession	IMG OID
Azospira restricta DSM18626T	Bae et al. 2007	LMG 23819; NRRL B-41660; SUA2	*Azospira restricta*	3975213	68.6	3718		PRJNA676164	SAMN16756644	GCA_016858125	
Tepidiphilus olei J18	Wang et al. 2020	LMG 31400; CGMCC 1.16800	*Tepidiphilus olei*	2313533	64.4	2179		PRJNA513900	SAMN10716029	GCA_006980705	
Tepidiphilus baoligensis B18-69	Zhang et al. 2020	CGMCC 1.13573; KCTC 62782	*Tepidiphilus baoligensis*	2241689	64.6	2068		PRJNA601298	SAMN13865277	GCA_012911495	
Tepidiphilus margaritifer DSM 15129	Manaia et al. 2003 emend. Poddar et al. 2014	LMG 21637; N2-214	*Tepidiphilus margaritifer*	2218222	64.9	2133	Gp0012380	PRJNA188900	SAMN02440862	GCA_000425565	2524023151
Tepidimonas fonticaldi AT-A2	Chen et al. 2013	LMG 26746; BCRC 80391; KCTC 23862	*Tepidimonas fonticaldi*	3003776	69.0	2758		PRJNA552820	SAMN12219528	GCA_007556755	
Tepidiphilus succinatimandens DSM 15512	(Bonilla Salinas et al. 2004) Poddar et al. 2014	4BON; CIP 107790	*Petrobacter succinatimandens; Tepidiphilus succinatimandens*	2363988	65.9	2289		PRJNA224116	SAMN10716071	GCF_006503695	
Thauera aromatica DSM 6984	Anders et al. 1995	K 172	*Thauera aromatica*	3711792	67.2	3399	Gp0071507	PRJNA222478	SAMN02370758	GCA_003030465	
Tepidiphilus thermophilus JCM 19170	Poddar et al. 2014	LMG 27587; DSM 27220; JHK30	*Tepidiphilus thermophilus*	2258324	66.2	2186	Gp0108249	PRJEB10558	SAMN03840742	GCA_001517365	

Strain	Authority	Other deposits	Synonyms	Base pairs	Percent G+C	No. proteins	Goldstamp	Bioproject accession	Biosample accession	Assembly accession	IMG OID
Stenotrophomonas daejeonensis JCM 16244	Lee et al. 2011	KCTC 22451; DSM 26149; MJ03	*Stenotrophomonas daejeonensis*	3284859	68.6	2806	Gp0145543	PRJNA284378	SAMN03701730	GCA_001431505	
Ralstonia pickettii NBRC 102503	(Ralston et al. 1973) Yabuuchi et al. 1996	CFBP 2459; LMG 5942; CIP 73.23; ATCC 27511; CCUG 3318; DSM 6297; JCM 5969; NCTC 11149; HAMBI 2158	*Burkholderia pickettii*; *Pseudomonas pickettii*; *Ralstonia pickettii*	4733058	64.0	4352	Gp0024237	PRJDB287	SAMD00045738	GCA_001544155	
bin.70.fa				1120571	65.5	1262					

Methods, Results and References

The genome sequence data were uploaded to the Type (Strain) Genome Server (TYGS), a free bioinformatics platform available under https://tygs.dsmz.de, for a whole genome-based taxonomic analysis [1]. The analysis also made use of recently introduced methodological updates and features [2]. Information on nomenclature, synonymy and associated taxonomic literature was provided by TYGS's sister database, the List of Prokaryotic names with Standing in Nomenclature (LPSN, available at https://lpsn.dsmz.de) [2]. The results were provided by the TYGS on 2024-06-16. The TYGS analysis was subdivided into the following steps:

Determination of closely related type strains

The determination of closely related type strains did not succeed because not a single 16S rDNA gene sequence was detected in the provided user genomes. The subsequent analyses are thus only based on the provided genome data and the manually selected type strains, if any.

Pairwise comparison of genome sequences

For the phylogenomic inference, all pairwise comparisons among the set of genomes were conducted using GBDP and accurate intergenomic distances inferred under the algorithm 'trimming' and distance formula d_5 [3]. 100 distance replicates were calculated each. Digital DDH values and confidence intervals were calculated using the recommended settings of the GGDC 4.0 [2,3].

Phylogenetic inference

The resulting intergenomic distances were used to infer a balanced minimum evolution tree with branch support via FASTME 2.1.6.1 including SPR postprocessing [4]. Branch support was inferred from 100 pseudo-bootstrap replicates each. The trees were rooted at the midpoint [5] and visualized with PhyD3 [6].

Type-based species and subspecies clustering

The type-based species clustering using a 70% dDDH radius around each of the 10 type strains was done as previously described [1]. The resulting groups are shown in Table 1 and 4. Subspecies clustering was done using a 79% dDDH threshold as previously introduced [7].

Results

Type-based species and subspecies clustering

The resulting species and subspecies clusters are listed in Table 4, whereas the taxonomic identification of the query strains is found in Table 1. Briefly, the clustering yielded 9 species clusters and the provided query strains were assigned to 1 of these. Moreover, user strains were located in 1 of 10 subspecies clusters.

Figure caption genome tree

Figure 2. Tree inferred with FastME 2.1.6.1 [4] from GBDP distances calculated from genome sequences. The branch lengths are scaled in terms of GBDP distance formula d_5. The numbers above branches are GBDP pseudo-bootstrap support values > 60 % from 100 replications, with an average branch support of 84.6 %. The tree was rooted at the midpoint [5].

References

[1] Meier-Kolthoff JP, Göker M. TYGS is an automated high-throughput platform for state-of-the-art genome-based taxonomy. Nat. Commun. 2019;10: 2182. DOI: 10.1038/s41467-019-10210-3

[2] Meier-Kolthoff JP, Sardà Carbasse J, Peinado-Olarte RL, Göker M. TYGS and LPSN: a database tandem for fast and reliable genome-based classification and nomenclature of prokaryotes. Nucleic Acid Res. 2022;50: D801–D807. DOI: 10.1093/nar/gkab902

[3] Meier-Kolthoff JP, Auch AF, Klenk H-P, Göker M. Genome sequence-based species delimitation with confidence intervals and improved distance functions. BMC Bioinformatics. 2013;14: 60. DOI: 10.1186/1471-2105-14-60

[4] Lefort V, Desper R, Gascuel O. FastME 2.0: A comprehensive, accurate, and fast distance-based phylogeny inference program. Mol Biol Evol. 2015;32: 2798–2800. DOI: 10.1093/molbev/msv150

[5] Farris JS. Estimating phylogenetic trees from distance matrices. Am Nat. 1972;106: 645–667.

[6] Kreft L, Botzki A, Coppens F, Vandepoele K, Van Bel M. PhyD3: A phylogenetic tree viewer with extended phyloXML support for functional genomics data visualization. Bioinformatics. 2017;33: 2946–2947. DOI: 10.1093/bioinformatics/btx324

[7] Meier-Kolthoff JP, Hahnke RL, Petersen J, Scheuner C, Michael V, Fiebig A, et al. Complete genome sequence of DSM 30083^{T}, the type strain (U5/41^{T}) of *Escherichia coli*, and a proposal for delineating subspecies in microbial taxonomy. Stand Genomic Sci. 2014;9: 2. DOI: 10.1186/1944-3277-9-2
