# Supplementary material for: Tracing active members in microbial communities by BONCAT and click chemistry-based enrichment of newly synthesized proteins
Source: ISME Commun. 2024 Dec 4;4(1):ycae153. doi: 10.1093/ismeco/ycae153 (PMC11683836; doi:10.1093/ismeco/ycae153)
Supplement: Genome_Server_ycae153 [file genome_server_ycae153.zip › Genome Server/Bin_74_TYGS_job_results.pdf]

TYPE (STRAIN) GENOME SERVER

JOB SUMMARY

PRINT DATE:	2024-06-17 09:26:44 +0200
JOB ID:	8ce6b427-b617-4191-b16f-6815bae704ea--25
RESULT PAGE:	https://tygs.dsmz.de/user_results/show?guid=8ce6b427-b617-4191-b16f-6815bae704ea--25

Table 1: Phylogenies

Publication-ready versions of both the genome-scale GBDP tree and the 16S rRNA gene sequence tree can be customized and exported either in SVG (vector graphic) or PNG format from within the phylogeny viewers in your TYGS result page. For publications the **SVG format is recommended** because it is lossless, always keeps its high resolution and can also be easily converted to other popular formats such as PDF or EPS. Please follow the link provided above!

Table 2: Identification

The below list contains the result of the TYGS species identification routine.

Explanation of remarks that might occur in the below table:

remark [R1]: The TYGS type strain database is automatically updated on an almost daily basis. However, if a particular type strain genome is not available in the TYGS database, this can have several reasons which are detailed in the FAQ. You can request an extended 16S rRNA gene analysis via the 16S tree viewer found in your result page to detect **not yet genome-sequenced** type strains relevant for your study.

remark [R2]: > 70% dDDH value (formula d_4) and (almost) minimal dDDH values for gene-content formulae d_0 and d_6 indicate a potentially unreliable identification result and should thus be checked via the 16S rRNA gene sequence similarity. Such strong deviations can, in principle, be caused by sequence contamination.

remark [R3]: G+C content difference of > 1 % indicates a potentially unreliable identification result because within species G+C content varies no more than 1 %, if computed from genome sequences (PMID: 24505073).

Strain	Conclusion	Identification result	Remark
**'bin.74'**	potential new species		see [R1]

Table 3: Pairwise comparisons of user genomes vs. type-strain genomes

The following table contains the pairwise dDDH values between your user genomes and the selected type-strain genomes. The dDDH values are provided along with their confidence intervals (C.I.) for the three different GBDP formulas:

- formula d_0 (a.k.a. GGDC formula 1): length of all HSPs divided by total genome length
- formula d_4 (a.k.a. GGDC formula 2): sum of all identities found in HSPs divided by overall HSP length
- formula d_6 (a.k.a. GGDC formula 3): sum of all identities found in HSPs divided by total genome length

Note: Formula d_4 is independent of genome length and is thus robust against the use of incomplete draft genomes. For other reasons for preferring formula d_4, see the FAQ.

Query	Subject	d_0	C.I. d_0	d_4	C.I. d_4	d_6	C.I. d_6	Diff. G+C Percent
**'bin.74.fa'**	*Desulfotignum phosphitoxidans* DSM 13687	12.5	[9.8 - 15.7]	41.9	[39.4 - 44.4]	12.9	[10.6 - 15.6]	0.02
**'bin.74.fa'**	*Desulfotignum balticum* DSM 7044	12.5	[9.8 - 15.7]	41.3	[38.8 - 43.9]	12.9	[10.6 - 15.6]	0.04
**'bin.74.fa'**	*Candidatus* Syntrophus pelomyxae SKADARSKE-3	12.5	[9.9 - 15.8]	18.4	[16.3 - 20.8]	12.9	[10.6 - 15.7]	1.33
**'bin.74.fa'**	*Roseomonas algicola* PeD5	12.5	[9.8 - 15.7]	15.2	[13.2 - 17.5]	12.9	[10.6 - 15.6]	19.75
**'bin.74.fa'**	*Dokdonella immobilis* CGMCC 1.7659	12.5	[9.8 - 15.7]	3.7	[2.8 - 4.8]	12.9	[10.6 - 15.6]	13.71
**'bin.74.fa'**	*Taibaiella koreensis* THG-DT86	12.5	[9.8 - 15.7]	3.7	[2.8 - 4.8]	12.9	[10.6 - 15.6]	1.36
**'bin.74.fa'**	*Methylovirgula ligni* DSM 19998	12.5	[9.8 - 15.7]	3.7	[2.8 - 4.8]	12.9	[10.6 - 15.6]	11.02
**'bin.74.fa'**	*Rhodocista centenaria* ATCC 51521	12.5	[9.8 - 15.7]	3.7	[2.8 - 4.8]	12.9	[10.6 - 15.6]	19.19
**'bin.74.fa'**	*Candidatus* Merdivicinus excrementipullorum CHK199-13235	12.5	[9.8 - 15.7]	3.7	[2.8 - 4.8]	12.9	[10.6 - 15.6]	3.22
**'bin.74.fa'**	*Dyella sedimenti* N-S-14	12.5	[9.8 - 15.7]	3.7	[2.8 - 4.8]	12.9	[10.6 - 15.6]	16.27

Table 4: Strains in your dataset

Joint dataset of automatically determined closest type strains (if this mode was chosen), manually selected type strains (if selected accordingly) and the provided user strains, if provided (marked in yellow).

Strain	Authority	Other deposits	Synonyms	Base pairs	Percent G+C	No. proteins	Goldstamp	Bioproject accession	Biosample accession	Assembly accession	IMG OID
Roseomonas algicola PeD5	Kim et al. 2020	JCM 33309; KACC 19925	*Falsiroseomonas algicola*; *Roseomonas algicola*	6556 345	71.0	6082		PRJNA606129	SAMN14085714	GCA_011040385	
Dokdonella immobilis CGMCC 1.7659	Liu et al. 2013	JCM 15763; LM 2-5	*Dokdonella immobilis*	4634 500	65.0	3902	Gp0119964	PRJEB17133	SAMN05216289	GCA_900115085	
Candidatus Merdivicinus excrementipullorum CHK199-13235	Gilroy et al. 2021		*Candidatus* Merdivicinus excrementipullorum	2778 595	54.5	2561		PRJNA543206	SAMN15817032	GCA_018716725	
Rhodocista centenaria ATCC 51521	Kawasaki et al. 1994	ATCC 43720; DSM 8284; DSM 9894; JCM 21060; IFO 16667; NBRC 16667; IAM 14193	*Rhodocista centenaria*; *Rhodospirillum centenum*	4355 543	70.5	4003	Gp0001825	PRJNA18307	SAMN02603908	GCA_000016185	643348571
Methylovirgula ligni DSM 19998	Vorob'ev et al. 2009	NCIMB 14408; BW863	*Methylovirgula ligni*	3292 527	62.3	3205	Gp0290495	PRJNA456020	SAMN09064159	GCA_003387225	2770939601
Desulfotignum phosphitoxidans DSM 13687	Schink et al. 2002	FiPS-3; OCM 818	*Desulfotignum phosphitoxidans*	4998 761	51.3	4646	Gp0013876	PRJNA187267	SAMN02469830	GCA_000350545	2523231053
Desulfotignum balticum DSM 7044	Kuever et al. 2001	ATCC BAA-19; Sax	*Desulfotignum balticum*	5118 755	51.2	4693	Gp0015885	PRJNA195832	SAMN02440581	GCA_000421285	2526164528
Candidatus Syntrophus pelomyxae SKADARSKE-3	Treitli et al. 2023		*Candidatus* Syntrophus pelomyxae	4972 172	49.9	4474		PRJNA672820	SAMN33331050	GCA_030920355	
Taibaiella koreensis THG-DT86	Son et al. 2014	JCM 18823; KACC 17171	*Taibaiella koreensis*	5932 417	49.9	5005	Gp0374616	PRJNA224116	SAMN09829961	GCA_003545715	

Strain	Authority	Other deposits	Synonyms	Base pairs	Percent G+C	No. proteins	Goldstamp	Bioproject accession	Biosample accession	Assembly accession	IMG OID
Dyella sedimenti N-S-14	Gao et al. 2022	CGMCC 1.18717; KCTC 82384	*Dyella sedimenti*	4304 064	67.5	3755		PRJNA808774	SAMN26105026	GCA_022760995	
bin.74.fa				2652 272	51.3	2421					

Methods, Results and References

The genome sequence data were uploaded to the Type (Strain) Genome Server (TYGS), a free bioinformatics platform available under https://tygs.dsmz.de, for a whole genome-based taxonomic analysis [1]. The analysis also made use of recently introduced methodological updates and features [2]. Information on nomenclature, synonymy and associated taxonomic literature was provided by TYGS's sister database, the List of Prokaryotic names with Standing in Nomenclature (LPSN, available at https://lpsn.dsmz.de) [2]. The results were provided by the TYGS on 2024-06-16. The TYGS analysis was subdivided into the following steps:

Determination of closely related type strains

The determination of closely related type strains did not succeed because not a single 16S rDNA gene sequence was detected in the provided user genomes. The subsequent analyses are thus only based on the provided genome data and the manually selected type strains, if any.

Pairwise comparison of genome sequences

For the phylogenomic inference, all pairwise comparisons among the set of genomes were conducted using GBDP and accurate intergenomic distances inferred under the algorithm 'trimming' and distance formula d_5 [3]. 100 distance replicates were calculated each. Digital DDH values and confidence intervals were calculated using the recommended settings of the GGDC 4.0 [2,3].

Phylogenetic inference

The resulting intergenomic distances were used to infer a balanced minimum evolution tree with branch support via FASTME 2.1.6.1 including SPR postprocessing [4]. Branch support was inferred from 100 pseudo-bootstrap replicates each. The trees were rooted at the midpoint [5] and visualized with PhyD3 [6].

Type-based species and subspecies clustering

The type-based species clustering using a 70% dDDH radius around each of the 10 type strains was done as previously described [1]. The resulting groups are shown in Table 1 and 4. Subspecies clustering was done using a 79% dDDH threshold as previously introduced [7].

Results

Type-based species and subspecies clustering

The resulting species and subspecies clusters are listed in Table 4, whereas the taxonomic identification of the query strains is found in Table 1. Briefly, the clustering yielded 10 species clusters and the provided query strains were assigned to 1 of these. Moreover, user strains were located in 1 of 11 subspecies clusters.

Figure caption genome tree

Figure 2. Tree inferred with FastME 2.1.6.1 [4] from GBDP distances calculated from genome sequences. The branch lengths are scaled in terms of GBDP distance formula d_5. The numbers above branches are GBDP pseudo-bootstrap support values > 60 % from 100 replications, with an average branch support of 79.9 %. The tree was rooted at the midpoint [5].

References

[1] Meier-Kolthoff JP, Göker M. TYGS is an automated high-throughput platform for state-of-the-art genome-based taxonomy. Nat. Commun. 2019;10: 2182. DOI: 10.1038/s41467-019-10210-3

[2] Meier-Kolthoff JP, Sardà Carbasse J, Peinado-Olarte RL, Göker M. TYGS and LPSN: a database tandem for fast and reliable genome-based classification and nomenclature of prokaryotes. Nucleic Acid Res. 2022;50: D801–D807. DOI: 10.1093/nar/gkab902

[3] Meier-Kolthoff JP, Auch AF, Klenk H-P, Göker M. Genome sequence-based species delimitation with confidence intervals and improved distance functions. BMC Bioinformatics. 2013;14: 60. DOI: 10.1186/1471-2105-14-60

[4] Lefort V, Desper R, Gascuel O. FastME 2.0: A comprehensive, accurate, and fast distance-based phylogeny inference program. Mol Biol Evol. 2015;32: 2798–2800. DOI: 10.1093/molbev/msv150

[5] Farris JS. Estimating phylogenetic trees from distance matrices. Am Nat. 1972;106: 645–667.

[6] Kreft L, Botzki A, Coppens F, Vandepoele K, Van Bel M. PhyD3: A phylogenetic tree viewer with extended phyloXML support for functional genomics data visualization. Bioinformatics. 2017;33: 2946–2947. DOI: 10.1093/bioinformatics/btx324

[7] Meier-Kolthoff JP, Hahnke RL, Petersen J, Scheuner C, Michael V, Fiebig A, et al. Complete genome sequence of DSM 30083^{T}, the type strain (U5/41^{T}) of *Escherichia coli*, and a proposal for delineating subspecies in microbial taxonomy. Stand Genomic Sci. 2014;9: 2. DOI: 10.1186/1944-3277-9-2
