# Supplementary material for: Tracing active members in microbial communities by BONCAT and click chemistry-based enrichment of newly synthesized proteins
Source: ISME Commun. 2024 Dec 4;4(1):ycae153. doi: 10.1093/ismeco/ycae153 (PMC11683836; doi:10.1093/ismeco/ycae153)
Supplement: Genome_Server_ycae153 [file genome_server_ycae153.zip › Genome Server/Bin_75_TYGS_job_results.pdf]

TYPE (STRAIN) GENOME SERVER

JOB SUMMARY

PRINT DATE:	2024-06-17 09:30:23 +0200
JOB ID:	8ce6b427-b617-4191-b16f-6815bae704ea--26
RESULT PAGE:	https://tygs.dsmz.de/user_results/show?guid=8ce6b427-b617-4191-b16f-6815bae704ea--26

Table 1: Phylogenies

Publication-ready versions of both the genome-scale GBDP tree and the 16S rRNA gene sequence tree can be customized and exported either in SVG (vector graphic) or PNG format from within the phylogeny viewers in your TYGS result page. For publications the **SVG format is recommended** because it is lossless, always keeps its high resolution and can also be easily converted to other popular formats such as PDF or EPS. Please follow the link provided above!

Table 2: Identification

The below list contains the result of the TYGS species identification routine.

Explanation of remarks that might occur in the below table:

remark [R1]: The TYGS type strain database is automatically updated on an almost daily basis. However, if a particular type strain genome is not available in the TYGS database, this can have several reasons which are detailed in the FAQ. You can request an extended 16S rRNA gene analysis via the 16S tree viewer found in your result page to detect **not yet genome-sequenced** type strains relevant for your study.

remark [R2]: > 70% dDDH value (formula d_4) and (almost) minimal dDDH values for gene-content formulae d_0 and d_6 indicate a potentially unreliable identification result and should thus be checked via the 16S rRNA gene sequence similarity. Such strong deviations can, in principle, be caused by sequence contamination.

remark [R3]: G+C content difference of > 1 % indicates a potentially unreliable identification result because within species G+C content varies no more than 1 %, if computed from genome sequences (PMID: 24505073).

Strain	Conclusion	Identification result	Remark
**'bin.75'**	potential new species		see [R1]

Table 3: Pairwise comparisons of user genomes vs. type-strain genomes

The following table contains the pairwise dDDH values between your user genomes and the selected type-strain genomes. The dDDH values are provided along with their confidence intervals (C.I.) for the three different GBDP formulas:

- formula d_0 (a.k.a. GGDC formula 1): length of all HSPs divided by total genome length
- formula d_4 (a.k.a. GGDC formula 2): sum of all identities found in HSPs divided by overall HSP length
- formula d_6 (a.k.a. GGDC formula 3): sum of all identities found in HSPs divided by total genome length

Note: Formula d_4 is independent of genome length and is thus robust against the use of incomplete draft genomes. For other reasons for preferring formula d_4, see the FAQ.

Query	Subject	d_0	C.I. d_0	d_4	C.I. d_4	d_6	C.I. d_6	Diff. G+C Percent
**'bin.75.fa'**	*Amycolatopsis nigrescens* CSC17Ta-90	12.5	[9.8 - 15.7]	29.2	[26.9 - 31.7]	12.9	[10.6 - 15.6]	15.69
**'bin.75.fa'**	*Streptomyces smaragdinus* RB5	12.5	[9.8 - 15.7]	28.2	[25.8 - 30.7]	12.9	[10.6 - 15.6]	17.7
**'bin.75.fa'**	*Streptomyces lasiicapitis* CGMCC 4.7349	12.5	[9.8 - 15.7]	28.2	[25.8 - 30.7]	12.9	[10.6 - 15.6]	17.17
**'bin.75.fa'**	*Azospirillum aestuarii* YIM DDC1	12.5	[9.8 - 15.7]	12.6	[10.8 - 14.7]	12.9	[10.6 - 15.6]	14.14
**'bin.75.fa'**	*Aquabacterium commune* DSM 11901	12.5	[9.8 - 15.7]	3.7	[2.8 - 4.8]	12.9	[10.6 - 15.6]	12.03
**'bin.75.fa'**	*Paracoccus versutus* DSM 582	12.5	[9.8 - 15.7]	3.7	[2.8 - 4.8]	12.9	[10.6 - 15.6]	13.24
**'bin.75.fa'**	*Ensifer aridi* LMR001	12.5	[9.8 - 15.7]	3.7	[2.8 - 4.8]	12.9	[10.6 - 15.6]	7.43
**'bin.75.fa'**	*Telluria mixta* LMG 11547	12.5	[9.8 - 15.7]	3.7	[2.8 - 4.8]	12.9	[10.6 - 15.6]	11.69
**'bin.75.fa'**	*Azospirillum palustre* B2	12.5	[9.8 - 15.7]	3.7	[2.8 - 4.8]	12.9	[10.6 - 15.6]	13.52
**'bin.75.fa'**	*Paracoccus bengalensis* DSM 17099	12.5	[9.8 - 15.7]	3.7	[2.8 - 4.8]	12.9	[10.6 - 15.6]	13.05

Table 4: Strains in your dataset

Joint dataset of automatically determined closest type strains (if this mode was chosen), manually selected type strains (if selected accordingly) and the provided user strains, if provided (marked in yellow).

Strain	Authority	Other deposits	Synonyms	Base pairs	Percent G+C	No. proteins	Goldstamp	Bioproject accession	Biosample accession	Assembly accession	IMG OID
Paracoccus bengalensis DSM 17099	Ghosh et al. 2006 emend. Hördt et al. 2020	LMG 22700; JJJ; MTCC 7003	*Paracoccus bengalensis*	4989 582	67.4	4932	Gp0131664	PRJNA332070	SAMN05443527	GCA_003386575	2773857629
Azospirillum aestuarii YIM DDC1	Xu et al. 2023	CGMCC 1.17325; KCTC 42887	*Azospirillum aestuarii*	7138 859	68.5	6366		PRJNA690667	SAMN17256624	GCA_016632405	
Azospirillum palustre B2	Tikhonova et al. 2019	KCTC 62613; VKM B-3233	*Azospirillum palustre*	7989 338	67.8	6821	Gp0253205	PRJNA414085	SAMN07776893	GCA_002573965	
Telluria mixta LMG 11547	(Bowman et al. 1989) Bowman et al. 1993	ACM 1762; CIP 104068; ATCC 49108; CCUG 35206; DSM 29330; DSM 4832; UQM 1762	*Pseudomonas mixta*; *Telluria mixta*	7388 759	66.0	6341		PRJNA868536	SAMN30252720	GCA_024756225	
Aquabacterium commune DSM 11901	Kalmbach et al. 1999	CIP 106984; ATCC BAA-209; CCUG 48318; B 8	*Aquabacterium commune*	4204 303	66.4	3653	Gp0325727	PRJNA520314	SAMN10866316	GCA_004362855	2795385467

Strain	Authority	Other deposits	Synonyms	Base pairs	Percent G+C	No. proteins	Goldstamp	Bioproject accession	Biosample accession	Assembly accession	IMG OID
Paracoccus versutus DSM 582	(Harrison 1983) Katayama et al. 1996 emend. Liu et al. 2020	A2; CIP 107117; NCCB 80062; NCCB 81078; ATCC 25364; JCM 20754; IFO 14567; NBRC 14567; VKM B-2163; IAM 12814; THI O41	*Paracoccus versutus*; *Thiobacillus rapidicrescens*; *Thiobacillus versutus*	5625 517	67.6	5451	Gp0112494	PRJNA303497	SAMN04488022	GCA_003387045	2615840723
Streptomyces lasiicapitis CGMCC 4.7349	Ye et al. 2017	3H-HV17(2); DSM 103124	*Streptomyces lasiicapitis*	9738 767	71.5	8382		PRJDB10509	SAMD00245204	GCA_014646335	
Amycolatopsis nigrescens CSC17Ta-90	Groth et al. 2007 emend. Nouioui et al. 2018	NRRL B-24473; DSM 44992; JCM 14717; HKI 330	*Amycolatopsis nigrescens*	9111 007	70.0	8550	Gp0012240	PRJNA82633	SAMN02261308	GCA_000384315	2515075013
Streptomyces smaragdinus RB5	Schwitalla et al. 2020	NRRL B-65539; VKM Ac-2839	*Streptomyces smaragdinus*	7852 112	72.0	7040		PRJNA545686	SAMN11898449	GCA_009604385	
Ensifer aridi LMR001	Rocha et al. 2020	LMG 31426; HAMBI 3707	*Ensifer aridi*	6585 387	61.7	6217		PRJNA224116	SAMN04455909	GCF_002078505	
bin.75.fa				1961 929	54.3	1565					

Methods, Results and References

The genome sequence data were uploaded to the Type (Strain) Genome Server (TYGS), a free bioinformatics platform available under https://tygs.dsmz.de, for a whole genome-based taxonomic analysis [1]. The analysis also made use of recently introduced methodological updates and features [2]. Information on nomenclature, synonymy and associated taxonomic literature was provided by TYGS's sister database, the List of Prokaryotic names with Standing in Nomenclature (LPSN, available at https://lpsn.dsmz.de) [2]. The results were provided by the TYGS on 2024-06-16. The TYGS analysis was subdivided into the following steps:

Determination of closely related type strains

The determination of closely related type strains did not succeed because not a single 16S rDNA gene sequence was detected in the provided user genomes. The subsequent analyses are thus only based on the provided genome data and the manually selected type strains, if any.

Pairwise comparison of genome sequences

For the phylogenomic inference, all pairwise comparisons among the set of genomes were conducted using GBDP and accurate intergenomic distances inferred under the algorithm 'trimming' and distance formula d_5 [3]. 100 distance replicates were calculated each. Digital DDH values and confidence intervals were calculated using the recommended settings of the GGDC 4.0 [2,3].

Phylogenetic inference

The resulting intergenomic distances were used to infer a balanced minimum evolution tree with branch support via FASTME 2.1.6.1 including SPR postprocessing [4]. Branch support was inferred from 100 pseudo-bootstrap replicates each. The trees were rooted at the midpoint [5] and visualized with PhyD3 [6].

Type-based species and subspecies clustering

The type-based species clustering using a 70% dDDH radius around each of the 10 type strains was done as previously described [1]. The resulting groups are shown in Table 1 and 4. Subspecies clustering was done using a 79% dDDH threshold as previously introduced [7].

Results

Type-based species and subspecies clustering

The resulting species and subspecies clusters are listed in Table 4, whereas the taxonomic identification of the query strains is found in Table 1. Briefly, the clustering yielded 10 species clusters and the provided query strains were assigned to 1 of these. Moreover, user strains were located in 1 of 10 subspecies clusters.

Figure caption genome tree

Figure 2. Tree inferred with FastME 2.1.6.1 [4] from GBDP distances calculated from genome sequences. The branch lengths are scaled in terms of GBDP distance formula d_5. The numbers above branches are GBDP pseudo-bootstrap support values > 60 % from 100 replications, with an average branch support of 89.1 %. The tree was rooted at the midpoint [5].

References

[1] Meier-Kolthoff JP, Göker M. TYGS is an automated high-throughput platform for state-of-the-art genome-based taxonomy. Nat. Commun. 2019;10: 2182. DOI: 10.1038/s41467-019-10210-3

[2] Meier-Kolthoff JP, Sardà Carbasse J, Peinado-Olarte RL, Göker M. TYGS and LPSN: a database tandem for fast and reliable genome-based classification and nomenclature of prokaryotes. Nucleic Acid Res. 2022;50: D801–D807. DOI: 10.1093/nar/gkab902

[3] Meier-Kolthoff JP, Auch AF, Klenk H-P, Göker M. Genome sequence-based species delimitation with confidence intervals and improved distance functions. BMC Bioinformatics. 2013;14: 60. DOI: 10.1186/1471-2105-14-60

[4] Lefort V, Desper R, Gascuel O. FastME 2.0: A comprehensive, accurate, and fast distance-based phylogeny inference program. Mol Biol Evol. 2015;32: 2798–2800. DOI: 10.1093/molbev/msv150

[5] Farris JS. Estimating phylogenetic trees from distance matrices. Am Nat. 1972;106: 645–667.

[6] Kreft L, Botzki A, Coppens F, Vandepoele K, Van Bel M. PhyD3: A phylogenetic tree viewer with extended phyloXML support for functional genomics data visualization. Bioinformatics. 2017;33: 2946–2947. DOI: 10.1093/bioinformatics/btx324

[7] Meier-Kolthoff JP, Hahnke RL, Petersen J, Scheuner C, Michael V, Fiebig A, et al. Complete genome sequence of DSM 30083^{T}, the type strain (U5/41^{T}) of *Escherichia coli*, and a proposal for delineating subspecies in microbial taxonomy. Stand Genomic Sci. 2014;9: 2. DOI: 10.1186/1944-3277-9-2
