# Supplementary material for: Tracing active members in microbial communities by BONCAT and click chemistry-based enrichment of newly synthesized proteins
Source: ISME Commun. 2024 Dec 4;4(1):ycae153. doi: 10.1093/ismeco/ycae153 (PMC11683836; doi:10.1093/ismeco/ycae153)
Supplement: Genome_Server_ycae153 [file genome_server_ycae153.zip › Genome Server/Bin_7_Bin_34_Bin_67_Bin_71_TYGS_job_results.pdf]

TYPE (STRAIN) GENOME SERVER

JOB SUMMARY

PRINT DATE:	2024-06-17 09:31:41 +0200
JOB ID:	8ce6b427-b617-4191-b16f-6815bae704ea--22
RESULT PAGE:	https://tygs.dsmz.de/user_results/show?guid=8ce6b427-b617-4191-b16f-6815bae704ea--22

Table 1: Phylogenies

Publication-ready versions of both the genome-scale GBDP tree and the 16S rRNA gene sequence tree can be customized and exported either in SVG (vector graphic) or PNG format from within the phylogeny viewers in your TYGS result page. For publications the **SVG format is recommended** because it is lossless, always keeps its high resolution and can also be easily converted to other popular formats such as PDF or EPS. Please follow the link provided above!

Table 2: Identification

The below list contains the result of the TYGS species identification routine.

Explanation of remarks that might occur in the below table:

remark [R1]: The TYGS type strain database is automatically updated on an almost daily basis. However, if a particular type strain genome is not available in the TYGS database, this can have several reasons which are detailed in the FAQ. You can request an extended 16S rRNA gene analysis via the 16S tree viewer found in your result page to detect **not yet genome-sequenced** type strains relevant for your study.

remark [R2]: > 70% dDDH value (formula d_4) and (almost) minimal dDDH values for gene-content formulae d_0 and d_6 indicate a potentially unreliable identification result and should thus be checked via the 16S rRNA gene sequence similarity. Such strong deviations can, in principle, be caused by sequence contamination.

remark [R3]: G+C content difference of > 1 % indicates a potentially unreliable identification result because within species G+C content varies no more than 1 %, if computed from genome sequences (PMID: 24505073).

Strain	Conclusion	Identification result	Remark
**'bin.67'**	belongs to known species	*Halopseudomonas salegens*	see [R2]<br>see [R3]
**'bin.7'**	potential new species		see [R1]
**'bin.34'**	potential new species		see [R1]
**'bin.71'**	potential new species		see [R1]

Table 3: Pairwise comparisons of user genomes vs. type-strain genomes

The following table contains the pairwise dDDH values between your user genomes and the selected type-strain genomes. The dDDH values are provided along with their confidence intervals (C.I.) for the three different GBDP formulas:

- formula d_0 (a.k.a. GGDC formula 1): length of all HSPs divided by total genome length
- formula d_4 (a.k.a. GGDC formula 2): sum of all identities found in HSPs divided by overall HSP length
- formula d_6 (a.k.a. GGDC formula 3): sum of all identities found in HSPs divided by total genome length

Note: Formula d_4 is independent of genome length and is thus robust against the use of incomplete draft genomes. For other reasons for preferring formula d_4, see the FAQ.

Query	Subject	d_0	C.I. d_0	d_4	C.I. d_4	d_6	C.I. d_6	Diff. G+C Percent
**'bin.67.fa'**	*Halopseudomonas salegens* CECT 8338	12.5	[9.8 - 15.7]	76.6	[73.6 - 79.3]	12.9	[10.6 - 15.6]	1.81
**'bin.7.fa'**	*Halopseudomonas salegens* CECT 8338	12.5	[9.8 - 15.7]	60.6	[57.7 - 63.4]	12.9	[10.6 - 15.6]	2.82
**'bin.7.fa'**	*Pseudomonas panipatensis* CCM 7469	12.5	[9.8 - 15.7]	54.3	[51.6 - 57.0]	12.9	[10.6 - 15.6]	10.69
**'bin.7.fa'**	*Methylogaea oryzae* JCM 16910	12.5	[9.8 - 15.7]	54.2	[51.5 - 56.9]	12.9	[10.6 - 15.6]	7.4
**'bin.67.fa'**	*Pseudomonas brassicae* MAFF 212427	12.5	[9.8 - 15.7]	53.7	[51.0 - 56.4]	12.9	[10.6 - 15.6]	3.91
**'bin.67.fa'**	*Pseudomonas panipatensis* CCM 7469	12.5	[9.8 - 15.7]	52.8	[50.1 - 55.5]	12.9	[10.6 - 15.6]	6.05
**'bin.7.fa'**	*Pseudomonas brassicae* MAFF 212427	12.5	[9.8 - 15.7]	52.7	[50.0 - 55.4]	12.9	[10.6 - 15.6]	8.55
**'bin.71.fa'**	*Mycolicibacterium aurantiacum* 3033	12.5	[9.8 - 15.7]	52.5	[49.8 - 55.2]	12.9	[10.6 - 15.6]	10.86
**'bin.67.fa'**	*Mycolicibacterium aurantiacum* 3033	12.5	[9.8 - 15.7]	52.5	[49.8 - 55.2]	12.9	[10.6 - 15.6]	8.6
**'bin.67.fa'**	*Trichlorobacter ammonificans* G1	12.5	[9.8 - 15.7]	50.7	[48.1 - 53.4]	12.9	[10.6 - 15.6]	1.8
**'bin.7.fa'**	*Trichlorobacter ammonificans* G1	12.5	[9.8 - 15.7]	40.0	[37.5 - 42.5]	12.9	[10.6 - 15.6]	6.43
**'bin.71.fa'**	*Candidatus* Alectryocaccomicrobium excrementavium 13766	12.5	[9.8 - 15.7]	39.5	[37.0 - 42.0]	12.9	[10.6 - 15.6]	2.66
**'bin.67.fa'**	*Candidatus* Alectryocaccomicrobium excrementavium 13766	12.5	[9.8 - 15.7]	37.1	[34.7 - 39.6]	12.9	[10.6 - 15.6]	0.4
**'bin.34.fa'**	*Pulveribacter suum* KACC 19309T	12.5	[9.8 - 15.7]	32.4	[30.0 - 34.9]	12.9	[10.6 - 15.6]	11.18
**'bin.67.fa'**	*Ferrimonas sediminum* DSM 23317	12.5	[9.8 - 15.7]	30.3	[27.9 - 32.8]	12.9	[10.6 - 15.6]	2.17
**'bin.71.fa'**	*Methylogaea oryzae* JCM 16910	12.5	[9.8 - 15.7]	27.3	[24.9 - 29.8]	12.9	[10.6 - 15.6]	5.03
**'bin.34.fa'**	*Quisquiliibacterium transsilvanicum* DSM 29781	12.5	[9.8 - 15.7]	26.1	[23.8 - 28.6]	12.9	[10.6 - 15.6]	11.86
**'bin.7.fa'**	**'bin.34.fa'**	12.5	[9.9 - 15.8]	24.1	[21.8 - 26.6]	12.9	[10.6 - 15.7]	3.03
**'bin.67.fa'**	**'bin.71.fa'**	26.5	[23.2 - 30.2]	21.5	[19.2 - 23.9]	24.4	[21.6 - 27.5]	2.27
**'bin.34.fa'**	**'bin.67.fa'**	13.4	[10.6 - 16.7]	21.5	[19.3 - 24.0]	13.7	[11.3 - 16.5]	1.61
**'bin.7.fa'**	**'bin.67.fa'**	13.3	[10.5 - 16.6]	20.3	[18.1 - 22.7]	13.6	[11.2 - 16.4]	4.64

Query	Subject	d_0	C.I. d_0	d_4	C.I. d_4	d_6	C.I. d_6	Diff. G+C Percent
**'bin.34.fa'**	**'bin.71.fa'**	13.6	[10.8 - 16.9]	20.1	[17.9 - 22.5]	13.9	[11.5 - 16.7]	0.66
**'bin.7.fa'**	**'bin.71.fa'**	12.7	[10.1 - 16.0]	19.1	[17.0 - 21.5]	13.1	[10.8 - 15.9]	2.37
**'bin.7.fa'**	*Caenibacillus caldisaponilyticus* B157	12.5	[9.8 - 15.7]	18.8	[16.6 - 21.1]	12.9	[10.6 - 15.6]	3.09
**'bin.7.fa'**	*Streptomyces carminius* TRM SA0054	12.5	[9.8 - 15.7]	18.8	[16.7 - 21.2]	12.9	[10.6 - 15.6]	18.3
**'bin.7.fa'**	*Streptomyces galbus* DSM 40089	12.5	[9.8 - 15.7]	17.2	[15.1 - 19.5]	12.9	[10.6 - 15.6]	18.22
**'bin.7.fa'**	*Nocardioides agariphilus* KCTC 19276	12.5	[9.8 - 15.7]	17.0	[14.9 - 19.3]	12.9	[10.6 - 15.6]	15.02
**'bin.67.fa'**	*Methylogaea oryzae* JCM 16910	12.5	[9.8 - 15.7]	17.0	[14.9 - 19.4]	12.9	[10.6 - 15.6]	2.76
**'bin.7.fa'**	*Candidatus* Eisenbergiella pullicola CHK197-24098	12.5	[9.8 - 15.8]	16.8	[14.7 - 19.1]	12.9	[10.6 - 15.6]	0.76
**'bin.71.fa'**	*Trichlorobacter ammonificans* G1	12.5	[9.8 - 15.8]	16.4	[14.3 - 18.7]	12.9	[10.6 - 15.6]	4.07
**'bin.67.fa'**	*Candidatus* Eisenbergiella pullicola CHK197-24098	12.5	[9.8 - 15.7]	16.3	[14.3 - 18.6]	12.9	[10.6 - 15.6]	5.39
**'bin.67.fa'**	*Streptosporangium minutum* M26	12.5	[9.8 - 15.7]	16.3	[14.3 - 18.6]	12.9	[10.6 - 15.6]	11.27
**'bin.67.fa'**	*Heliomicrobium gestii* DSM 11169	12.5	[9.9 - 15.8]	16.1	[14.0 - 18.4]	12.9	[10.6 - 15.7]	2.3
**'bin.67.fa'**	*Heliomicrobium gestii* DSM 11169	12.5	[9.9 - 15.8]	16.1	[14.0 - 18.4]	12.9	[10.6 - 15.7]	2.32
**'bin.7.fa'**	*Ruixingdingia sedimenti* LG-4	12.5	[9.8 - 15.7]	15.8	[13.8 - 18.1]	12.9	[10.6 - 15.6]	13.47
**'bin.71.fa'**	*Heliomicrobium gestii* DSM 11169	12.5	[9.8 - 15.8]	15.6	[13.5 - 17.9]	12.9	[10.6 - 15.7]	0.03
**'bin.71.fa'**	*Heliomicrobium gestii* DSM 11169	12.5	[9.8 - 15.8]	15.6	[13.5 - 17.9]	12.9	[10.6 - 15.7]	0.05
**'bin.7.fa'**	*Candidatus* Alectryocaccomicrobium excrementavium 13766	12.5	[9.8 - 15.7]	15.3	[13.2 - 17.5]	12.9	[10.6 - 15.6]	5.03
**'bin.7.fa'**	*Streptomyces pacificus* CWH03	12.5	[9.8 - 15.7]	14.7	[12.7 - 16.9]	12.9	[10.6 - 15.6]	17.46
**'bin.7.fa'**	*Heliomicrobium gestii* DSM 11169	12.5	[9.8 - 15.8]	14.5	[12.6 - 16.8]	12.9	[10.6 - 15.6]	2.32
**'bin.7.fa'**	*Heliomicrobium gestii* DSM 11169	12.5	[9.8 - 15.8]	14.5	[12.6 - 16.8]	12.9	[10.6 - 15.6]	2.34
**'bin.67.fa'**	*Ruixingdingia sedimenti* LG-4	12.5	[9.8 - 15.7]	14.4	[12.4 - 16.6]	12.9	[10.6 - 15.6]	8.84
**'bin.67.fa'**	*Streptomyces galbus* DSM 40089	12.5	[9.8 - 15.7]	14.0	[12.1 - 16.2]	12.9	[10.6 - 15.6]	13.59
**'bin.34.fa'**	*Ferrimonas sediminum* DSM 23317	12.5	[9.8 - 15.7]	13.3	[11.4 - 15.4]	12.9	[10.6 - 15.6]	0.56
**'bin.34.fa'**	*Streptomyces galbus* DSM 40089	12.5	[9.8 - 15.7]	12.5	[10.7 - 14.6]	12.9	[10.6 - 15.6]	15.2
**'bin.7.fa'**	*Thermus aquaticus* DSM 625	12.5	[9.8 - 15.7]	3.7	[2.8 - 4.8]	12.9	[10.6 - 15.6]	13.24
**'bin.67.fa'**	*Hydrogenophaga palleronii* NBRC 102513	12.5	[9.8 - 15.7]	3.7	[2.8 - 4.8]	12.9	[10.6 - 15.6]	7.26

Query	Subject	d_0	C.I. d_0	d_4	C.I. d_4	d_6	C.I. d_6	Diff. G+C Percent
**'bin.34.fa'**	*Deinococcus metallilatus* DSM 105434	12.5	[9.8 - 15.7]	3.7	[2.8 - 4.8]	12.9	[10.6 - 15.6]	10.78
**'bin.34.fa'**	*Granulosicoccus antarcticus* IMCC3135	12.5	[9.8 - 15.7]	3.7	[2.8 - 4.8]	12.9	[10.6 - 15.6]	5.04
**'bin.71.fa'**	*Nocardioides agariphilus* KCTC 19276	12.5	[9.8 - 15.7]	3.7	[2.8 - 4.8]	12.9	[10.6 - 15.6]	12.65
**'bin.67.fa'**	*Shewanella indica* KJW27	12.5	[9.8 - 15.7]	3.7	[2.8 - 4.8]	12.9	[10.6 - 15.6]	7.13
**'bin.71.fa'**	*Granulosicoccus antarcticus* IMCC3135	12.5	[9.8 - 15.7]	3.7	[2.8 - 4.8]	12.9	[10.6 - 15.6]	4.38
**'bin.71.fa'**	*Ruixingdingia sedimenti* LG-4	12.5	[9.8 - 15.7]	3.7	[2.8 - 4.8]	12.9	[10.6 - 15.6]	11.1
**'bin.71.fa'**	*Limnobacter humi* NBRC 111650	12.5	[9.8 - 15.7]	3.7	[2.8 - 4.8]	12.9	[10.6 - 15.6]	1.22
**'bin.71.fa'**	*Shewanella indica* KCTC 23171	12.5	[9.8 - 15.7]	3.7	[2.8 - 4.8]	12.9	[10.6 - 15.6]	4.86
**'bin.71.fa'**	*Candidatus* Blautia merdipullorum 17058	12.5	[9.8 - 15.7]	3.7	[2.8 - 4.8]	12.9	[10.6 - 15.6]	12.22
**'bin.7.fa'**	*Pseudomonas maumuensis* COW77	12.5	[9.8 - 15.7]	3.7	[2.8 - 4.8]	12.9	[10.6 - 15.6]	9.25
**'bin.34.fa'**	*Limnobacter humi* NBRC 111650	12.5	[9.8 - 15.7]	3.7	[2.8 - 4.8]	12.9	[10.6 - 15.6]	1.88
**'bin.67.fa'**	*Quisquiliibacterium transsilvanicum* DSM 29781	12.5	[9.8 - 15.7]	3.7	[2.8 - 4.8]	12.9	[10.6 - 15.6]	10.25
**'bin.34.fa'**	*Pseudomonas brassicae* MAFF 212427	12.5	[9.8 - 15.7]	3.7	[2.8 - 4.8]	12.9	[10.6 - 15.6]	5.52
**'bin.71.fa'**	*Denitrobaculum tricleocarpae* R148T	12.5	[9.8 - 15.7]	3.7	[2.8 - 4.8]	12.9	[10.6 - 15.6]	2.22
**'bin.71.fa'**	*Thermus aquaticus* DSM 625	12.5	[9.8 - 15.7]	3.7	[2.8 - 4.8]	12.9	[10.6 - 15.6]	10.87
**'bin.34.fa'**	*Streptomyces pacificus* CWH03	12.5	[9.8 - 15.7]	3.7	[2.8 - 4.8]	12.9	[10.6 - 15.6]	14.44
**'bin.71.fa'**	*Streptomyces pacificus* CWH03	12.5	[9.8 - 15.7]	3.7	[2.8 - 4.8]	12.9	[10.6 - 15.6]	15.09
**'bin.67.fa'**	*Notoacmeibacter ruber* M2BS9Y-3-1	12.5	[9.8 - 15.7]	3.7	[2.8 - 4.8]	12.9	[10.6 - 15.6]	0.54
**'bin.67.fa'**	*Deinococcus metallilatus* DSM 105434	12.5	[9.8 - 15.7]	3.7	[2.8 - 4.8]	12.9	[10.6 - 15.6]	9.17
**'bin.67.fa'**	*Pseudomonas maumuensis* COW77	12.5	[9.8 - 15.7]	3.7	[2.8 - 4.8]	12.9	[10.6 - 15.6]	4.61
**'bin.7.fa'**	*Hydrogenophaga palleronii* NBRC 102513	12.5	[9.8 - 15.7]	3.7	[2.8 - 4.8]	12.9	[10.6 - 15.6]	11.89
**'bin.67.fa'**	*Deinococcus multiflagellatus* KACC 19287	12.5	[9.8 - 15.7]	3.7	[2.8 - 4.8]	12.9	[10.6 - 15.6]	8.63
**'bin.67.fa'**	*Duganella guangzhouensis* FT80W	12.5	[9.8 - 15.7]	3.7	[2.8 - 4.8]	12.9	[10.6 - 15.6]	2.94
**'bin.7.fa'**	*Deinococcus metallilatus* DSM 105434	12.5	[9.8 - 15.7]	3.7	[2.8 - 4.8]	12.9	[10.6 - 15.6]	13.81
**'bin.34.fa'**	*Trichlorobacter ammonificans* G1	12.5	[9.8 - 15.7]	3.7	[2.8 - 4.8]	12.9	[10.6 - 15.6]	3.41
**'bin.34.fa'**	*Brenneria goodwinii* FRB 141	12.5	[9.8 - 15.7]	3.7	[2.8 - 4.8]	12.9	[10.6 - 15.6]	4.82
**'bin.34.fa'**	*Denitrobaculum tricleocarpae* R148T	12.5	[9.8 - 15.7]	3.7	[2.8 - 4.8]	12.9	[10.6 - 15.6]	1.56

Query	Subject	d_0	C.I. d_0	d_4	C.I. d_4	d_6	C.I. d_6	Diff. G+C Percent
**'bin.7.fa'**	*Shewanella indica* KCTC 23171	12.5	[9.8 - 15.7]	3.7	[2.8 - 4.8]	12.9	[10.6 - 15.6]	2.49
**'bin.34.fa'**	*Duganella guangzhouensis* FT80W	12.5	[9.8 - 15.7]	3.7	[2.8 - 4.8]	12.9	[10.6 - 15.6]	4.55
**'bin.34.fa'**	*Heliomicrobium gestii* DSM 11169	12.5	[9.8 - 15.7]	3.7	[2.8 - 4.8]	12.9	[10.6 - 15.6]	0.71
**'bin.71.fa'**	*Streptomyces carminius* TRM SA0054	12.5	[9.8 - 15.7]	3.7	[2.8 - 4.8]	12.9	[10.6 - 15.6]	15.93
**'bin.34.fa'**	*Shewanella indica* KJW27	12.5	[9.8 - 15.7]	3.7	[2.8 - 4.8]	12.9	[10.6 - 15.6]	5.52
**'bin.67.fa'**	*Brenneria goodwinii* FRB 141	12.5	[9.8 - 15.7]	3.7	[2.8 - 4.8]	12.9	[10.6 - 15.6]	6.43
**'bin.71.fa'**	*Pseudomonas brassicae* MAFF 212427	12.5	[9.8 - 15.7]	3.7	[2.8 - 4.8]	12.9	[10.6 - 15.6]	6.18
**'bin.7.fa'**	*Limnobacter humi* NBRC 111650	12.5	[9.8 - 15.7]	3.7	[2.8 - 4.8]	12.9	[10.6 - 15.6]	1.15
**'bin.7.fa'**	*Deinococcus multiflagellatus* KACC 19287	12.5	[9.8 - 15.7]	3.7	[2.8 - 4.8]	12.9	[10.6 - 15.6]	13.27
**'bin.67.fa'**	*Denitrobaculum tricleocarpae* R148T	12.5	[9.8 - 15.7]	3.7	[2.8 - 4.8]	12.9	[10.6 - 15.6]	0.05
**'bin.67.fa'**	*Subtercola boreus* JCM 11267	12.5	[9.8 - 15.7]	3.7	[2.8 - 4.8]	12.9	[10.6 - 15.6]	8.48
**'bin.71.fa'**	*Streptomyces galbus* DSM 40089	12.5	[9.8 - 15.7]	3.7	[2.8 - 4.8]	12.9	[10.6 - 15.6]	15.85
**'bin.34.fa'**	*Pseudonocardia asaccharolytica* NBRC 16224	12.5	[9.8 - 15.7]	3.7	[2.8 - 4.8]	12.9	[10.6 - 15.6]	13.9
**'bin.34.fa'**	*Candidatus* Blautia merdipullorum 17058	12.5	[9.8 - 15.7]	3.7	[2.8 - 4.8]	12.9	[10.6 - 15.6]	12.88
**'bin.34.fa'**	*Paracoccus luteus* CFH 10530	12.5	[9.8 - 15.7]	3.7	[2.8 - 4.8]	12.9	[10.6 - 15.6]	11.7
**'bin.7.fa'**	*Tersicoccus phoenicis* 1P05MA	12.5	[9.8 - 15.7]	3.7	[2.8 - 4.8]	12.9	[10.6 - 15.6]	15.8
**'bin.71.fa'**	*Pseudonocardia asaccharolytica* NBRC 16224	12.5	[9.8 - 15.7]	3.7	[2.8 - 4.8]	12.9	[10.6 - 15.6]	14.56
**'bin.71.fa'**	*Subtercola boreus* JCM 11267	12.5	[9.8 - 15.7]	3.7	[2.8 - 4.8]	12.9	[10.6 - 15.6]	10.74
**'bin.7.fa'**	*Pseudonocardia asaccharolytica* NBRC 16224	12.5	[9.8 - 15.7]	3.7	[2.8 - 4.8]	12.9	[10.6 - 15.6]	16.93
**'bin.34.fa'**	*Candidatus* Alectryocaccomicrobium excrementavium 13766	12.5	[9.8 - 15.7]	3.7	[2.8 - 4.8]	12.9	[10.6 - 15.6]	2.01
**'bin.71.fa'**	*Notoacmeibacter ruber* M2BS9Y-3-1	12.5	[9.8 - 15.7]	3.7	[2.8 - 4.8]	12.9	[10.6 - 15.6]	2.8
**'bin.34.fa'**	*Deinococcus multiflagellatus* KACC 19287	12.5	[9.8 - 15.7]	3.7	[2.8 - 4.8]	12.9	[10.6 - 15.6]	10.24
**'bin.7.fa'**	*Pulveribacter suum* KACC 19309T	12.5	[9.8 - 15.7]	3.7	[2.8 - 4.8]	12.9	[10.6 - 15.6]	14.21
**'bin.67.fa'**	*Candidatus* Blautia merdipullorum 17058	12.5	[9.8 - 15.7]	3.7	[2.8 - 4.8]	12.9	[10.6 - 15.6]	14.49
**'bin.71.fa'**	*Streptosporangium minutum* M26	12.5	[9.8 - 15.7]	3.7	[2.8 - 4.8]	12.9	[10.6 - 15.6]	13.54

Query	Subject	d_0	C.I. d_0	d_4	C.I. d_4	d_6	C.I. d_6	Diff. G+C Percent
**'bin.67.fa'**	*Caenibacillus caldisaponilyticus* B157	12.5	[9.8 - 15.7]	3.7	[2.8 - 4.8]	12.9	[10.6 - 15.6]	7.73
**'bin.34.fa'**	*Haliea alexandrii* LZ-16-2T	12.5	[9.8 - 15.7]	3.7	[2.8 - 4.8]	12.9	[10.6 - 15.6]	3.37
**'bin.34.fa'**	*Notoacmeibacter ruber* M2BS9Y-3-1	12.5	[9.8 - 15.7]	3.7	[2.8 - 4.8]	12.9	[10.6 - 15.6]	2.15
**'bin.71.fa'**	*Pulveribacter suum* KACC 19309T	12.5	[9.8 - 15.7]	3.7	[2.8 - 4.8]	12.9	[10.6 - 15.6]	11.84
**'bin.71.fa'**	*Tersicoccus phoenicis* 1P05MA	12.5	[9.8 - 15.7]	3.7	[2.8 - 4.8]	12.9	[10.6 - 15.6]	13.43
**'bin.7.fa'**	*Notoacmeibacter ruber* M2BS9Y-3-1	12.5	[9.8 - 15.7]	3.7	[2.8 - 4.8]	12.9	[10.6 - 15.6]	5.17
**'bin.34.fa'**	*Tersicoccus phoenicis* 1P05MA	12.5	[9.8 - 15.7]	3.7	[2.8 - 4.8]	12.9	[10.6 - 15.6]	12.78
**'bin.34.fa'**	*Streptosporangium minutum* M26	12.5	[9.8 - 15.7]	3.7	[2.8 - 4.8]	12.9	[10.6 - 15.6]	12.88
**'bin.7.fa'**	*Streptosporangium minutum* M26	12.5	[9.8 - 15.7]	3.7	[2.8 - 4.8]	12.9	[10.6 - 15.6]	15.91
**'bin.34.fa'**	*Subtercola boreus* JCM 11267	12.5	[9.8 - 15.7]	3.7	[2.8 - 4.8]	12.9	[10.6 - 15.6]	10.09
**'bin.34.fa'**	*Hydrogenophaga palleronii* NBRC 102513	12.5	[9.8 - 15.7]	3.7	[2.8 - 4.8]	12.9	[10.6 - 15.6]	8.87
**'bin.71.fa'**	*Haliea alexandrii* LZ-16-2T	12.5	[9.8 - 15.7]	3.7	[2.8 - 4.8]	12.9	[10.6 - 15.6]	4.03
**'bin.34.fa'**	*Pelolinea submarina* DSM 23923	12.5	[9.8 - 15.7]	3.7	[2.8 - 4.8]	12.9	[10.6 - 15.6]	7.28
**'bin.71.fa'**	*Ferrimonas sediminum* DSM 23317	12.5	[9.8 - 15.7]	3.7	[2.8 - 4.8]	12.9	[10.6 - 15.6]	0.09
**'bin.34.fa'**	*Mycolicibacterium aurantiacum* 3033	12.5	[9.8 - 15.7]	3.7	[2.8 - 4.8]	12.9	[10.6 - 15.6]	10.21
**'bin.34.fa'**	*Halopseudomonas salegens* CECT 8338	12.5	[9.8 - 15.7]	3.7	[2.8 - 4.8]	12.9	[10.6 - 15.6]	0.2
**'bin.71.fa'**	*Candidatus* Eisenbergiella pullicola CHK197-24098	12.5	[9.8 - 15.7]	3.7	[2.8 - 4.8]	12.9	[10.6 - 15.6]	3.13
**'bin.71.fa'**	*Duganella guangzhouensis* FT80W	12.5	[9.8 - 15.7]	3.7	[2.8 - 4.8]	12.9	[10.6 - 15.6]	5.2
**'bin.67.fa'**	*Tersicoccus phoenicis* 1P05MA	12.5	[9.8 - 15.7]	3.7	[2.8 - 4.8]	12.9	[10.6 - 15.6]	11.17
**'bin.67.fa'**	*Thermus aquaticus* DSM 625	12.5	[9.8 - 15.7]	3.7	[2.8 - 4.8]	12.9	[10.6 - 15.6]	8.6
**'bin.71.fa'**	*Brenneria goodwinii* FRB 141	12.5	[9.8 - 15.7]	3.7	[2.8 - 4.8]	12.9	[10.6 - 15.6]	4.16
**'bin.7.fa'**	*Quisquiliibacterium transsilvanicum* DSM 29781	12.5	[9.8 - 15.7]	3.7	[2.8 - 4.8]	12.9	[10.6 - 15.6]	14.89
**'bin.7.fa'**	*Shewanella indica* KJW27	12.5	[9.8 - 15.7]	3.7	[2.8 - 4.8]	12.9	[10.6 - 15.6]	2.5
**'bin.71.fa'**	*Caenibacillus caldisaponilyticus* B157	12.5	[9.8 - 15.7]	3.7	[2.8 - 4.8]	12.9	[10.6 - 15.6]	5.46
**'bin.34.fa'**	*Caenibacillus caldisaponilyticus* B157	12.5	[9.8 - 15.7]	3.7	[2.8 - 4.8]	12.9	[10.6 - 15.6]	6.12
**'bin.7.fa'**	*Paracoccus luteus* CFH 10530	12.5	[9.8 - 15.7]	3.7	[2.8 - 4.8]	12.9	[10.6 - 15.6]	14.73
**'bin.34.fa'**	*Nocardioides agariphilus* KCTC 19276	12.5	[9.8 - 15.7]	3.7	[2.8 - 4.8]	12.9	[10.6 - 15.6]	11.99

Query	Subject	d_0	C.I. d_0	d_4	C.I. d_4	d_6	C.I. d_6	Diff. G+C Percent
**'bin.34.fa'**	*Shewanella indica* KCTC 23171	12.5	[9.8 - 15.7]	3.7	[2.8 - 4.8]	12.9	[10.6 - 15.6]	5.52
**'bin.71.fa'**	*Deinococcus metallilatus* DSM 105434	12.5	[9.8 - 15.7]	3.7	[2.8 - 4.8]	12.9	[10.6 - 15.6]	11.44
**'bin.67.fa'**	*Pseudonocardia asaccharolytica* NBRC 16224	12.5	[9.8 - 15.7]	3.7	[2.8 - 4.8]	12.9	[10.6 - 15.6]	12.29
**'bin.34.fa'**	*Methylogaea oryzae* JCM 16910	12.5	[9.8 - 15.7]	3.7	[2.8 - 4.8]	12.9	[10.6 - 15.6]	4.37
**'bin.34.fa'**	*Heliomicrobium gestii* DSM 11169	12.5	[9.8 - 15.7]	3.7	[2.8 - 4.8]	12.9	[10.6 - 15.6]	0.69
**'bin.71.fa'**	*Deinococcus multiflagellatus* KACC 19287	12.5	[9.8 - 15.7]	3.7	[2.8 - 4.8]	12.9	[10.6 - 15.6]	10.9
**'bin.67.fa'**	*Pulveribacter suum* KACC 19309T	12.5	[9.8 - 15.7]	3.7	[2.8 - 4.8]	12.9	[10.6 - 15.6]	9.57
**'bin.7.fa'**	*Brenneria goodwinii* FRB 141	12.5	[9.8 - 15.7]	3.7	[2.8 - 4.8]	12.9	[10.6 - 15.6]	1.79
**'bin.67.fa'**	*Limnobacter humi* NBRC 111650	12.5	[9.8 - 15.7]	3.7	[2.8 - 4.8]	12.9	[10.6 - 15.6]	3.49
**'bin.67.fa'**	*Haliea alexandrii* LZ-16-2T	12.5	[9.8 - 15.7]	3.7	[2.8 - 4.8]	12.9	[10.6 - 15.6]	1.76
**'bin.67.fa'**	*Pelolinea submarina* DSM 23923	12.5	[9.8 - 15.7]	3.7	[2.8 - 4.8]	12.9	[10.6 - 15.6]	8.89
**'bin.71.fa'**	*Pseudomonas panipatensis* CCM 7469	12.5	[9.8 - 15.7]	3.7	[2.8 - 4.8]	12.9	[10.6 - 15.6]	8.32
**'bin.7.fa'**	*Duganella guangzhouensis* FT80W	12.5	[9.8 - 15.7]	3.7	[2.8 - 4.8]	12.9	[10.6 - 15.6]	7.57
**'bin.7.fa'**	*Mycolicibacterium aurantiacum* 3033	12.5	[9.8 - 15.7]	3.7	[2.8 - 4.8]	12.9	[10.6 - 15.6]	13.23
**'bin.67.fa'**	*Streptomyces pacificus* CWH03	12.5	[9.8 - 15.7]	3.7	[2.8 - 4.8]	12.9	[10.6 - 15.6]	12.83
**'bin.34.fa'**	*Pseudomonas maumuensis* COW77	12.5	[9.8 - 15.7]	3.7	[2.8 - 4.8]	12.9	[10.6 - 15.6]	6.22
**'bin.7.fa'**	*Ferrimonas sediminum* DSM 23317	12.5	[9.8 - 15.7]	3.7	[2.8 - 4.8]	12.9	[10.6 - 15.6]	2.46
**'bin.71.fa'**	*Halopseudomonas salegens* CECT 8338	12.5	[9.8 - 15.7]	3.7	[2.8 - 4.8]	12.9	[10.6 - 15.6]	0.45
**'bin.67.fa'**	*Streptomyces carminius* TRM SA0054	12.5	[9.8 - 15.7]	3.7	[2.8 - 4.8]	12.9	[10.6 - 15.6]	13.66
**'bin.71.fa'**	*Pelolinea submarina* DSM 23923	12.5	[9.8 - 15.7]	3.7	[2.8 - 4.8]	12.9	[10.6 - 15.6]	6.62
**'bin.67.fa'**	*Shewanella indica* KCTC 23171	12.5	[9.8 - 15.7]	3.7	[2.8 - 4.8]	12.9	[10.6 - 15.6]	7.13
**'bin.71.fa'**	*Hydrogenophaga palleronii* NBRC 102513	12.5	[9.8 - 15.7]	3.7	[2.8 - 4.8]	12.9	[10.6 - 15.6]	9.52
**'bin.71.fa'**	*Quisquiliibacterium transsilvanicum* DSM 29781	12.5	[9.8 - 15.7]	3.7	[2.8 - 4.8]	12.9	[10.6 - 15.6]	12.52
**'bin.71.fa'**	*Pseudomonas maumuensis* COW77	12.5	[9.8 - 15.7]	3.7	[2.8 - 4.8]	12.9	[10.6 - 15.6]	6.88
**'bin.34.fa'**	*Thermus aquaticus* DSM 625	12.5	[9.8 - 15.7]	3.7	[2.8 - 4.8]	12.9	[10.6 - 15.6]	10.21
**'bin.7.fa'**	*Candidatus* Blautia merdipullorum 17058	12.5	[9.8 - 15.7]	3.7	[2.8 - 4.8]	12.9	[10.6 - 15.6]	9.85

Query	Subject	d_0	C.I. d_0	d_4	C.I. d_4	d_6	C.I. d_6	Diff. G+C Percent
**'bin.34.fa'**	*Pseudomonas panipatensis* CCM 7469	12.5	[9.8 - 15.7]	3.7	[2.8 - 4.8]	12.9	[10.6 - 15.6]	7.66
**'bin.71.fa'**	*Paracoccus luteus* CFH 10530	12.5	[9.8 - 15.7]	3.7	[2.8 - 4.8]	12.9	[10.6 - 15.6]	12.36
**'bin.7.fa'**	*Haliea alexandrii* LZ-16-2T	12.5	[9.8 - 15.7]	3.7	[2.8 - 4.8]	12.9	[10.6 - 15.6]	6.4
**'bin.7.fa'**	*Subtercola boreus* JCM 11267	12.5	[9.8 - 15.7]	3.7	[2.8 - 4.8]	12.9	[10.6 - 15.6]	13.11
**'bin.34.fa'**	*Candidatus* Eisenbergiella pullicola CHK197-24098	12.5	[9.8 - 15.7]	3.7	[2.8 - 4.8]	12.9	[10.6 - 15.6]	3.78
**'bin.34.fa'**	*Streptomyces carminius* TRM SA0054	12.5	[9.8 - 15.7]	3.7	[2.8 - 4.8]	12.9	[10.6 - 15.6]	15.27
**'bin.34.fa'**	*Ruixingdingia sedimenti* LG-4	12.5	[9.8 - 15.7]	3.7	[2.8 - 4.8]	12.9	[10.6 - 15.6]	10.45
**'bin.7.fa'**	*Pelolinea submarina* DSM 23923	12.5	[9.8 - 15.7]	3.7	[2.8 - 4.8]	12.9	[10.6 - 15.6]	4.25
**'bin.7.fa'**	*Denitrobaculum tricleocarpae* R148T	12.5	[9.8 - 15.7]	3.7	[2.8 - 4.8]	12.9	[10.6 - 15.6]	4.59
**'bin.7.fa'**	*Granulosicoccus antarcticus* IMCC3135	12.5	[9.8 - 15.7]	3.7	[2.8 - 4.8]	12.9	[10.6 - 15.6]	2.01
**'bin.67.fa'**	*Granulosicoccus antarcticus* IMCC3135	12.5	[9.8 - 15.7]	3.7	[2.8 - 4.8]	12.9	[10.6 - 15.6]	6.65
**'bin.71.fa'**	*Shewanella indica* KJW27	12.5	[9.8 - 15.7]	3.7	[2.8 - 4.8]	12.9	[10.6 - 15.6]	4.86
**'bin.67.fa'**	*Nocardioides agariphilus* KCTC 19276	12.5	[9.8 - 15.7]	3.7	[2.8 - 4.8]	12.9	[10.6 - 15.6]	10.38
**'bin.67.fa'**	*Paracoccus luteus* CFH 10530	12.5	[9.8 - 15.7]	3.7	[2.8 - 4.8]	12.9	[10.6 - 15.6]	10.09

Table 4: Strains in your dataset

Joint dataset of automatically determined closest type strains (if this mode was chosen), manually selected type strains (if selected accordingly) and the provided user strains, if provided (marked in yellow).

Strain	Authority	Other deposits	Synonyms	Base pairs	Percent G+C	No. proteins	Goldstamp	Bioproject accession	Biosample accession	Assembly accession	IMG OID
Streptomyces carminius TRM SA0054	Wang et al. 2018	KCTC 39903; CCTCC AA 2016041	*Streptomyces carminius*	7197 105	73.2	6110	Gp0365186	PRJNA417743	SAMN08005496	GCA_002794255	
Paracoccus luteus CFH 10530	Ming et al. 2020	CGMCC 1.16597; KCTC 62919	*Paracoccus luteus*	3324 847	69.6	3143		PRJNA513413	SAMN10712550	GCA_004522155	
Shewanella indica KJW27	Verma et al. 2011 emend. Thorell et al. 2019	KCTC 23171; BCC 41031; DSM 23486; NCIM 5388; KJW27	*Shewanella indica*	4402 639	52.4	3787	Gp0372229	PRJNA224116	SAMN07175133	GCF_002836975	
Limnobacter humi NBRC 111650	Nguyen and Kim 2017	KACC 18574; UCM 39	*Limnobacter humi*	3231 595	56.0	3020		PRJNA224116	SAMN30025452	GCF_024518835	
Heliomicrobium gestii DSM 11169	(Ormerod et al. 1996) Kyndt et al. 2021	ATCC 43375; Chainat	*Heliobacterium gestii; Heliomicrobium gestii*	3694 627	57.2	3388	Gp0505730				2901048070
Mycolicibacterium aurantiacum 3033	Pan et al. 2022	MCCC 1K04526; KCTC 49712; B 3033	*Mycolicibacterium aurantiacum*	1101 1331	68.1	10263		PRJNA597954	SAMN13693881	GCA_019710595	
Methylogaea oryzae JCM 16910	Geymonat et al. 2011	DSM 23452; E10	*Methylogaea oryzae*	3350 496	62.3	2515	Gp0093147	PRJNA224116	SAMD00000538	GCF_001312345	
Candidatus Alectryocaccomicrobium excrementavium 13766	Gilroy et al. 2021		*Candidatus* Alectryocaccomicrobium excrementavium	3048 624	59.9	2751		PRJNA543206	SAMN15816965	GCA_018716755	

Strain	Authority	Other deposits	Synonyms	Base pairs	Percent G+C	No. proteins	Goldstamp	Bioproject accession	Biosample accession	Assembly accession	IMG OID
Candidatus Blautia merdipullorum 17058	Gilroy et al. 2021		*Candidatus* Blautia merdipullorum	3667 621	45.0	3506		PRJNA543206	SAMN15816655	GCA_019118685	
Candidatus Eisenbergiella pullicola CHK197-24098	Gilroy et al. 2021		*Candidatus* Eisenbergiella pullicola	2909 585	54.1	2596		PRJNA543206	SAMN15816836	GCA_019117385	
Deinococcus multiflagellatus KACC 19287	Kim et al. 2018	NBRC 112888; ID1504	*Deinococcus multiflagellatus*	4762 367	68.1	4542		PRJNA224116	SAMN20982218	GCA_020166415	
Pseudomonas maumuensis COW77	Girard et al. 2022	CFBP 8888; LMG 32179	*Pseudomonas maumuensis*	5758 328	64.1	4970		PRJNA639797	SAMN19473670	GCA_019139675	
Trichlorobacter ammonificans G1	Sorokin et al. 2023	DSM 105480; UNIQEM 10005; UNIQEM U1005	*Trichlorobacter ammonificans*	3058 983	61.3	2731		PRJNA224116	SAMEA13094085	GCF_933509905	
Deinococcus metallilatus DSM 105434	Kim et al. 2015	DSM 105434; NBRC 110141; KACC 17964; MA1002	*Deinococcus metallilatus*	4387 729	68.7	4314	Gp0457725				2861326389
Quisquiliibacterium transsilvanicum DSM 29781	Felföldi et al. 2017	JCM 31785; CGI-09	*Quisquiliibacterium transsilvanicum*	4254 555	69.8	3946	Gp0456329				2861366533
Pseudonocardia asaccharolytica NBRC 16224	Reichert et al. 1998 emend. Nouioui et al. 2018	580; CIP 105685; DSM 44247; JCM 10410; IFO 16224; NBRC 16224	*Pseudonocardia asaccharolytica*	4995 400	71.8	4883	Gp0024446	PRJDB436	SAMD00166238	GCA_007989065	
Nocardioides agariphilus KCTC 19276	Dastager et al. 2008	DSM 19323; JCM 16020; MSL 28	*Nocardioides agariphilus*	4736 498	69.9	4442		PRJNA674498	SAMN16659058	GCA_015352445	

Strain	Authority	Other deposits	Synonyms	Base pairs	Percent G+C	No. proteins	Goldstamp	Bioproject accession	Biosample accession	Assembly accession	IMG OID
Notoacmeibacter ruber M2BS9Y-3-1	Yan and Tuo 2020	CGMCC 1.13746; KCTC 62838	*Notoacmeibacter ruber*	3254 790	60.0	3033		PRJNA495143	SAMN10220050	GCA_003668555	
Tersicoccus phoenicis 1P05MA	Vaishampayan et al. 2013	NRRL B-59547; DSM 30849	*Tersicoccus phoenicis*	3209 563	70.7	2873	Gp0316497	PRJNA347610	SAMN05893361	GCA_001968835	
Caenibacillus caldisaponilyticus B157	Tsujimoto et al. 2016	DSM 101100; NBRC 111400	*Caenibacillus caldisaponilyticus*	3356 095	51.8	3132	Gp0316165	PRJDB4782	SAMD00051327	GCA_002003465	
Subtercola boreus JCM 11267	Männistö et al. 2000	CIP 106947; ATCC BAA-168; CCUG 43135; DSM 13056; JCM 11267; NBRC 103085; K300	*Subtercola boreus*	4212 750	68.0	3779	Gp0365333	PRJNA378458	SAMN06610376	GCA_003399685	
Pulveribacter suum KACC 19309T	Heo et al. 2019	NBRC 113102; SC2-7	*Pulveribacter suum*	3358 427	69.1	2970		PRJNA438161	SAMN08707401	GCA_003013695	
Pelolinea submarina DSM 23923	Imachi et al. 2014	DSM 28969; JCM 17238; MO-CFX1	*Pelolinea submarina*	3518 991	50.6	3095	Gp0290646	PRJNA463391	SAMN09074695	GCA_003385075	2770939606

Strain	Authority	Other deposits	Synonyms	Base pairs	Percent G+C	No. proteins	Goldstamp	Bioproject accession	Biosample accession	Assembly accession	IMG OID
Streptomyces galbus DSM 40089	Frommer 1959	BCRC 12166; CCRC 12166; NRRL B-2283; NRRL ISP-5089; NCIMB 13005; CBS 831.68; ATCC 23910; DSM 40089; JCM 4222; JCM 4639; IFO 12864; NBRC 12864; VKM Ac-165; IMET 42937; RIA 1121	*Streptomyces galbus*	7798 215	73.1	6984		PRJNA224116	SAMN11280916	GCF_005280195	
Denitrobaculum tricleocarpae R148T	Wang et al. 2020	MCCC 1K03781; KCTC 72137	*Denitrobaculum tricleocarpae*	6431 720	59.5	5799		PRJNA550178	SAMN12109841	GCA_007004665	
Granulosicoccus antarcticus IMCC3135	Lee et al. 2008	DSM 24912; NBRC 102684; KCCM 42676; IMCC3135r	*Granulosicoccus antarcticus*	7783 862	52.9	6761	Gp0021679	PRJNA167143	SAMN06112367	GCA_002215215	
Brenneria goodwinii FRB 141	Denman et al. 2012	LMG 26270; NCPPB 4484; BCC 845; DSM 27058; R-43656	*Brenneria goodwinii*	5360 730	53.1	4528	Gp0302355	PRJNA308225	SAMN04388496	GCA_002291445	
Ferrimonas sediminum DSM 23317	Ji et al. 2013	LMG 25564; JYr13	*Ferrimonas sediminum*	4370 844	57.3	3940	Gp0116521	PRJNA303718	SAMN04488540	GCA_900100175	2634166324
Duganella guangzhouensis FT80W	Lu et al. 2020	GDMCC 1.1678; KACC 21470	*Duganella guangzhouensis*	7540 741	62.4	6591		PRJNA575502	SAMN13220417	GCA_009674535	

Strain	Authority	Other deposits	Synonyms	Base pairs	Percent G+C	No. proteins	Goldstamp	Bioproject accession	Biosample accession	Assembly accession	IMG OID
Ruixingdingia sedimenti LG-4	Xu et al. 2024	MCCC 1K08849; KCTC 8136	*Ruixingdingia sedimenti*	4686139	68.3	4593		PRJNA1012573	SAMN37264443	GCA_031432425	
Thermus aquaticus DSM 625	Brock and Freeze 1969 emend. García-López et al. 2019	LMG 8924; NCIMB 11243; ATCC 25104; JCM 10724; NBRC 103206; NCAIM B.01703; NCIB 11243; YT-1	*Thermus aquaticus*	2216193	68.1	2348	Gp0124680	PRJNA291648	SAMN03951125	GCA_001280255	
Pseudomonas brassicae MAFF 212427	Sawada et al. 2020	ICMP 23635	*Pseudomonas brassicae*	5452514	63.4	4228		PRJNA605507	SAMN14069736	GCA_010671725	
Hydrogenophaga palleronii NBRC 102513	(Davis 1970) Willems et al. 1989	CFBP 2445; LMG 2366; CIP 103304; ATCC 17724; CCUG 1780; CCUG 20334; DSM 63; JCM 21412; VKM B-1328; Stanier 362t1	*Hydrogenophaga palleronii*; *Pseudomonas palleronii*	4841746	66.8	4489	Gp0075788	PRJDB1376	SAMD00046729	GCA_001571225	
Shewanella indica KCTC 23171	Verma et al. 2011 emend. Thorell et al. 2019	KCTC 23171; BCC 41031; DSM 23486; NCIM 5388; KJW27	*Shewanella indica*	4386546	52.4	3880		PRJDB10508	SAMD00245601	GCA_014652115	
Pseudomonas panipatensis CCM 7469	Gupta et al. 2008	DSM 21819; Esp-1; MTCC 8990	*Pseudomonas panipatensis*	5775045	65.6	5273	Gp0127172	PRJEB15946	SAMN05216272	GCA_900099785	

Strain	Authority	Other deposits	Synonyms	Base pairs	Percent G+C	No. proteins	Goldstamp	Bioproject accession	Biosample accession	Assembly accession	IMG OID
Halopseudomonas salegens CECT 8338	(Amoozegar et al. 2014) Rudra and Gupta 2021	GBPy5; IBRC M 10762	*Halopseudomonas salegens*; *Neopseudomonas salegens*; *Pseudomonas salegens*	3796005	57.7	3431	Gp0127212	PRJEB16488	SAMN05216210	GCA_900105655	
Streptomyces pacificus CWH03	Takahashi et al. 2023	TBRC 15780; NBRC 114659	*Streptomyces pacificus*	6626316	72.3	5800		PRJDB9281	SAMD00204451	GCA_011766325	
Haliea alexandrii LZ-16-2T	Yang et al. 2020	KCTC 62344; CCTCC AB 2017229	*Haliea alexandrii*	3961377	61.3	3588		PRJNA224116	SAMN10169376	GCF_003719295	
Streptosporangium minutum M26	Le Roes-Hill et al. 2021	LMG 28850; NRRL B-65295	*Streptosporangium minutum*	9565337	70.8	8393	Gp0371850	PRJNA386254	SAMN06928586	GCA_002149035	
Heliomicrobium gestii DSM 11169	(Ormerod et al. 1996) Kyndt et al. 2021	ATCC 43375; Chainat	*Heliobacterium gestii*; *Heliomicrobium gestii*	3678836	57.2	3281		PRJNA599378	SAMN13746817	GCA_009877435	
bin.7.fa				2864993	54.9	2912					
bin.34.fa				625575	57.9	753					
bin.67.fa				1929198	59.5	2027					
bin.71.fa				1845766	57.2	2039					

Methods, Results and References

The genome sequence data were uploaded to the Type (Strain) Genome Server (TYGS), a free bioinformatics platform available under https://tygs.dsmz.de, for a whole genome-based taxonomic analysis [1]. The analysis also made use of recently introduced methodological updates and features [2]. Information on nomenclature, synonymy and associated taxonomic literature was provided by TYGS's sister database, the List of Prokaryotic names with Standing in Nomenclature (LPSN, available at https://lpsn.dsmz.de) [2]. The results were provided by the TYGS on 2024-06-16. The TYGS analysis was subdivided into the following steps:

Determination of closely related type strains

The determination of closely related type strains did not succeed because not a single 16S rDNA gene sequence was detected in the provided user genomes. The subsequent analyses are thus only based on the provided genome data and the manually selected type strains, if any.

Pairwise comparison of genome sequences

For the phylogenomic inference, all pairwise comparisons among the set of genomes were conducted using GBDP and accurate intergenomic distances inferred under the algorithm 'trimming' and distance formula d_5 [3]. 100 distance replicates were calculated each. Digital DDH values and confidence intervals were calculated using the recommended settings of the GGDC 4.0 [2,3].

Phylogenetic inference

The resulting intergenomic distances were used to infer a balanced minimum evolution tree with branch support via FASTME 2.1.6.1 including SPR postprocessing [4]. Branch support was inferred from 100 pseudo-bootstrap replicates each. The trees were rooted at the midpoint [5] and visualized with PhyD3 [6].

Type-based species and subspecies clustering

The type-based species clustering using a 70% dDDH radius around each of the 40 type strains was done as previously described [1]. The resulting groups are shown in Table 1 and 4. Subspecies clustering was done using a 79% dDDH threshold as previously introduced [7].

Results

Type-based species and subspecies clustering

The resulting species and subspecies clusters are listed in Table 4, whereas the taxonomic identification of the query strains is found in Table 1. Briefly, the clustering yielded 41 species clusters and the provided query strains were assigned to 4 of these. Moreover, user strains were located in 4 of 42 subspecies clusters.

Figure caption genome tree

Figure 2. Tree inferred with FastME 2.1.6.1 [4] from GBDP distances calculated from genome sequences. The branch lengths are scaled in terms of GBDP distance formula d_5. The numbers above branches are GBDP pseudo-bootstrap support values > 60 % from 100 replications, with an average branch support of 16.8 %. The tree was rooted at the midpoint [5].

References

[1] Meier-Kolthoff JP, Göker M. TYGS is an automated high-throughput platform for state-of-the-art genome-based taxonomy. Nat. Commun. 2019;10: 2182. DOI: 10.1038/s41467-019-10210-3

[2] Meier-Kolthoff JP, Sardà Carbasse J, Peinado-Olarte RL, Göker M. TYGS and LPSN: a database tandem for fast and reliable genome-based classification and nomenclature of prokaryotes. Nucleic Acid Res. 2022;50: D801–D807. DOI: 10.1093/nar/gkab902

[3] Meier-Kolthoff JP, Auch AF, Klenk H-P, Göker M. Genome sequence-based species delimitation with confidence intervals and improved distance functions. BMC Bioinformatics. 2013;14: 60. DOI: 10.1186/1471-2105-14-60

[4] Lefort V, Desper R, Gascuel O. FastME 2.0: A comprehensive, accurate, and fast distance-based phylogeny inference program. Mol Biol Evol. 2015;32: 2798–2800. DOI: 10.1093/molbev/msv150

[5] Farris JS. Estimating phylogenetic trees from distance matrices. Am Nat. 1972;106: 645–667.

[6] Kreft L, Botzki A, Coppens F, Vandepoele K, Van Bel M. PhyD3: A phylogenetic tree viewer with extended phyloXML support for functional genomics data visualization. Bioinformatics. 2017;33: 2946–2947. DOI: 10.1093/bioinformatics/btx324

[7] Meier-Kolthoff JP, Hahnke RL, Petersen J, Scheuner C, Michael V, Fiebig A, et al. Complete genome sequence of DSM 30083^{T}, the type strain (U5/41^{T}) of *Escherichia coli*, and a proposal for delineating subspecies in microbial taxonomy. Stand Genomic Sci. 2014;9: 2. DOI: 10.1186/1944-3277-9-2
